# Metabolic dysfunction–associated steatohepatitis exacerbated by *Clostridium perfringens*–derived ammonia is attenuated by tripeptide DT-109

**DOI:** 10.1172/JCI200522

**Published:** 2026-05-12

**Authors:** Pengxiang Qu, Shusi Ding, Yanru Zhang, Yang Zhao, Erfei Song, Liangshuo Hu, Ruike Ding, Wenbin Cao, Yiting Hou, Jia Qi, Juan Zhao, Chenjing Duan, Shuangqing Liu, Chong Shen, Ying Zhao, Yanhong Guo, Zuowen Zheng, Shiwei Luo, Huizhong Hu, Liang Bai, Sihai Zhao, Bo Wang, Shuixiang He, Yi Wu, Xuelian Xiong, Qiutong Wu, Weiwang Gu, Oren Rom, Aimin Xu, Lemin Zheng, Jifeng Zhang, Enqi Liu, Y. Eugene Chen

**Affiliations:** 1 Laboratory Animal Center, School of Basic Medical Science, Xi’an Jiaotong University; Department of Gastroenterology, The First Affiliated Hospital of Xi’an Jiaotong University; Key Laboratory of Environment and Genes Related to Diseases, Ministry of Education of China, Xi’an, China.; 2 Beijing Tiantan Hospital, China National Clinical Research Center for Neurological Diseases, Advanced Innovation Center for Human Brain Protection, Capital Medical University, Beijing, China.; 3 Department of Internal Medicine, University of Michigan Medical School, Ann Arbor, Michigan, USA.; 4 Department of Metabolic and Bariatric Surgery, The First Affiliated Hospital of Jinan University, Guangzhou, China; State Key Laboratory of Pharmaceutical Biotechnology, The University of Hong Kong, Hong Kong, China.; 5 Department of Hepatobiliary Surgery and; 6 Department of Hematology, The First Affiliated Hospital of Xi’an Jiaotong University, Xi’an, Shaanxi, China.; 7 Spring Biological Technology Development Co., Ltd, Fangchenggang, Guangxi, China.; 8 Guangxi University of Chinese Medicine, Nanning, China.; 9 Department of Endocrinology and Metabolism, Fudan Institute of Metabolic Diseases, Zhongshan Hospital, Fudan University, Shanghai, China.; 10 The Institute of Cardiovascular Sciences, Health Science Center, Peking University, Beijing, China.; 11 Guangdong Provincial Key Laboratory of Large Animal Models for Biomedicine, South China Institute of Large Animal Models for Biomedicine, Wuyi University, Jiangmen, Guangdong, China.; 12 Department of Pathology and Translational Pathobiology, Louisiana State University Health Sciences Center-Shreveport, Shreveport, Louisiana, USA.; 13 State Key Laboratory of Pharmaceutical Biotechnology, Department of Medicine, Guangdong-Hong Kong Joint Laboratory for Metabolic Medicine, The University of Hong Kong, Hong Kong, China.

**Keywords:** Gastroenterology, Hepatology, Microbiome, T cells

## Abstract

The global prevalence of metabolic dysfunction–associated steatohepatitis (MASH) is rising, driven by a complex interplay of metabolic disturbances, inflammation, and fibrosis, yet effective treatment options remain limited. This study examined the relationships among intestinal microbial dysbiosis, ammonia production, and hepatic CD8^+^ T cell activity in MASH, then assessed the therapeutic potential of DT-109, a glycine-based tripeptide. We investigated the gut/liver axis across human cohorts and both nonhuman primate and mouse MASH models. Multiomics approaches were used to characterize ileal microbiota, ammonia levels, and hepatic immune and metabolic pathways. Causality was verified through microbiota transplantation, *C*. *perfringens* *NirA*-knockout mutants, and functional validation in vitro and in vivo. The efficacy of DT-109 was evaluated in nonhuman primates and mice. Our results revealed a significant increase in the ammonia-producing gut bacterium *C*. *perfringens*, which led to elevated intestinal ammonia and disruption of the intestinal barrier in MASH. Elevated ammonia levels triggered FosB-mediated upregulation of *CCL5* in CD8^+^ T cells, which in turn drove T cell cytotoxicity in the liver. Notably, DT-109 effectively lowered *C*. *perfringens* abundance, reduced intestinal ammonia, restored intestinal barrier integrity, and alleviated CD8^+^ T cell dysregulation in MASH. These results identify a distinct mechanism in which gut-derived ammonia drives CD8^+^ T cell–mediated MASH and demonstrate that DT-109 effectively targets this axis by inhibiting *C*. *perfringens* and reducing ammonia, ultimately ameliorating MASH.

## Abstract 2

In MASH, an ammonia-producing gut bacterium increases ammonia, weakens the gut barrier, and overactivates liver immune cells; DT-109 lowers ammonia and improves disease.

## Graphical abstract

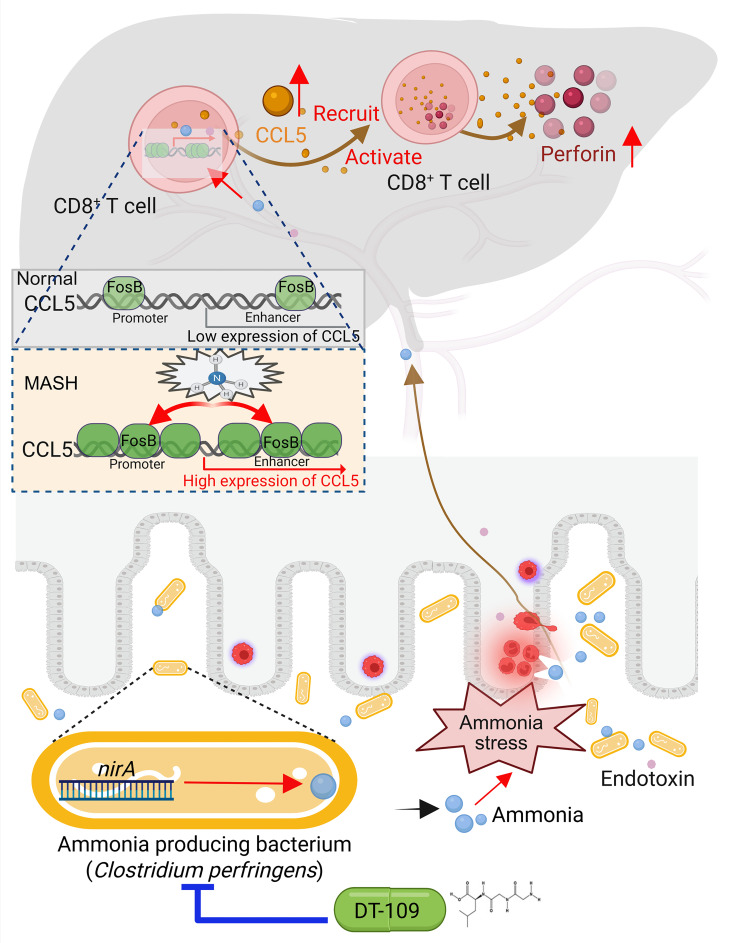

## Introduction

Metabolic dysfunction–associated steatotic liver disease (MASLD) affects nearly one-third of the global population, posing a substantial public health and economic burden (1). About 25% of patients with MASLD progress to metabolic dysfunction–associated steatohepatitis (MASH), which can lead to irreversible cirrhosis and hepatocellular carcinoma (HCC) if untreated (2). The complex pathogenesis of MASH, characterized by metabolic disturbances, chronic inflammation, fibrosis, and carcinogenesis, presents substantial research challenges and underscores the need for targeted therapeutic interventions (3). Recent studies have highlighted the critical role of the CD8^+^ cytotoxic T cell population in MASH progression (4). Auto-aggressive CD8^+^ T cells in the liver contribute to the development of HCC in patients with MASH (5). Paradoxically, although CD8^+^ T cells are typically key mediators of antitumor immunity, in MASH, they can become dysregulated and contribute to tumor-promoting inflammation, tissue damage, and immune evasion by cancer cells (6, 7). Furthermore, MASH impairs CD8^+^ T antitumor surveillance in patients with HCC treated with immune checkpoint inhibitors, specifically via programmed cell death 1/programmed cell death ligand 1 (PD-1/PD-L1) (6). Therefore, developing treatments for MASH by targeting CD8^+^ T cells is of considerable clinical importance.

The gut microbiota plays a critical role in the progression of MASLD by modulating host immune responses (3). Disruption of gut microbiome homeostasis, particularly by pathogenic bacteria, compromises the intestinal barrier, enabling the translocation of endotoxins and other harmful substances to the liver, which triggers inflammation to promote MASLD (8). Furthermore, metabolites produced by gut bacteria, such as secondary bile acids, contribute to MASLD progression (9). Ammonia is a metabolic waste product that microbes use for amino acid synthesis or generate through the degradation of nitrogenous compounds (10, 11). Previous research has primarily focused on hepatic urea cycle disorders leading to ammonia accumulation (12, 13), and the endogenously produced ammonia has been shown to contribute to MASH (12, 14). Mechanistically, hepatic ammonia accumulation drives excessive glutaminolysis, which disrupts metabolic and inflammatory dysregulation, thereby accelerating disease progression (15, 16). However, the contribution of gut microbiota to ammonia production and its potential role in the pathogenesis of MASH remain incompletely understood.

Our prior studies demonstrate that DT-109, a glycine-based tripeptide (Gly-Gly-Leu), ameliorates steatohepatitis and fibrosis by enhancing de novo glutathione biosynthesis, accelerating fatty acid degradation, and reshaping microbial bile acid metabolism (17, 18). Beyond its hepatic benefits, DT-109 also reduces aortic and coronary atherosclerosis by dampening vascular inflammation, blocking the osteogenic phenotypic transition of smooth muscle cells, and reducing oxidative stress (19, 20). In multiple mouse models of type 2 diabetes, DT-109 improves glucose tolerance and lowers blood glucose (21). Building on the promising effects of DT-109 in metabolic disorders, we further investigated whether DT-109 also mitigates MASH by targeting gut-derived ammonia and the associated dysregulation of hepatic CD8^+^ T cells. In this study, we employed multiomics analyses to identify key pathogenic drivers linking the gut and liver in MASH. Our initial analyses revealed a substantial association between CD8^+^ T cell–dependent hepatocyte cytotoxicity and *Clostridium perfringens* (*C*. *perfringens*), an ammonia-producing bacterium (22), which is commonly found in animal intestines and known to cause diseases such as diarrhea and gas gangrene under specific conditions (23). Thus, we tested the central hypothesis that gut-derived ammonia, primarily produced by *C*. *perfringens* overgrowth, is a key pathogenic driver of hepatic CD8^+^ T cell dysregulation and MASH progression. Furthermore, we demonstrated that elevated endotoxin and ammonia levels in MASH promote FosB-mediated upregulation of *CCL5* in CD8^+^ T cells, driving their cytotoxicity in the liver. Notably, DT-109 effectively reduced the abundance of *C*. *perfringens* and intestinal ammonia levels, restored mucosal integrity, and decreased CD8^+^ T cell–dependent hepatotoxicity.

## Results

### Gene expression profiling indicates enhanced CD8^+^ T cell–dependent liver cytotoxicity in MASH.

Twenty monkeys were fed a MASH-inducing diet for 10 months. After confirming the onset of MASH, we randomly divided the monkeys into 2 groups and maintained them on the diet for an additional 5 months: one group received DT-109 (DT-109 group, *n* = 10), and the other received a solvent control (MASH group, *n* = 10). A separate group of 6 monkeys was maintained on a standard diet as normal controls (*n* = 6) (Supplemental Figure 1A; supplemental material available online with this article; https://doi.org/10.1172/JCI200522DS1). At the study endpoint, liver and ileum tissues were collected for analysis. Histological examination revealed healthy liver morphology in the normal control group, whereas the MASH group exhibited marked liver damage, including steatosis, ballooning degeneration, inflammation, and fibrosis, as well as elevated levels of fibrosis marker α–smooth muscle actin and apoptotic cells (Supplemental Figure 1, B–F). In contrast, the DT-109 group exhibited substantial improvement in liver histology (Supplemental Figure 1, B–F).

**Figure 1:**
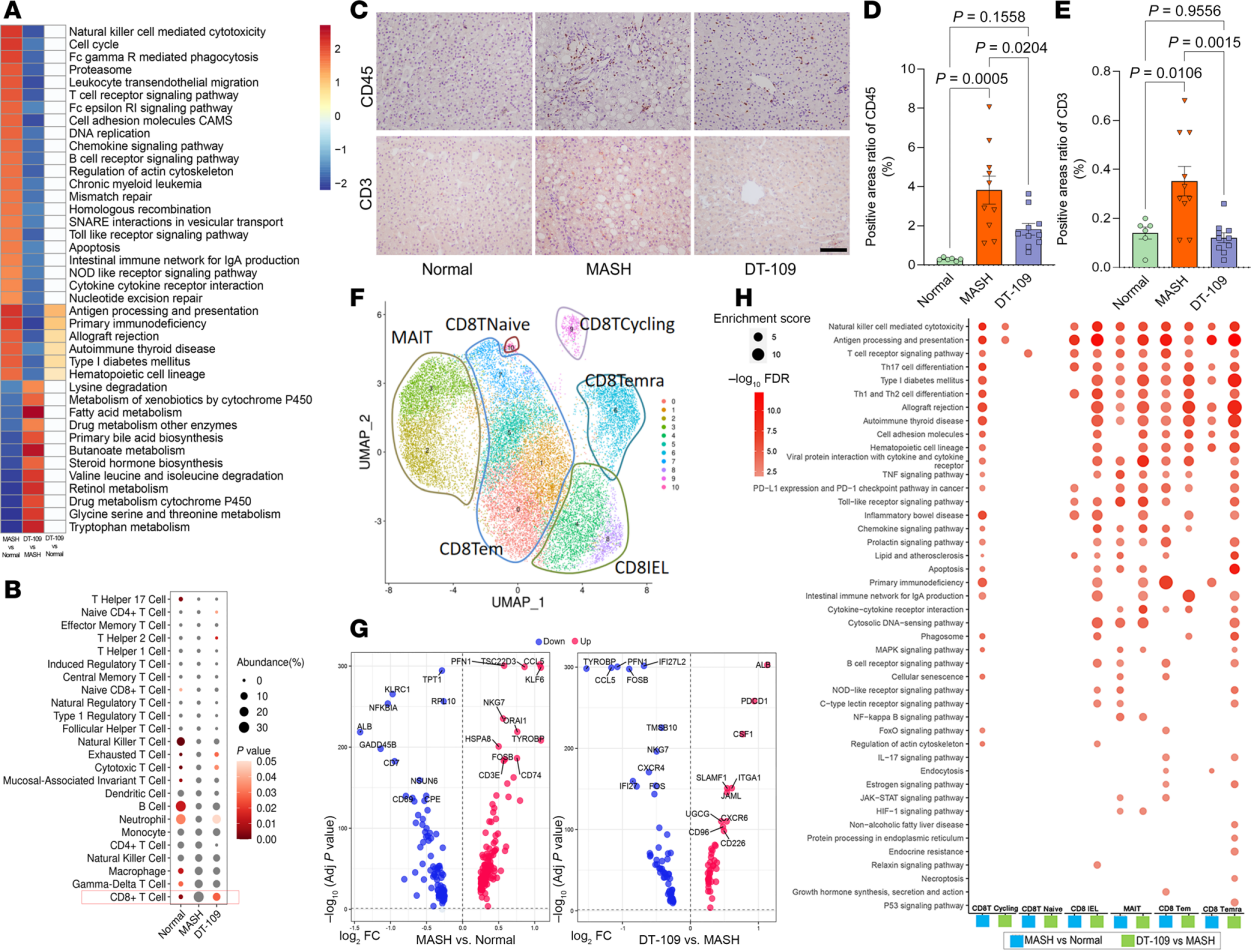
Transcriptomic analysis reveals enhanced CD8^+^ T cell–mediated cytotoxicity in the monkey liver with MASH. (**A**) Heatmap based on gene set enrichment analysis (GSEA) of monkey liver RNA-seq data comparing the DT-109, MASH, and normal groups at endpoint. Significantly upregulated pathways are shown in red and downregulated pathways in blue, and pathways with no significant changes are displayed without color. Scale bars, normalized enrichment score (NES) of the GSEA. (**B**) A bubble plot illustrates the proportion of immune cell abundance in monkey livers, comparing the DT-109, MASH, and normal groups using ImmuCellAI tool. Circle size represents the proportion of cells, and circle color indicates the *P* value. Red circles in the normal and DT-109 groups indicate cell types that differ significantly from the MASH group. Immunohistochemistry (**C**) for CD45 and CD3 in monkey livers from the normal, MASH, and DT-109 groups (scale bars, 100 μm). Ratio of CD45-positive (**D**) and CD3-positive (**E**) areas in monkey livers are compared among the normal (*n* = 6), MASH (*n* = 10), and DT-109 (*n* = 10) groups. Data (**D** and **E**) are presented as means ± SEM. Differences (**D** and **E**) were analyzed using 1-way ANOVA with Dunnett’s post hoc test. (**F**) UMAP visualization showing the clustering of CD8^+^ T cells from scRNA-seq analysis of monkey livers in the normal, MASH, and DT-109 groups (*n* = 3/group), with cells colored by their 6 subtypes. (**G**) Volcano plot depicting DEGs in CD8^+^ T cells for 2 comparisons (MASH vs. normal and DT-109 vs. MASH) based on scRNA-seq. Red dots represent upregulated genes, while blue dots represent downregulated genes in CD8^+^ T cells. *Y* axis denotes −log10 FDR; *x* axis shows log_2_fold-change values. (**H**) Bubble plot of enriched KEGG pathways for DEGs in CD8^+^ T cell subtypes (CD8^+^ Temra cells, CD8^+^ Tem cells, MAIT cells, CD8^+^ IEL cells, CD8^+^ T cycling cells, and CD8^+^ T naive cells) in 2 comparisons (MASH vs. normal, and DT-109 vs. MASH). KEGG pathways have an FDR of less than 0.05. DEGs, differentially expressed genes; scRNA-seq, single-cell RNA-seq; UMAP, uniform manifold approximation and projection. Values above brackets in figures represent *P* values unless indicated as FDR.

Gene set enrichment analysis (GSEA) of liver RNA-seq data revealed a concerted immunologic shift in MASH. Pathways linked to antigen processing and presentation, primary immunodeficiency, and NK cell–mediated cytotoxicity were strongly upregulated in MASH but returned toward baseline with DT-109 treatment. In contrast, amino acid metabolic pathways, such as valine leucine and isoleucine degradation, glycine serine and threonine metabolism, and tryptophan metabolism, were downregulated in MASH monkeys but upregulated in the DT-109 group (Figure 1A). Such a shift was captured by ImmuCellAI analysis, showing a pronounced expansion of hepatic CD8^+^ T cells in MASH that DT-109 largely abolished (Figure 1B). Consistently, CD45 (pan-leukocyte marker) and CD3 (pan–T cell marker) immunohistochemistry (IHC) demonstrated an increase in total leukocyte and T cell area fractions in MASH livers, both of which fell significantly after DT-109 administration (Figure 1, C–E).

Single-cell RNA sequencing (scRNA-seq) of hepatic leukocytes revealed that the ratio of CD4^+^ to total T cells remained consistent across cohorts, whereas CD8^+^ T cells were significantly expanded in MASH (Supplemental Figure 2, A and B). Unsupervised clustering resolved at least 6 major subtypes: naive CD8^+^ T cells, CD8^+^ Tem cells (effector memory T cells) and Temra cells (terminally differentiated effector memory T cells), tissue-resident CD8^+^ MAIT cells (mucosal-associated invariant T cells), IEL cells (intraepithelial lymphocytes), and CD8^+^ T cycling cells (Figure 1F; main gene markers in Supplemental Figure 2, C–G; and all gene markers in Supplemental Table 1). The relative abundance of these subsets was stable among groups, with CD8^+^ Tem cells being the most prevalent and CD8^+^ T naive cells the least (Supplemental Figure 2H). Differential expression analysis identified a MASH-specific upregulation of genes such as *CCL5* and *FOSB* in CD8^+^ T cells (Figure 1G and Supplemental Figure 3, A–E). Notably, DT-109 treatment suppressed both transcripts to near-baseline levels (Figure 1G and Supplemental Figure 3, A–E). Kyoto Encyclopedia of Genes and Genomes (KEGG) pathway analysis based on scRNA-seq also highlighted the enrichment of CD8^+^ T cell–related pathways, including natural killer cell mediated cytotoxicity, antigen processing, and T cell receptor signaling, in MASH versus normal, which was attenuated in DT-109 versus MASH across virtually all non-naive CD8^+^ T cell subsets (Figure 1H).

**Figure 2:**
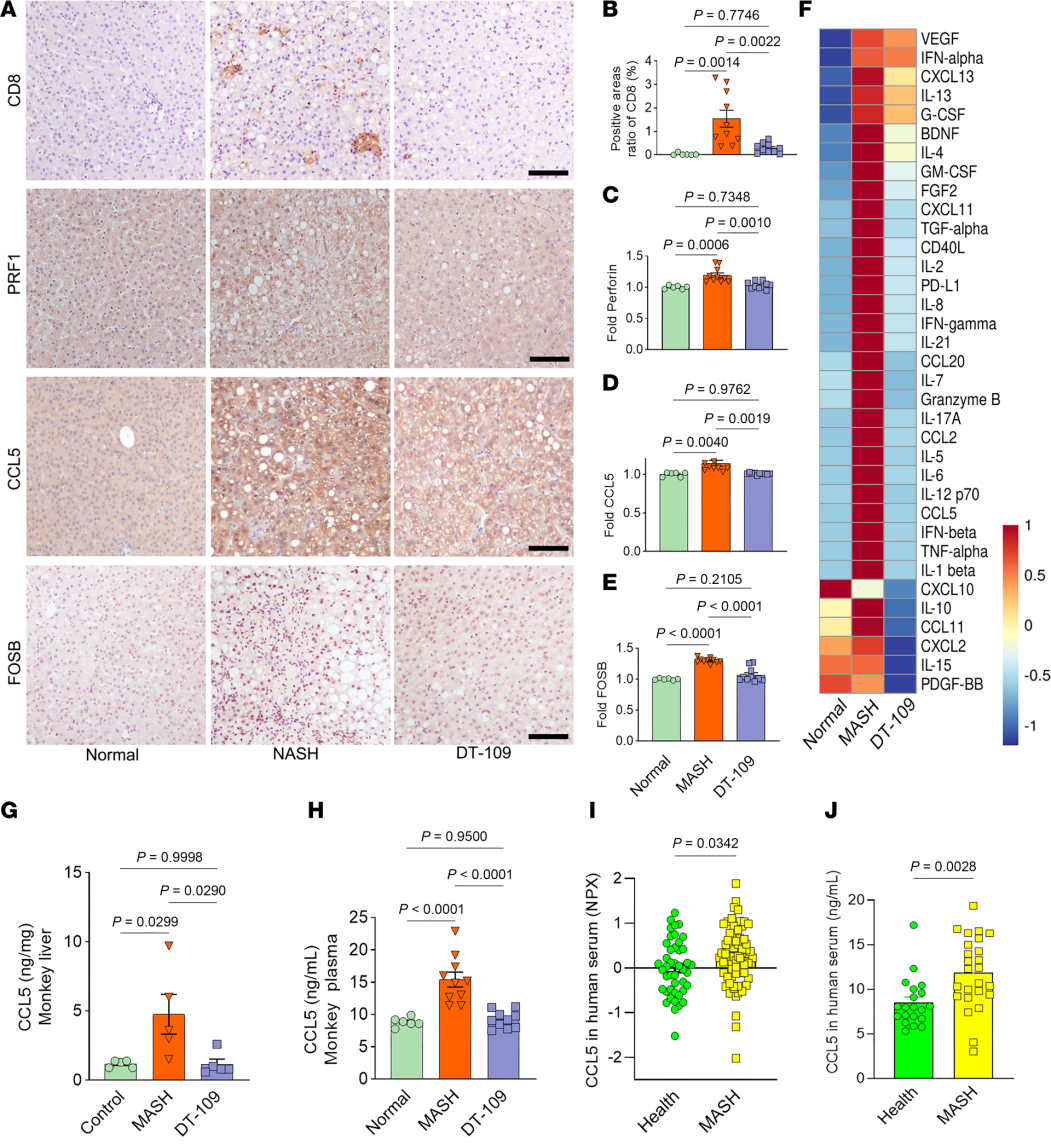
DT-109 effectively restores liver immune homeostasis in monkeys with MASH. (**A**–**E**) Immunohistochemistry (**A**) for CD8, PRF1, CCL5, and FosB in monkey livers from the normal, MASH, and DT-109 groups (scale bars, 100 μm). Quantification of CD8 (**B**), perforin 1 (PRF1) (**C**), CCL5 (**D**), and FosB (**E**) in monkey livers in the normal group (*n* = 6), MASH group (*n* = 10), and DT-109 group (*n* = 10). Abundance of PRF1, CCL5, and FosB was quantified as the fold-change from the normal group based on the mean density. (**F**) Heatmap based on multiple immune factors in monkey livers from the normal, MASH, and DT-109 groups (*n* = 5 per group), generated using the NHP XL Cytokine Premixed assay (catalog FCSTM21, R&D Systems). Scale bars, normalized abundance of immune factors. CCL5 levels in monkey livers (**G**) (*n* = 5/group) and in plasma (**H**) (*n* = 6, normal group; *n* = 10, MASH and DT-109 group) were confirmed using ELISA. CCL5 levels in human serum (cohort 1: 44 healthy individuals and 67 diagnosed with MASH) were analyzed based on Olink assay (**I**). *Y* axis denotes normalized protein expression (NPX). ELISA validation of 45 participants (20 healthy and 25 with MASH) randomly picked from cohort 1 (**J**). Data (**B**–**E** and **G**–**J**) are presented as means ± SEM. Statistical differences were analyzed using Kruskal-Wallis test followed by Dunn’s post hoc test (**B**–**E** and **H**), 1-way ANOVA followed by Dunnett’s post hoc test (**G**), Mann-Whitney *U* test (**I**), or unpaired *t* test (**J**). Values above brackets in figures represent *P* values unless indicated as FDR.

IHC analysis of liver tissue confirmed a significantly higher proportion of CD8^+^ T cells in the MASH group compared with both the normal and DT-109–treated groups (Figure 2, A and B). To delineate the underlying gene-regulatory circuitry, we applied SCENIC (Single-Cell Regulatory Network Inference and Clustering) to the scRNA-seq dataset and ranked transcription factors based on regulon activity (top 50 shown in Supplemental Figure 4A). Notably, changes involving FOSB were particularly noteworthy in our dataset (Supplemental Figure 4A). AUCell analysis further revealed strong regulatory activity of *FOSB* in CD8^+^ T cells (Supplemental Figure 4B). Fifteen predicted target genes of *FOSB* overlapped with differentially expressed genes (DEGs) in both the MASH versus normal and DT-109 versus MASH comparisons, including *CCL5* (Supplemental Figure 4, C and D). *CCL5* is highly expressed in T cells, particularly in CD8^+^ T cells and other cytotoxic T cell subsets, such as NK T cells (Supplemental Figure 5). Additionally, IHC and UMAP analysis revealed that the expression of *CCL5*, *FOSB*, and *PRF1* (the pore-forming effector of cytotoxic cells) was significantly elevated in the MASH group compared with both the normal and DT-109–treated groups (Figure 2, A and C–E, and Supplemental Figure 6, A–C). In parallel, T cell activation marker HLA-DRA was significantly enriched in CD8^+^ T cells in the MASH group compared with the normal and DT-109–treated groups, as demonstrated by UMAP (Supplemental Figure 6, A and D).

Pseudotime analysis of CD8^+^ T cells identified 3 distinct states, with CD8^+^ T naive cells at the proximal end of the cellular trajectory, and CD8^+^ Temra cells and other terminally differentiated cells located at the distal end, consistent with the canonical maturation of CD8^+^ T cells (Supplemental Figure 7, A–D). In the MASH group, both CD8^+^ Temra cells and state 3 (late state) cells were enriched, indicating an accelerated shift toward terminal cytotoxic phenotypes (Supplemental Figure 7, B–D). DT-109 treatment partially reversed this shift, reducing the proportion of cells in the terminal state and CD8^+^ Temra cells (Supplemental Figure 7, C and D). Branch-point analysis further separated the trajectory into 2 divergent fates: cell-fate 1, dominated by MASH fate, and cell-fate 2, characteristic of healthy fates (Supplemental Figure 7E).

To dissect transcriptional kinetics along the pseudotime axis, we clustered the top 1,000 most variable DEGs into 3 temporal modules. These modules describe gene expression dynamics along the 2 inferred trajectories. Cluster 1 genes involving biological processes related to CD8^+^ T cell cytotoxicity, such as antigen processing and presentation, and cytotoxicity, were upregulated in the terminal phase of the MASH state-like trajectory but downregulated in the health state-like trajectory (Supplemental Figure 7E). Cluster 2 genes, primarily involved in maintaining CD8^+^ T cell function, including DNA replication and cellular response, showed stable expression throughout the MASH state-like trajectory but were upregulated in the health state-like trajectory (Supplemental Figure 7E). Cluster 3 genes, involving T cell activation and transcription factor activity, were highly expressed in the early phase of both trajectories but declined over time (Supplemental Figure 7E). However, the expression trends of these genes (cluster 3) in the MASH state-like trajectory did not drop to the same level as in the health state-like trajectory (Supplemental Figure 7E). The gene dynamics suggest that cluster 3 genes respond first, whereas cluster 1 genes act as the ultimate effectors. These findings indicate that CD8^+^ T cells in MASH exhibit increased cytotoxicity and altered immune responses, potentially driving disease progression. This model offers valuable insights into the immune landscape of MASH liver and highlights potential therapeutic targets, such as *FOSB* and *CCL5*, which could play key roles in regulating CD8^+^ T cell function in MASH progression.

### DT-109 ameliorates immune dysregulation in MASH liver.

To further characterize systemic immune dysregulation in MASH and DT-109’s effects, we quantified 35 cytokines, chemokines, and costimulatory molecules in hepatic extracts from normal, MASH, and DT-109–treated monkeys. Our findings revealed marked alterations in the hepatic immune microenvironment of MASH monkeys, with DT-109 treatment effectively reversing many of these changes (Figure 2F and Supplemental Table 2). In the MASH group, 60% (21 out of 35) of the immune-related factors examined were substantially elevated compared with the normal group (Figure 2F). Notably, DT-109 treatment reduced levels of approximately 76% (16 out of 21) of these elevated factors (Figure 2F). Protein levels of key checkpoints and pro-inflammatory mediators, including CD40L, PD-L1, and TNF-α, were significantly increased in MASH and reduced by DT-109 treatment (Figure 2F). Similarly, chemokines crucial for immune cell migration and activity (CCL5, CXCL11, and CXCL13) were elevated in MASH. DT-109 treatment significantly reduced levels of CCL5 and CXCL11. Interferons (IFN-α, IFN-γ) and growth factors (TGF-α, GM-CSF, G-CSF) were also elevated in MASH, with DT-109 reducing IFN-γ, TGF-α, and GM-CSF levels (Figure 2F). Multiple interleukins, key signaling molecules in T cell survival and differentiation, were markedly elevated in MASH. DT-109 treatment effectively reduced levels of several interleukins, including IL-2, IL-5, IL-7, IL-8, IL-12p70, IL-17A, and IL-21 (Figure 2F).

Among the 35 immune analytes, CCL5 exhibited the most pronounced differences in terms of absolute abundance, MASH-to-normal fold-changes, and statistical significance (Figure 2F and Supplemental Table 2). The above hepatic CCL5 was initially screened in 5 randomly selected samples per group using Luminex assay (Figure 2F), followed by ELISA validation (Figure 2G), while plasma CCL5 was validated with ELISA in all available samples (Figure 2H). Moreover, serum CCL5 levels were significantly higher in patients with MASH compared with healthy individuals in a population cohort (Figure 2, I and J). These results demonstrate that MASH is associated with a pro-inflammatory hepatic immune environment, marked by upregulation of various immune factors. DT-109 treatment effectively reverses many of these alterations, potentially reducing inflammation in the MASH liver. The consistent cross-species elevation of CCL5 in monkey samples and human cohorts suggests its potential as a candidate biomarker for MASH.

### DT-109 restores intestinal barrier integrity in MASH monkeys.

**Figure 3:**
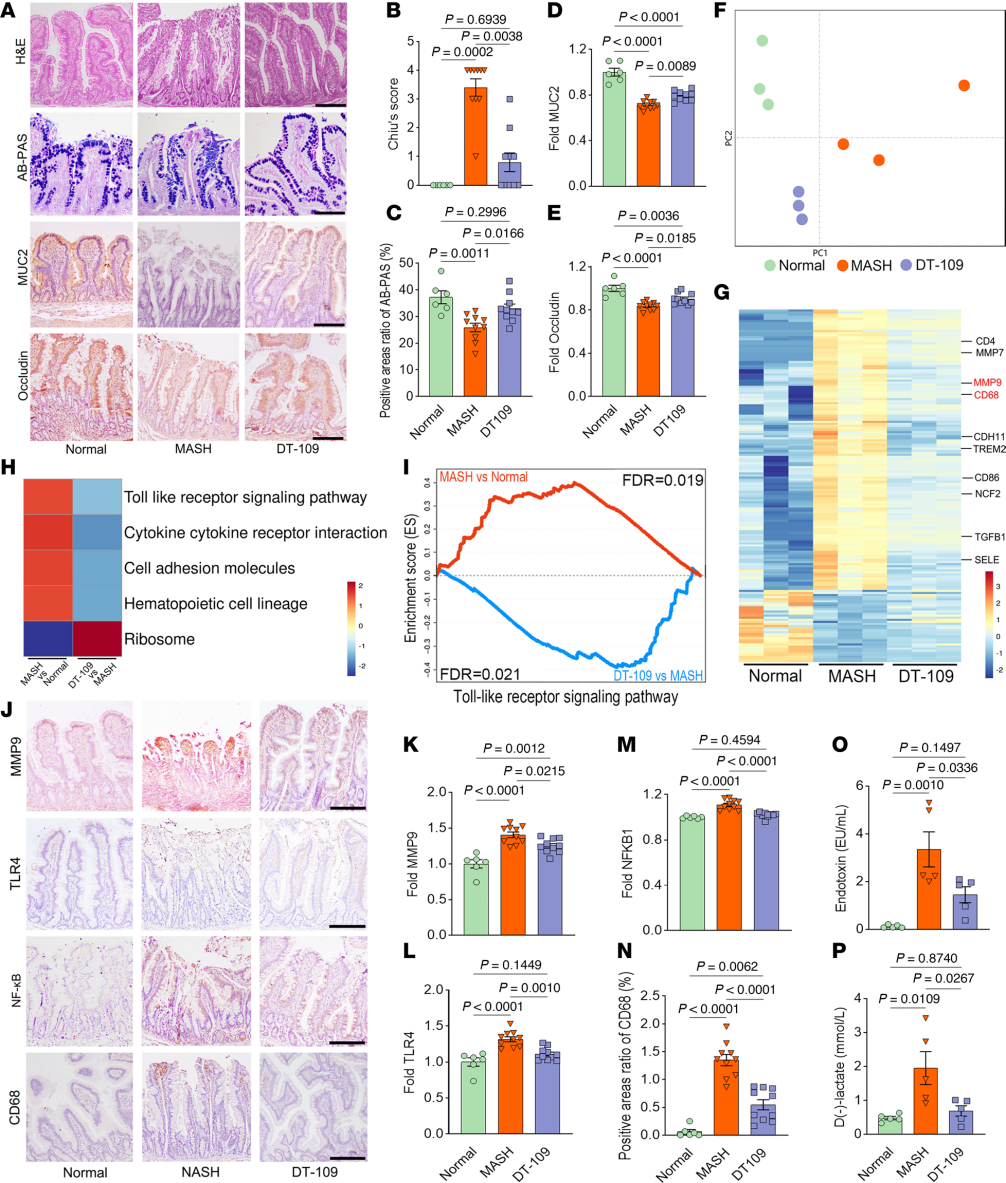
DT-109 alleviates intestinal barrier damage in monkeys with MASH. (**A**) Hematoxylin and eosin (H&E) staining, Alcian blue–periodic acid–Schiff (AB-PAS) staining, and immunohistochemistry for mucin 2 (MUC2) and occludin in monkey ileum sections for the normal, MASH, and DT-109 groups (scale bars, 100 μm). Chiu’s score (**B**), the ratio of AB-PAS–positive areas (**C**), MUC2 mean density (**D**), and occludin mean density (**E**) for the normal (*n* = 6), MASH (*n* = 10), and DT-109 (*n* = 10) groups. (**F**) Principal component analysis (PCA) of RNA-sequencing data of monkey ileum tissues for the 3 groups (*n* = 3/group). (**G**) DEGs for the 3 groups on a heatmap. MMP9 and CD68 (in red), validated by immunohistochemistry, are shown in panel **J**. (**H**) Heatmap based on GSEA among the 3 groups. The scale bars represent the normalized enrichment score (NES). (**I**) GSEA plot of Toll-like receptor signaling pathway, upregulated in MASH and downregulated in DT-109; FDR < 0.05. Immunohistochemistry (**J**) and quantification of MMP9 (**K**), TLR4 (**L**), NFKB1 (**M**), and CD68 (**N**) in the normal (*n* = 6), MASH (*n* = 10), and DT-109 (*n* = 10) groups (scale bars, 100 μm). Plasma endotoxin (**O**) and d-lactate (**P**) levels in normal (*n* = 6), MASH (*n* = 10), and DT-109 (*n* = 10). All points are shown (**B**–**E**, **K**–**P**). Data are presented as means ± SEM. Statistical differences were assessed by the Kruskal-Wallis test followed by Dunn’s post hoc test (**B**, **M**, **N**, and **P**) or by 1-way ANOVA followed by Dunnett’s post hoc test (**C**–**E**, **K**, **L**, and **O**). Values above brackets in figures represent *P* values unless indicated as FDR.

Examination of the ileum revealed severe mucosal damage and compromised barrier integrity in the MASH group. Chiu’s score, a measure of mucosal damage, was significantly higher in the MASH group compared with both the normal control and DT-109 groups (Figure 3, A and B). Additionally, the MASH group showed a markedly lower ratio of Alcian blue–periodic acid–Schiff–positive (AB-PAS^+^) areas in the ileum, indicating reduced mucosal secretion, while the DT-109 group displayed a higher ratio compared with the MASH group (Figure 3, A and C). IHC analysis revealed that levels of MUC2 and occludin, key markers of intestinal mucosal integrity, were significantly reduced in the MASH group compared with normal controls, and the DT-109 group showed significantly higher levels of these markers compared with the MASH group (Figure 3, A, D, and E), which were confirmed by Western blot of MUC2 and occludin (Supplemental Figure 8A). These findings demonstrate that while MASH manifests as substantial liver and intestinal damage, DT-109 treatment significantly mitigates these effects, underscoring its potential as a therapeutic option for mitigating MASH-related pathology. Notably, no significant changes were observed in ileal CD8^+^ T cells among normal, MASH, and DT-109 groups in nonhuman primates (Supplemental Figure 8, B and C). Furthermore, DT-109 treatment did not significantly alter plasma GLP-1 levels in nonhuman primates, indicating its therapeutic benefits are independent of ileal CD8^+^ T cells or are unlikely to be mediated primarily via GLP-1–dependent pathways (Supplemental Figure 8D).

To evaluate changes in intestinal gene expression, bulk RNA-seq was conducted on ileal tissues from the 3 groups. Principal component analysis (PCA) revealed a distinct separation in ileum transcriptome profiles between MASH group monkeys and normal group monkeys, while the DT-109 group occupied an intermediate position (Figure 3F). Compared with the normal group, the MASH group exhibited significant alterations in gene expression, including marked upregulation of immune response genes (*CD68*, *CD86*, *CD4*, *TREM2*) and genes associated with tissue damage (*MMP9*, *MMP7*) (Figure 3G). DT-109 treatment significantly reduced the expression levels of these inflammatory and damage-related marker genes (Figure 3G). GSEA further demonstrated that, compared with the MASH group, immune-inflammatory pathways, such as the Toll-like receptor (TLR) signaling pathway, cytokine-cytokine receptor interactions, and cell adhesion molecules, were upregulated. DT-109 treatment restored these pathways to near-normal levels (Figure 3, H and I). Validation of key markers, including MMP9 and those in the TLR signaling pathway (TLR4, NFKB1, CD68), through IHC confirmed the RNA-seq findings (Figure 3, J–N). Plasma levels of endotoxins (Figure 3O) and d-lactate (Figure 3P) were significantly elevated in monkeys with MASH compared with both normal and DT-109–treated monkeys. Collectively, these findings demonstrate that the intestinal mucosal barrier is compromised in monkeys with MASH, and DT-109 treatment effectively mitigates this damage.

### DT-109 modulates gut microbiota by inhibiting C. perfringens and reducing ammonia levels in MASH.

**Figure 4:**
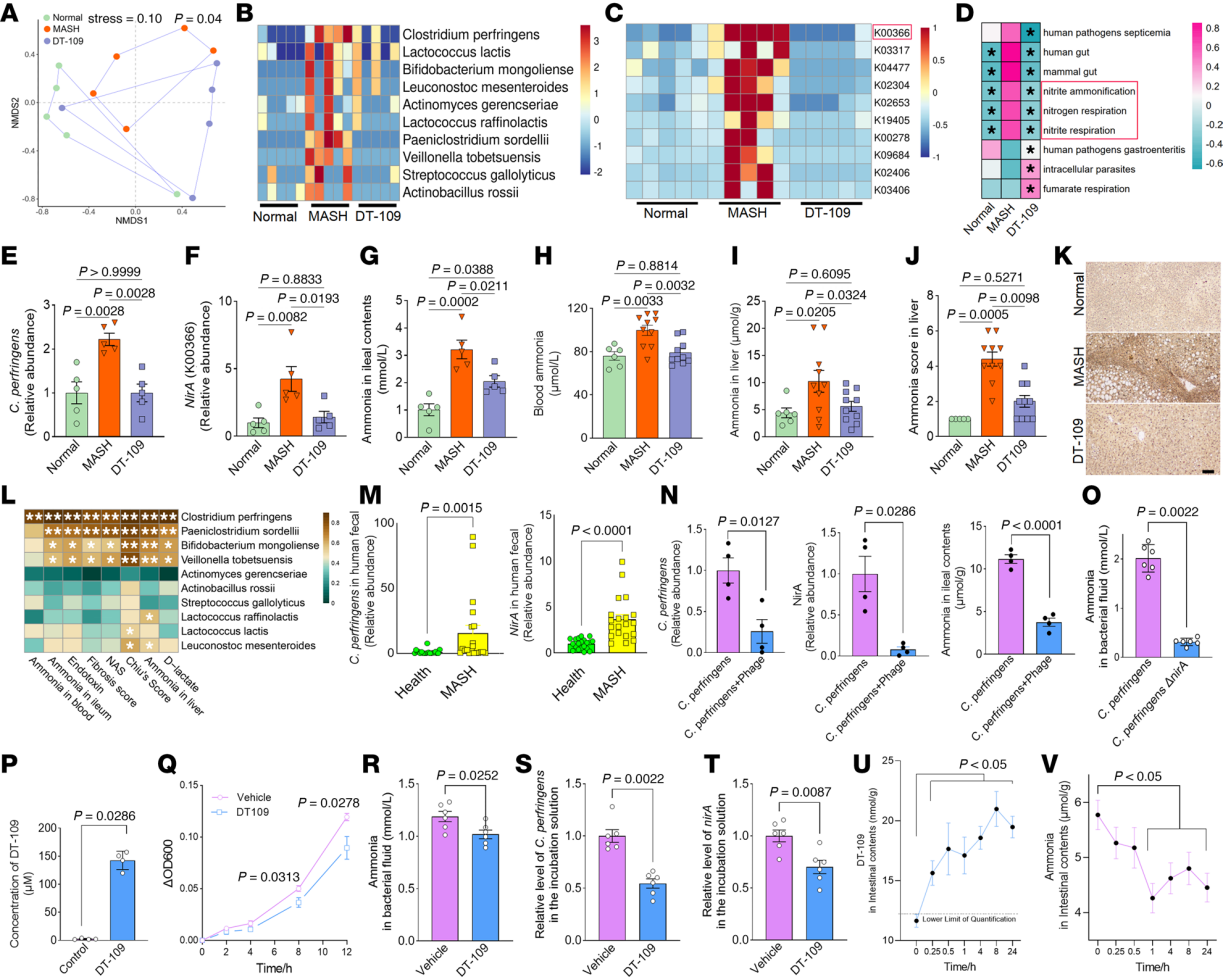
Elevated ammonia-producing gut bacteria in monkeys with MASH. (**A**) Nonmetric multidimensional scaling (NMDS) of metagenome among the 3 groups. (**B**) Heatmap of top 10 differentially expressed bacteria at species level among the 3 groups. Scale bars, normalized species level. (**C**) Heatmap of top 10 differentially expressed macrogenomic genes among the 3 groups. K00366, referred to as *NirA*, as indicated by a red box. Scale bars indicate normalized abundance. (**D**) Comparison of different enriched FAPROTAX (Functional Annotation of Prokaryotic Taxa) functions. Scale bars, normalized functional abundance (*FDR < 0.05) compared with MASH group. The abundance of *C*. *perfringens* (**E**) and *NirA* (**F**). Ammonia levels in ileum contents (**G**), plasma (**H**), and liver (**I**). Hepatic ammonia score (**J**) by Nessler staining. Nessler’s staining (**K**) (scale bars, 100 μm). (**L**) Correlations between significantly altered microbiota, MASH-related or intestinal barrier damage parameters, and ammonia concentrations. (**M**) *C*. *perfringens* and *NirA* in 40 participants from cohort 1. (**N**) *C*. *perfringens*, *NirA*, and ammonia in ileum contents of mice (*n* = 4/group). (**O**) Ammonia between wild-type *C*. *perfringens* and *C*. *perfringens* Δ*NirA* mutant strain (*n* = 6/group). (**P**) DT-109 within *C*. *perfringens* and the control solution was detected (*n* = 4/group). *C*. *perfringens* over 0–12 hours (**Q**) and ammonia at 12 hours (**R**) with or without DT-109 (*n* = 6/group). *C*. *perfringens* (**S**) and *NirA* (**T**) with or without DT-109 (*n* = 6/group). DT-109 (**U**) and ammonia (**V**) in mouse intestinal contents after oral gavage of DT-109 (*n* = 5/time point). Significantly different species (**B**), genes (**C**), and functional profiles (**D**) were analyzed by DESeq2. Data (**E**–**J**, **M**–**V**) are presented as means ± SEM. Statistical differences were analyzed using Kruskal-Wallis test followed by Dunn’s post hoc test (**E** and **J**), 1-way ANOVA followed by Dunnett’s post hoc test (**F**–**I**), unpaired *t* test (**O**, **Q**–**V**), or Mann-Whitney *U* test (**M**, **N**, and **P**). (**L**) *FDR < 0.05; **FDR < 0.01. Values above brackets in figures represent *P* values unless indicated as FDR.

To investigate the mechanisms underlying intestinal mucosal damage in MASH, we performed metagenomic sequencing of ileal microbiota. Nonmetric multidimensional scaling (NMDS) analysis revealed a clear separation between the MASH and normal groups, with the DT-109–treated group positioned intermediately between them (Figure 4A). Analysis of the top 10 bacterial taxa, from phylum to species levels, identified significant differences between the MASH and normal groups, including changes in Actinomycetales (order), Clostridiaceae and Aerococcaceae (families), *Clostridium* and *Abiotrophia* (genera), and *C*. *perfringens* (species) (Supplemental Figure 9, A–F). In addition, significant differences were observed in Enterococcaceae (family) and *C*. *perfringens* (species) when comparing the DT-109 group and the MASH group (Supplemental Figure 9, A–F). Further differential comparison of the microbiota at the species level among the 3 groups revealed that *C*. *perfringens*, *Lactococcus lactis*, *Paeniclostridium sordellii*, and *Streptococcus gallolyticus* were significantly more abundant in the MASH group compared with both normal and DT-109 groups (Figure 4B). Among these, *C*. *perfringens* emerged as the most significantly altered bacterium in the intestinal samples from the MASH monkeys (Supplemental Figure 9G). Differential analysis of macrogenomic genes’ abundance in the intestinal microbiota identified the top 10 differentially abundant genes among the 3 groups: *K00366*, *K03317*, *K04477*, *K02304*, *K02653*, *K19405*, *K00278*, *K09684*, *K02406*, and *K03406* (Figure 4C). Predictive functional analysis using FAPROTAX (Functional Annotation of Prokaryotic Taxa) revealed significant differences in nitrogen respiration, nitrite respiration, and nitrite ammonification between the MASH and normal groups, as well as between the DT-109 and MASH groups (Figure 4D). Notably, K00366, known as *NirA*, plays a crucial role in ammonia production by converting nitrate to ammonia (24). Validation of the *C*. *perfringens* and *NirA* genes via PCR confirmed the metagenomic findings (Figure 4, E and F). *C*. *perfringens*, an important producer of ammonia (22), exhibited significant changes in abundance, suggesting possible dysregulation of ammonia metabolism in the intestines of MASH monkeys. Ammonia quantification revealed that ammonia levels in the ileal contents of the MASH group were significantly higher than those in the normal group, and DT-109 treatment significantly reduced the ammonia levels (Figure 4G). Changes in ileal ammonia levels were also accompanied by corresponding changes in plasma and liver ammonia levels (Figure 4, H–K). Previous studies have reported that changes in circulating ammonia are primarily influenced by the liver (12). Our results suggest that ammonia derived from the gut microbiota may also influence systemic levels. Correlation analysis of differential microbiota and ileal ammonia, plasma ammonia, liver ammonia, plasma endotoxins and d-lactate, NAFLD activity score (NAS), liver fibrosis score, and intestinal Chiu’s score revealed a significant association between *C*. *perfringens* and these parameters (Figure 4L). Also, qPCR analysis of human fecal samples showed significantly increased *C*. *perfringens* and *NirA* abundance in patients with MASH compared with healthy controls (Figure 4M).

To further validate the impact of *C*. *perfringens* and DT-109 on ammonia levels, we performed a phage interference experiment in mice. We isolated a bacteriophage that specifically lyses *C*. *perfringens*. In the intervention study, one group of mice received *C*. *perfringens* transplantation alone (*C*. *perfringens* group), whereas a second group received the same transplantation followed by phage gavage (*C*. *perfringens*+Phage group). Analysis of intestinal contents after 2 weeks revealed significantly lower levels of *C*. *perfringens*, *NirA*, and intestinal ammonia in the *C*. *perfringens*+Phage group compared with the *C*. *perfringens* group (Figure 4N). A *NirA*-knockout mutant strain of *C*. *perfringens* (*C*. *perfringens* Δ*NirA*) was constructed and functionally characterized. In vitro assays definitively confirmed an impairment in ammonia production capability in the Δ*NirA* mutant (Figure 4O).

To investigate the direct effects of DT-109 on *C*. *perfringens* in an in vitro setting, we examined whether DT-109 could penetrate the bacterial cell wall. *C*. *perfringens* was coincubated with DT-109 for 1 hour, and the entry of DT-109 into the bacteria was monitored. We found that, compared with the control group, the DT-109–supplemented group exhibited significant entry and enrichment of DT-109 within the bacteria, indicating that DT-109 can successfully enter *C*. *perfringens* (Figure 4P and Supplemental Figure 10A). Next, we assessed the impact of DT-109 on bacterial growth and ammonia production in vitro. In vitro culture of *C*. *perfringens* treated with DT-109 demonstrated that DT-109 significantly reduced bacterial proliferation and ammonia production in the medium (Figure 4, Q and R). Furthermore, to model the interaction in a more complex gut-like environment, we conducted in vitro coculture experiments. *C*. *perfringens* was coincubated with intestinal contents in the presence or absence of DT-109. DT-109 treatment significantly reduced the abundance of *C*. *perfringens* and *NirA* in the incubation solution (Figure 4, S and T). To determine whether DT-109 could effectively reduce intestinal ammonia levels in vivo and correlate its presence with this effect, after oral gavage administration of DT-109 to mice, DT-109 and ammonia levels in intestinal contents were measured at 0, 0.25, 0.5, 1, 4, 8, and 24 hours. Results showed that intestinal DT-109 levels were significantly elevated from 0.25 to 24 hours postadministration (Figure 4U), whereas ammonia concentrations were significantly reduced from 1 to 24 hours (Figure 4V), providing direct in vivo evidence for DT-109’s efficacy in suppressing intestinal ammonia. In vitro, DT-109 did not significantly affect *FosB* and *Ccl5* upregulation in ammonia-treated mouse CD8^+^ T cells (Supplemental Figure 10, B and C), supporting an indirect mechanism via ammonia reduction rather than DT-109’s direct targeting of CD8^+^ T cells.

### Gut-derived ammonia accumulation causes intestinal mucosal damage in MASH.

**Figure 5:**
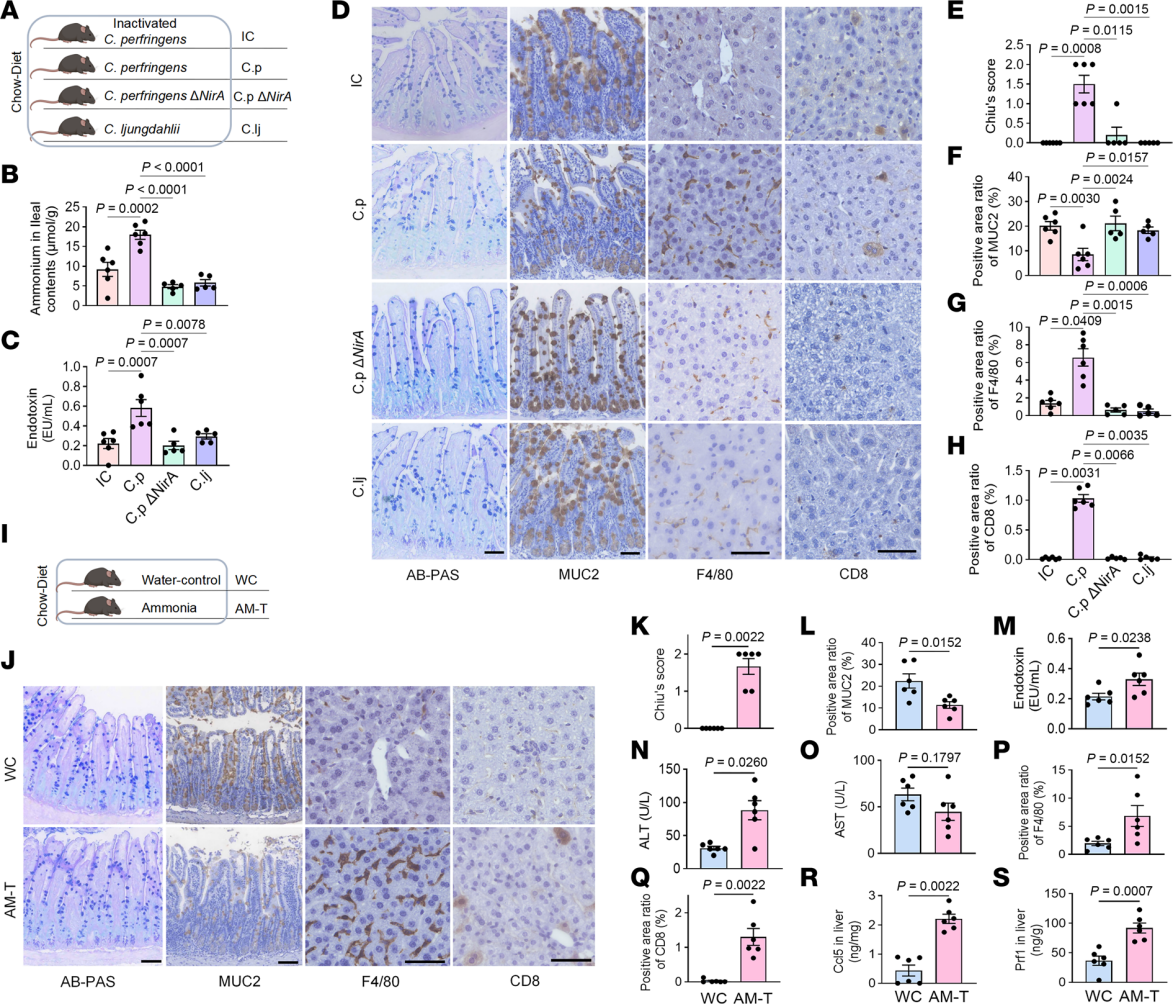
Ammonia produced by *C*. *perfringens* induces gut barrier disruption and hepatic inflammation. (**A**–**H**) Bacterial colonization experiment. (**A**) Schematic of experimental design: mice (C57BL/6) colonized with heat-inactivated bacteria (IC group), wild-type *C*. *perfringens* (C.p group), *C*. *perfringens* Δ*NirA* (C.p Δ*NirA* group), or *C*. *ljungdahlii* (*C.lj* group) for 8 weeks. (**B**) Ileal content ammonia concentrations. (**C**) Plasma endotoxin levels. (**D**) Histological and immunohistochemical sections in the ileum and liver (scale bars, 100 μm). Quantification of ileal mucosal damage by Chiu’s score (**E**) and MUC2 expression (**F**). Hepatic F4/80 macrophages (**G**) and CD8^+^ T cells (**H**). (**I**–**S**) Ammonium chloride treatment experiment. (**I**) Schematic of ammonium chloride treatment: mice (AM-T group) received oral gavage of ammonium chloride for 8 weeks vs. water (vehicle) control (WC group). (**J**) Histological and immunohistochemical sections in the ileum and liver (scale bars, 100 μm). Chiu’s score (**K**) and MUC2 expression (**L**) in the ileum. (**M**) Endotoxin levels in portal vein blood. Plasma ALT (**N**) and AST (**O**) levels. Hepatic F4/80 macrophages (**P**) and CD8^+^ T cells (**Q**). Hepatic *Ccl5* (**R**) and *Prf1* (**S**) concentrations. Data (**B**, **C**, **E**–**H**, **K**–**S**) are presented as means ± SEM. Statistical differences were analyzed using Kruskal-Wallis test followed by Dunn’s post hoc test (**E**, **G**, and **H**), 1-way ANOVA followed by Dunnett’s post hoc test (**B**, **C**, and **F**), unpaired *t* test (**L**, **M**, **O**, **R**, and **S**), or Mann-Whitney *U* test (**K**, **N**, **P**, and **Q**). Values above brackets in figures represent *P* values unless indicated as FDR.

To determine the role of ammonia accumulation by *C*. *perfringens* in MASH, mice (C57BL/6) were transplanted with heat-inactivated bacteria (inactivated control, IC group), wild-type *C*. *perfringens* (*C.p* group), *C*. *perfringens* *ΔNirA* (a *NirA-*knockout mutant strain, *C.p* *ΔNirA* group), or *C*. *ljungdahlii* (a *Clostridium* strain control, *C.lj* group) for 8 weeks (Figure 5A). Ileal content ammonia concentrations (Figure 5B) and plasma endotoxin levels (Figure 5C) were significantly higher in the *C.p* group than in the IC group; both the *C.p* Δ*NirA* group and the *C.lj* group showed significantly lower levels than the *C.p* group (Figure 5, B and C). Histological analysis of the ileum revealed that mice transplanted with *C*. *perfringens* (*C.p* group) displayed significant mucosal damage, as indicated by higher Chiu’s scores (Figure 5, D and E) and lower expression level of MUC2 (Figure 5, D and F) compared with the IC group, whereas the *C.p* Δ*NirA* group and the *C.lj* group did not exhibit significant mucosal damage relative to the *C.p* group (Figure 5, D–F). These mice in the *C.p* group also exhibited an increased hepatic F4/80 macrophage (Figure 5, D and G) and CD8^+^ T cell numbers (Figure 5, D and H) compared with the IC group, while no such increases were observed in either the *C.p* Δ*NirA* group or the *C.lj* group (Figure 5, D, G, and H). C57BL/6 mice fed a MASH diet for 3 months (treatments during final month) were transplanted with *C*. *perfringens* (MASH *C.p*), *C*. *perfringens* Δ*NirA* (MASH *C.p* Δ*NirA*), *C*. *ljungdahlii* (MASH *C.lj*), or an untreated MASH as control group (MASH Ctrl) (Supplemental Figure 11A). The MASH *C.p* group exhibited significantly reduced ileal MUC2 expression and elevated hepatic F4/80 macrophages, CD8^+^ T cell numbers, and alanine aminotransferase (ALT) levels compared with MASH Ctrl, whereas neither the MASH *C.p* Δ*NirA* nor MASH *C.lj* groups showed these alterations (Supplemental Figure 11, B and C).

To further confirm the direct pathogenic role of ammonia in mediating these effects, C57BL/6 mice (AM-T group) were administered ammonium chloride via oral gavage for 8 weeks, while the control group (WC group) received water (Figure 5I). Comparison of ileal histopathology between the 2 groups revealed that mice treated with ammonium chloride exhibited mucosal damage, with significantly higher Chiu’s scores (Figure 5, J and K), lower expression level of MUC2 (Figure 5, J and L), and elevated plasma endotoxin levels (Figure 5M). Ammonia treatment did not significantly alter CD8^+^ cells in the ileum of mice, consistent with the MASH primate findings (Supplemental Figure 12, A and B). Western blot confirmed that ammonia did not upregulate CD8, FosB, or CCL5 in the ileum of mice (Supplemental Figure 12, C and D). Also, CD8^+^ T cell depletion did not mitigate ammonia-induced intestinal barrier damage (Supplemental Figure 12, E–G). Compared with the WC group, the AM-T group mice showed significantly increased plasma ALT levels (Figure 5N), while aspartate aminotransferase (AST) levels showed no significant difference (Figure 5O). Additionally, the AM-T group exhibited significantly increased hepatic numbers of F4/80^+^ macrophages (Figure 5P) and CD8^+^ T cells (Figure 5Q), as well as significantly elevated hepatic concentration of Ccl5 (Figure 5R) and Prf1 (Figure 5S). To elucidate the role of CD8^+^ T cells in ammonia-induced hepatotoxicity, 3 additional independent experiments were conducted. First, groups comprising Ctrl (Balb/c mice, no ammonia), Balb/c+A (Balb/c mice, 1-week ammonia), and Balb/c-nu+A (T cell–deficient mice, 1-week ammonia) were compared (Supplemental Figure 13A). Significantly elevated ALT levels and hepatic Ccl5 and Prf1 concentrations were observed in the Balb/c+A group relative to both the Ctrl and Balb/c-nu+A groups (Supplemental Figure 13A). Second, in C57BL/6 mice, analyses of Non-A (no ammonia), IgG + A (IgG control + ammonia), and CD8-anti + A (CD8^+^ T cell–depleted + ammonia) groups revealed significantly higher ALT, Ccl5, and Prf1 levels in the IgG + A group compared with the Non-A and CD8-anti + A groups, confirming CD8^+^ T cell–dependent ammonia hepatotoxicity (Supplemental Figure 13B). Finally, C57BL/6 mice were fed a MASH diet (high fructose, fat, and cholesterol) for 3 months with interventions administered during the final month. Four ammonia-treated groups were evaluated: IgG, CD8-anti (CD8^+^ T cell–depleted), PBS, and maraviroc (CCL5 inhibitor) (Supplemental Figure 13C). Both the CD8-anti and maraviroc groups exhibited attenuated MASH pathology and reduced ALT levels (Supplemental Figure 13, D and E). Collectively, these findings provide compelling evidence that ammonia directly induces intestinal and hepatic injury in a CD8^+^ T cell– and CCL5-dependent manner, further underscoring the critical role of gut-derived ammonia and immune interactions in driving MASH pathogenesis.

### Ammonia-induced FosB mediates CCL5 upregulation and drives CD8^+^ T cell cytotoxicity in MASH progression.

**Figure 6:**
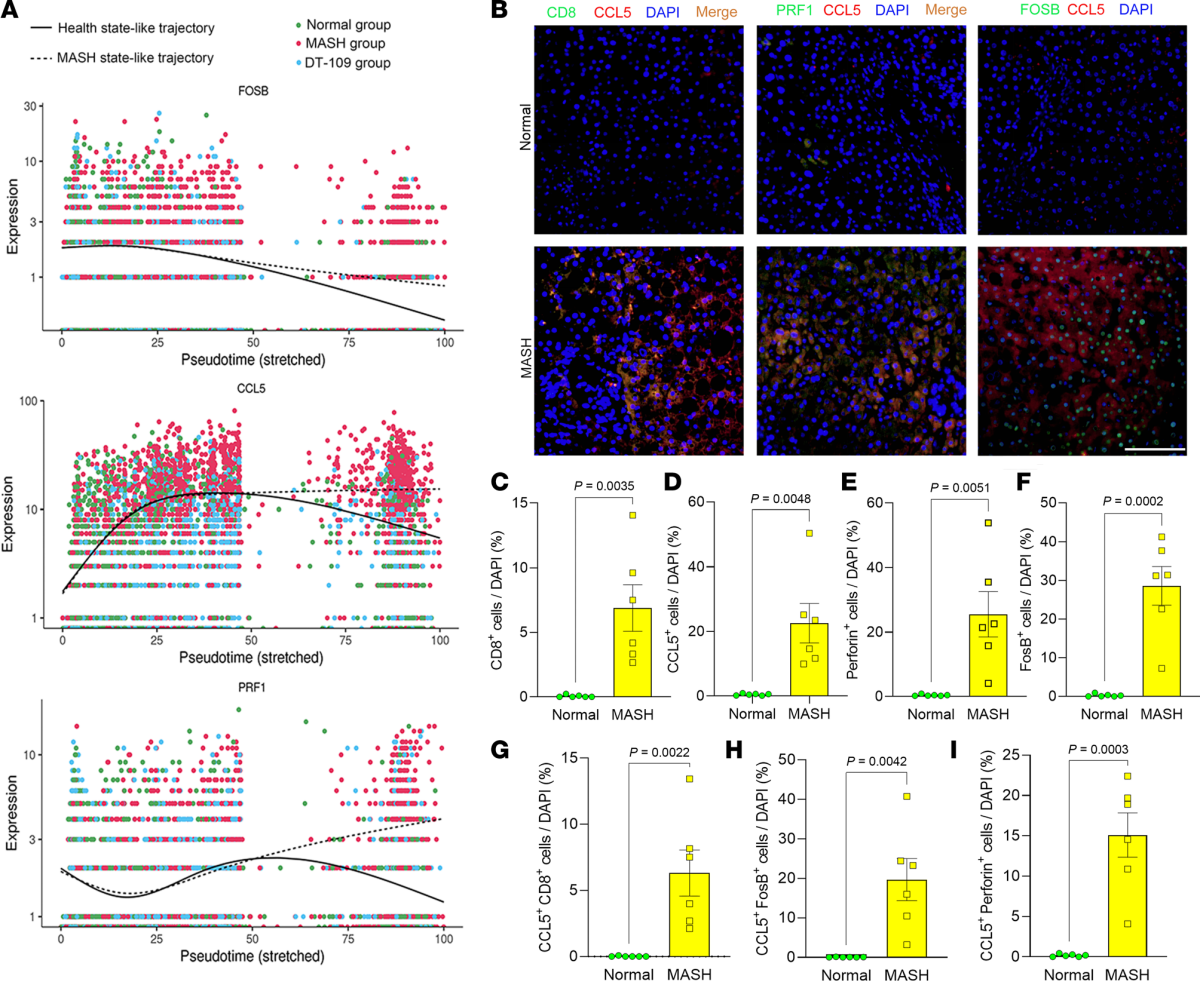
Elevated expression of FOSB, CCL5, and PRF1 in CD8^+^ T cells in MASH livers. (**A**) Expression dynamics of FOSB, CCL5, and PRF1 over the pseudotime trajectories (the health state-like trajectory and the MASH state-like trajectory) of CD8^+^ T cells in monkey livers from the normal, MASH, and DT-109 groups (*n* = 3/group), shown as dot plots representing the expression level. Representative images of immunofluorescence staining (**B**) for CD8, PRF1, CCL5, and FOSB in human livers (cohort 2: 6 healthy individuals and 6 patients diagnosed with MASH) (scale bars, 100 μm). The ratio of CD8-positive (**C**), CCL5-positive (**D**), PRF1-positive (**E**), and FosB-positive (**F**) cells in human livers from cohort 2. The ratios of CD8 & CCL5 double-positive cells (**G**), FosB & CCL5 double-positive cells (**H**), and PRF1 & CCL5 double-positive cells (**I**) are shown in human livers from healthy individuals and patients diagnosed with MASH (cohort 2). Data (**C**–**I**) are presented as means ± SEM. Statistical differences were assessed by the Mann-Whitney *U* test (**C**–**I**). Values above brackets in figures represent *P* values unless indicated as FDR.

In the pseudotime trajectory analysis of CD8^+^ T cells in monkey livers, *FOSB* was identified in cluster 3, whereas *CCL5* and *PRF1* were found in cluster 1 (Supplemental Figure 7E). These dynamic gene expression changes along the trajectories highlight their crucial role in MASH progression. Notably, *FOSB*, *CCL5*, and *PRF1* exhibited marked expression changes (Figure 6A). Consistent with the trajectory analysis, DT-109 treatment ameliorated the dysregulation of these genes. In later stages of the trajectories, the expression levels of these genes were markedly higher in the MASH state-like trajectory compared with the health state-like trajectory (Figure 6A). Immunofluorescence staining of liver tissues from patients with MASH (*n* = 6) and healthy controls (*n* = 6) demonstrated an increased presence of CD8-positive, CCL5-positive, and PRF1-positive cells in MASH patient livers (Figure 6, B–E). FosB-positive cells were also more abundant in MASH patient livers (Figure 6, B and F). CD8-positive cells appeared to preferentially orient toward CCL5, supporting previous findings that CCL5 recruits CD8^+^ T cells (Figure 6, B and G). Additionally, FOSB and PRF1 in MASH livers showed substantial overlap with CCL5-positive cells (Figure 6, B, H, and I).

**Figure 7:**
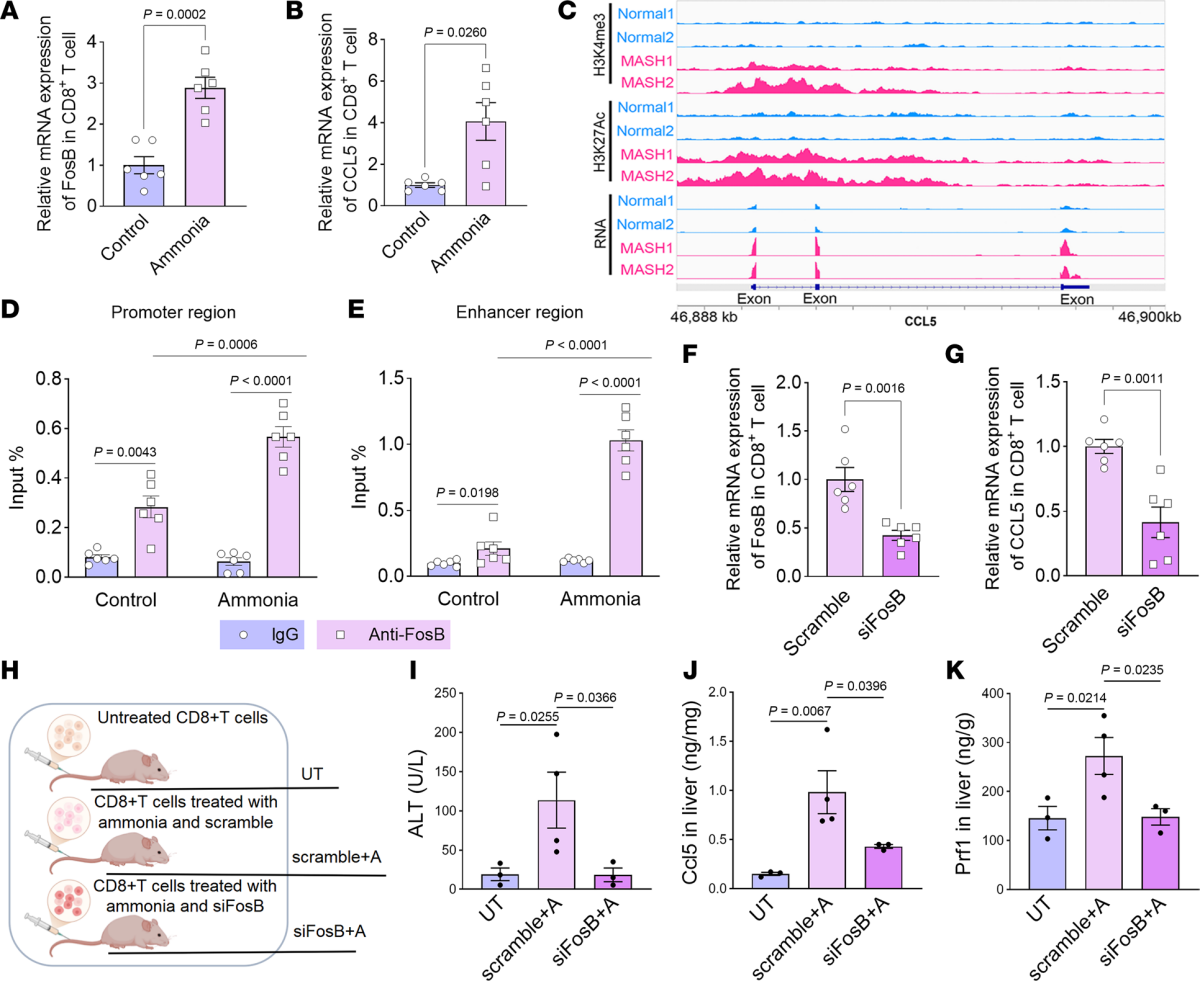
Elevated ammonia triggers FosB-mediated *CCL5* expression in CD8^+^ T cells, driving MASH progression. Relative mRNA expression levels of *FosB* (**A**) and *Ccl5* (**B**) in mouse CD8^+^ T cells in the control and ammonia groups. (**C**) ChIP-seq results of active histone modification markers H3 trimethylated at lysine 4 (H3K4me3) and H3 acetylated at lysine 27 (H3K27ac) and RNA-seq results for CCL5 and its adjacent DNA sequences in monkey livers from the MASH group (marked in red) and normal group (marked in blue) (*n* = 2/group). (**D** and **E**) FosB ChIP-qPCR analyses of CD8^+^ T cells of mice in the control and ammonia groups (*n* = 6/group). FosB binding signals were detected at the promoter region (**D**) and the enhancer region (**E**) of Ccl5. IgG was used as a negative control. mRNA expression of *FosB* (**F**) and *Ccl5* (**G**) in mouse CD8^+^ T cells transfected with control siRNA (siCtrl) or FosB-specific siRNA (siFosB) under ammonia exposure (*n* = 6). (**H**) Schematic of adoptive transfer experiment: CD8^+^ T cells from 3 groups were adoptively transferred into nude mice: untreated controls (UT, without ammonia exposure or siRNA transfection), scramble siRNA–transfected cells, and siFosB-transfected cells. Cells in the latter 2 groups were exposed to ammonia for 24 hours in vitro prior to transfer. (**I**) Plasma ALT levels in recipient nude mice 48 hours after transfer of CD8^+^ T cells. (**J** and **K**) Hepatic Ccl5 and Prf1 in recipient nude mice 48 hours after transfer. Data (**A**, **B**, **D**–**G**, and **I**–**K**) are presented as means ± SEM. Statistical differences were analyzed using Kruskal-Wallis test followed by Dunn’s post hoc test (**I**), 1-way ANOVA followed by Dunnett’s post hoc test (**J** and **K**), Mann-Whitney *U* test (**A**, **B**, **D**, and **E**), or unpaired *t* test (**F** and **G**). Values above brackets in figures represent *P* values unless indicated as FDR.

Given that elevated ammonia serves as a significant environmental stimulus in MASH liver pathology, its effects on CD8^+^ T cell activation and *CCL5* expression were examined. Mouse primary CD8^+^ T cells were isolated, cultured in vitro, and treated with ammonium chloride. Results showed that *Ccl5* and *FosB* expression levels were significantly higher in the ammonia-treated group compared with the control (Figure 7, A and B). ChIP-seq analysis of liver tissues from monkeys in both the MASH and normal groups demonstrated significant enrichment of the histone modifications histone H3K4me3 and H3K27ac at the *CCL5* promoter and enhancer regions in the MASH group (Figure 7C). This suggests increased accessibility of the *CCL5* promoter and enhancer regions in MASH, facilitating transcription factor interactions. In vitro ammonia treatment alone did not significantly alter the enrichment of H3K27ac or H3K4me3 at the *Ccl5* locus in mouse primary CD8^+^ T cells. Instead, we found that palmitic acid (PA), a key mediator of lipotoxicity in MASH, significantly upregulated these activating histone marks at the *Ccl5* promoter (Supplemental Figure 14). This indicates that the observed chromatin remodeling in vivo is likely driven by the local lipotoxic milieu, whereas ammonia may operate through its corresponding *trans*-acting factor. Further ChIP-PCR analysis of in vitro–cultured mouse primary CD8^+^ T cells showed a significant enrichment of *FosB* at the presumptive promoter or enhancer region of *Ccl5*, and the enrichment was more substantial in the ammonia-stimulated group (Figure 7, D and E). Notably, *FosB* knockdown under ammonia stimulation did not affect H3K27ac or H3K4me3 levels at the *Ccl5* promoter, confirming that FosB functions primarily as a *trans*-acting factor binding to regulatory elements (Supplemental Figure 14). This underscores FosB’s crucial role in promoters and enhancers by regulating gene expression, including *CCL5*, which is vital for MASH progression. Therefore, high ammonia levels enhance CD8^+^ T cell cytotoxicity by promoting FosB-mediated transcriptional activation at the *CCL5* promoter and enhancer regions, resulting in increased *CCL5* expression. To confirm the functional role of FosB in this ammonia/CCL5 pathway, FosB-specific siRNA was used to knock down *FosB* expression in mouse CD8^+^ T cells under ammonia-exposed conditions, with both siRNA transfection and ammonia treatment (for 24 hours). This intervention effectively reduced *FosB* mRNA levels (Figure 7F) and significantly downregulated transcription of its key downstream molecule, *Ccl5* (Figure 7G). Following adoptive transfer of the treated CD8^+^ T cells into nude mice (Figure 7H), plasma ALT levels (Figure 7I), as well as hepatic Ccl5 (Figure 7J) and Prf1 (Figure 7K) levels, were significantly reduced in the siFosB group 48 hours after transfer. Together, these data reveal a cooperative model in MASH where lipotoxicity promotes a permissive chromatin state at the *CCL5* locus, while gut-derived ammonia potentiates its expression via FosB, driving CCL5 upregulation.

**Figure 8:**
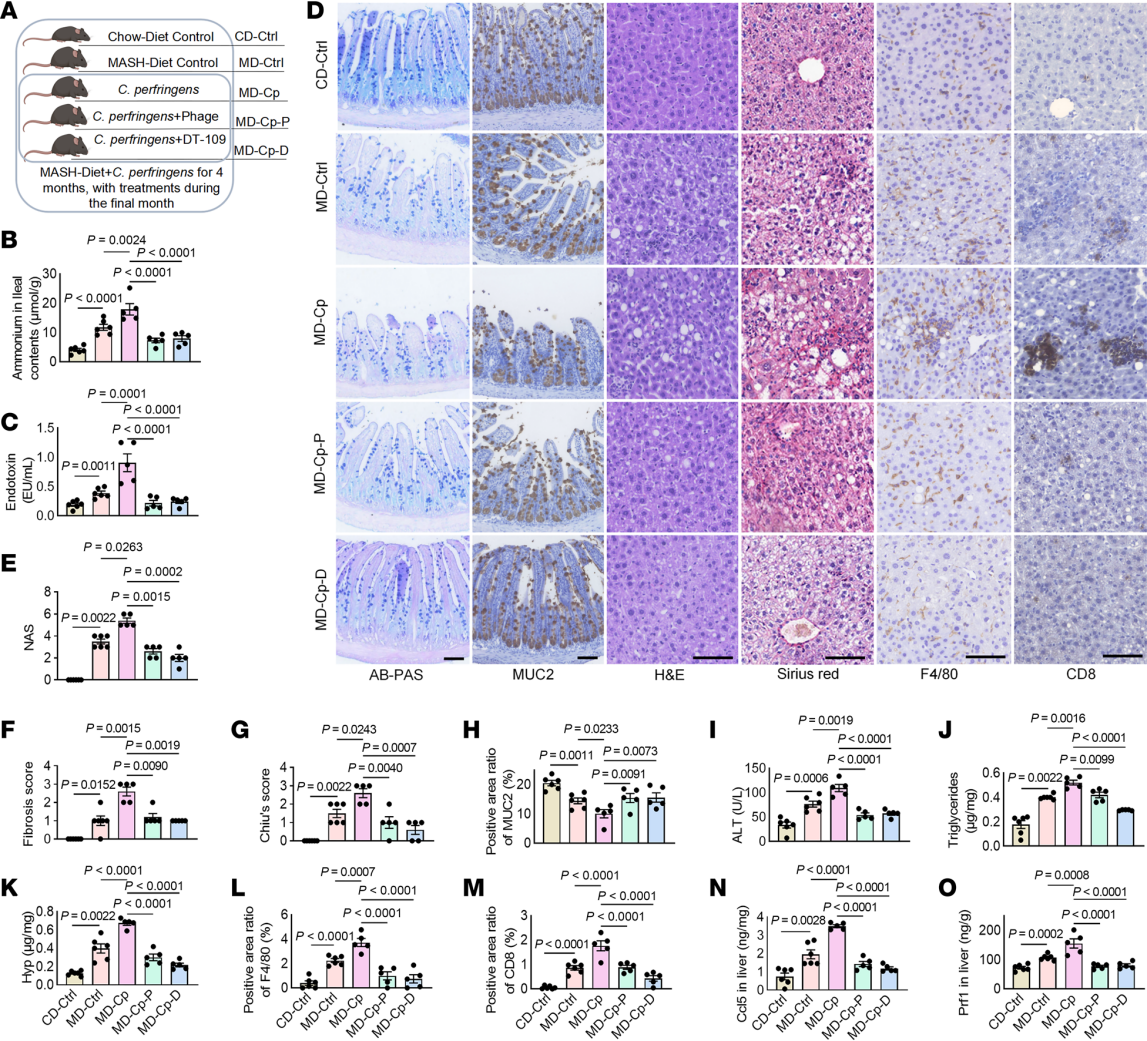
*C*. *perfringens*–directed therapeutics suppress MASH progression in mice. (**A**) Schematic of experimental design. C57BL/6 mice were fed a chow diet (CD-Ctrl) or MASH diet (all other groups). During the final month, MASH diet–fed mice coadministered *C*. *perfringens* received either solvent control (MD-Cp), bacteriophages targeting C. perfringens (MD-Cp-P), or DT-109 (MD-Cp-D). Control groups included MASH diet alone (MD-Ctrl) and chow diet (CD-Ctrl). Ileal ammonia (**B**) and plasma endotoxin (**C**). (**D**) Images of AB-PAS staining (ileum), MUC2 immunohistochemistry (ileum), H&E staining (liver), Sirius red staining (liver), F4/80 immunohistochemistry (liver), and CD8 immunohistochemistry (liver) across groups (scale bars, 100 μm). Liver NAS (**E**) and fibrosis score (**F**). Quantification of ileal mucosal damage by Chiu’s score (**G**) and MUC2 expression (**H**). Plasma ALT levels (**I**), hepatic triglyceride (**J**), and hydroxyproline content (**K**). Quantification of hepatic F4/80 macrophages (**L**) and CD8^+^ T cells (**M**). Hepatic Ccl5 (**N**) and Prf1 (**O**) levels across groups. Data (**B**, **C**, and **E**–**O**) are presented as means ± SEM. Statistical differences were analyzed using the Kruskal-Wallis test followed by Dunn’s post hoc test (**E**–**G**) or 1-way ANOVA followed by Dunnett’s post hoc test (**B**, **C**, and **H**–**O**). Values above brackets in figures represent *P* values unless indicated as FDR.

Given that ammonia drives CD8^+^ T cell–mediated liver injury by enhancing *CCL5* expression via FosB, its gut-derived origin emerges as a critical therapeutic target. To investigate this, C57BL/6 mice were fed a MASH diet with coadministration of *C*. *perfringens* for 4 months. During the final month, treatment groups received *C*. *perfringens*–targeting bacteriophages (MD-Cp-P), DT-109 (MD-Cp-D), or solvent control (MD-Cp). Control groups included mice fed the MASH diet alone (MD-Ctrl) and healthy chow-fed mice (CD-Ctrl) (Figure 8A). Compared to MD-Ctrl, the MD-Cp group exhibited significantly elevated ileal ammonia (Figure 8B), plasma endotoxin (Figure 8C), NAS and fibrosis scores (Figure 8, D–F), and Chiu’s scores (Figure 8, D and G). Additionally, the MD-Cp group showed reduced intestinal MUC2 expression (Figure 8, D and H) and increased plasma ALT (Figure 8I), hepatic triglyceride (Figure 8J), hydroxyproline (Figure 8K), F4/80 cell (Figure 8, D and L), CD8^+^ T cell (Figure 8, D and M), and Ccl5 (Figure 8N) and Prf1 (Figure 8O) levels, indicating exacerbated gut barrier damage and liver MASH. Both bacteriophage and DT-109 treatments significantly ameliorated adverse changes, reducing ileal ammonia, endotoxin, NAS, fibrosis, and Chiu’s scores, while increasing MUC2 expression and lowering plasma ALT, hepatic triglyceride, hydroxyproline, F4/80 cell, CD8^+^ T cell, Ccl5, and Prf1 levels (Figure 8, B–O). The results indicate that selective elimination of *C*. *perfringens* restores gut/liver axis homeostasis and suppresses the progression of MASH.

## Discussion

Despite recent advances in microbiome research that have identified associations with health or disease, determining whether these relationships are causal or merely correlative remains challenging. Increasing evidence supports the important role of gut microbiota in the progression of MASH (25). The gut microbiota shows promise as a noninvasive biomarker and therapeutic target for MASLD/MASH. However, interactions between the gut microbiota and the host are vast and complex, with current understanding remaining limited (25). Challenges such as low translational success and inconsistent reproducibility hamper microbiota research and its clinical application (26). The composition of microbiota differs greatly across various segments of the gastrointestinal tract (27). Interrelationships among gut microorganisms are highly complex and dynamic, and microbiota composition varies substantially among individuals and between different host species (28). Although mouse models are valuable for studying host-microbiota interactions, the substantial differences in gut microbiota between mice and humans limit the translation of research into the clinic (29). Therefore, there is a pressing need for an animal model that more closely resembles human pathology, particularly with respect to the gut microbiota. Direct data on ileal ammonia levels in human patients with MASH remain limited because of challenges in sampling, though elevated blood ammonia has been reported in clinical studies, contributing to disease progression via urea cycle dysregulation and hyperammonemia (15, 16, 30).

Our previous research developed a nonhuman primate model that closely mimics human MASH (18), and the gut microbiota of nonhuman primates was similar to that of humans (31). Building on this, we discovered that DT-109 can ameliorate MASH. However, the mechanisms underlying MASH and the therapeutic effects of DT-109 remain incompletely understood. Additionally, a limitation of most studies is that microbiota samples are typically derived from fecal matter (32). Strictly speaking, fecal microbiota cannot accurately reflect the gut’s internal microbiota composition. In this study, we address these limitations by using primate models and analyzing microbiota from in situ gut segment contents. This approach more closely simulates human conditions and allows for direct analysis of intestinal tissue and corresponding gut microbiota samples, providing a more accurate representation of the true microbial environment within the host’s intestines. This shift in sampling strategy accounts for the identification of key functional groups, such as *C*. *perfringens* here, complementing our prior findings on bile acid–related taxa and collectively demonstrating DT-109’s capacity to rectify multiple facets of gut microbial dysbiosis.

Damage to the intestinal barrier allows harmful bacteria and their molecules, such as endotoxins, to enter the liver, triggering the release of pro-inflammatory cytokines (33). Here, we found that endotoxin and d-lactate levels in the blood of MASH monkeys were significantly elevated, and treatment with DT-109 markedly reduced these levels. Pathological and transcriptional analysis revealed severe damage to the ileal barrier in the MASH group, which was markedly alleviated by DT-109 treatment. In contrast with alcohol-associated liver disease (ALD), where intestinal CD8^+^ T cell loss drives pathology and hepatic CD8^+^ T cells provide compensatory protection (34), our MASH models showed no significant changes in ileal CD8^+^ T cells. Ammonia treatment did not upregulate *CD8*, *FosB*, or *CCL5* in the intestine, and CD8^+^ T cell depletion did not affect ammonia-induced barrier integrity in mice. This indicates that ammonia’s damaging effects on the intestinal barrier are independent of intestinal CD8^+^ T cells, highlighting organ-specific immune responses in MASH versus ALD. This investigation of the CD8^+^ T cell–mediated hepatic axis complements our prior finding of macrophages (35), indicating DT-109’s broad immunomodulation.

Metagenomic analysis indicated dysregulation of ammonium nitrogen metabolism in the ileum of MASH. Both the ammonia-producing gene *NirA* and the ammonia-producing bacterium *C*. *perfringens* were more abundant in the MASH group compared with the normal and DT-109 groups. Correspondingly, intestinal ammonia levels were significantly elevated in the MASH group. Positive correlations between *C*. *perfringens* and intestinal ammonia levels, endotoxin levels, and NAS score indicate that high ammonia levels induced by *C*. *perfringens* may be a key driver in MASH progression. In vitro experiments, including monoculture, coculture, and phage trials, confirmed the ammonia-producing capability of *C*. *perfringens*, and DT-109 effectively reduced its abundance. Functional validation with *NirA*-knockout *C*. *perfringens* established a definitive causal relationship between bacterial ammonia production and the dual pathogenesis of intestinal barrier damage and hepatic CD8^+^ T cell–mediated inflammation. Previous studies have documented that high ammonia impairs tight junction barriers (36, 37), and our study confirms this, with ammonia administration in mice further demonstrating that elevated intestinal ammonia can damage the intestinal barrier. DT-109’s restoration of gut integrity thus links microbiota modulation (e.g., ammonia reduction) to liver protection by preventing harmful translocation and subsequent CD8^+^ T cell activation in the gut/liver axis. Pharmacokinetic studies further revealed that orally administered DT-109 rapidly accumulates in the gut, coinciding with a significant reduction in intestinal ammonia, providing direct in vivo evidence for its action. In our study, DT-109 remained below detection limits in plasma, indicating primarily gastrointestinal tract-localized action. The causal and pathogenic role of gut-derived ammonia was further confirmed by the abolition of ammonia-driven liver injury following CD8^+^ T cell depletion or CCL5 inhibition and by the protective effects of both *C*. *perfringens*-targeted bacteriophages and DT-109.

Previous research has reported that hepatic lipid accumulation can inhibit the expression of urea cycle enzymes, such as ornithine carbamoyltransferase, thereby obstructing the conversion of ammonia to urea and leading to ammonia accumulation (12). This hepatic ammonia accumulation can initiate and sustain hepatic inflammation and promote the progression of liver fibrosis (13, 38). Also, ammonia is closely associated with various multiorgan symptoms of MASH (39), including MASH-related hyperammonemia (12, 16), cognitive dysfunction (40), sarcopenia (41), and HCC (42). Apart from the liver, the intestine is an important source of ammonia. Recent studies have established a causal relationship between intestinal ammonia metabolism and disease progression (10, 11). For instance, *Fusobacterium nucleatum* can promote chromosomal instability and drug resistance in patients with multiple myeloma by stabilizing NEK2 protein through increased acetylation and decreased ubiquitination (11). Ruminants, as a unique group of animals with respect to nitrogen utilization, harbor high ammonia-producing bacteria in their rumen, which degrade proteins into ammonia, providing a primary nitrogen source for rumen microbes (43). Excessively high concentrations of ammonia in the rumen can disrupt homeostasis, leading to mortality (44). Although these bacteria, such as *Clostridium aminophilum* and *Peptostreptococcus anaerobius*, constitute less than 1% of the total microbial population, they play a crucial role (44, 45). Using selective culture methods, certain human gut bacteria capable of producing high levels of ammonia have been identified, including *C*. *perfringens* (22, 46, 47), the focus of our study. Our findings suggest that *C*. *perfringens*, an ammonia-producing bacterium, in the intestine contributes to MASH by damaging the intestinal barrier, highlighting a distinct pathogenic pathway for MASH. While hepatocytes are central to ammonia detoxification via the urea cycle, DT-109’s therapeutic effects act upstream by reducing gut-derived ammonia burden. Unlike GLP-1 receptor agonists that primarily exert effects through weight loss, DT-109 did not significantly alter body weight or plasma GLP-1 levels in nonhuman primates, indicating its benefits are mediated via non–GLP-1-dependent pathways targeting the gut/liver axis.

Rifaximin, an antibiotic, is commonly used as a treatment for hepatic encephalopathy (48) and hyperammonemia (30). Recent studies have demonstrated that rifaximin can ameliorate MASH by modulating gut microbiota and influencing bile acid production (49), and it has also been shown to improve hepatic fibrosis and enhance gut barrier integrity (50). Despite being classified as an ammonia-lowering agent, the mechanisms of action described above do not emphasize that it exerts its effects through the reduction of ammonia. Reduction of blood or hepatic ammonia levels with ornithine phenylacetate, an ammonia scavenger, prevented the progression of fibrosis (38). Here, our study indicates that reducing intestinal ammonia also plays a vital role in ameliorating MASH, suggesting that therapeutic strategies targeting gut ammonia may be beneficial. In this context, DT-109 appears to exert multifaceted effects: in addition to its antioxidant activity and modulation of lithocholic acid–related bile acid metabolism, it suppresses ammonia-producing gut bacteria, presenting a distinct therapeutic pathway for MASH. This finding also suggests DT-109’s potential for treating other hyperammonemia-related diseases.

The phenotype of MASH liver is characterized by lipid accumulation and elevated endotoxin levels (51), with a pro-inflammatory environment being a key pathological feature. Recent research has highlighted that CD8^+^ T cells undergo a series of activation steps leading to auto-aggression, which contributes to metabolic diseases such as MASH (5). Results from RNA-seq, scRNA-seq, and IHC revealed that immune responses mediated by CD8^+^ T cells are enhanced in the MASH liver, with significant upregulation of key markers and enrichment of pathways in CD8^+^ T cells, which confirms, from a nonhuman primate model, that CD8^+^ T cells exhibit cytotoxicity in MASH liver. Ammonia-induced hepatotoxicity exhibited CD8^+^ T cell dependency, as demonstrated by resistance in T cell–deficient and CD8^+^ T cell–depleted mice in the present study. Previous research has reported that CCL5 plays a crucial role in T cell recruitment and in activating T cells for immune attack (52). Prior studies have shown that CCL5 is highly expressed in the liver of patients with MASLD/MASH, and the increasing hepatic CCL5 promotes steatosis and inflammation (53). Our analysis revealed that CCL5 levels were significantly higher in patients with MASH compared with healthy controls (Figure 6D), underscoring the importance of CCL5 in MASH and highlighting the potential of CCL5 as a noninvasive diagnostic marker for MASH. To evaluate its specific utility in monitoring disease resolution, such as DT-109 therapy, further validation in longitudinal human interventional studies is required. CCL5 contributes to MASH pathogenesis by enhancing pro-inflammatory responses and activating hepatic stellate cells (HSCs), driving steatosis and fibrosis; for instance, CCL5 from HSCs induces hepatocyte steatosis via CCR5, and the CCL5/CCR5 axis promotes fibrosis (53, 54). Inhibition of CCL5 with maraviroc attenuated ammonia-induced pathology, similar to CD8^+^ T cell depletion. The increased CCL5 expression in CD8^+^ T cells was also demonstrated by the results of scRNA, immunofluorescence, and in vitro experiments in the present study, which supports the hypothesis that hepatic CD8^+^ T cell–derived CCL5 mediates CD8^+^ T cell cytotoxicity in MASH livers. Targeting CCL5 has already been applied in treatments for AIDS, cancer, and autoimmune diseases (55), and applying these CCL5-targeted therapies for MASH seems to be a promising strategy. Additionally, we found that DT-109 reduced CCL5 levels and CD8^+^ T cell cytotoxicity in the MASH monkeys, validating the pathway of DT-109 in ameliorating MASH and suggesting its potential in treating other CCL5-related diseases.

LPS in the gut/liver axis can modulate liver immunity by driving CD8^+^ T cells with immunomodulatory capabilities (56). High ammonia levels can induce metabolic reprogramming in T cells (57, 58). Environmental stimuli can alter the state and fate of T cells, but the underlying molecular mechanisms remain incompletely understood. In this study, we identified FosB as a key transcription factor that activates CCL5 expression in CD8^+^ T cells. Ammonia promotes *FosB* upregulation via Ca^2+^ influx, and ammonia induces calcium dysregulation potentially through NMDA receptor activation (59, 60). Ammonia does not directly alter CCL5 locus H3K27ac and H3K4me3 levels in CD8^+^ T cells, nor does *FosB* knockdown; instead, PA upregulates these marks, suggesting lipid toxicity drives chromatin remodeling while ammonia acts via FosB *trans*-regulation. Previous research has shown that FosB regulates T cell differentiation and transformation in response to stimuli or environmental stress (61, 62). Studies related to tuberculosis have demonstrated that FosB controls CCL5 expression in human alveolar epithelial cells by driving CCL5 promoter activity (63). FosB functions at enhancers that regulate gene expression critical to brain development and disease (64). In this study, we found that ammonia promotes FosB-driven CCL5 expression, demonstrating that *FosB* within CD8^+^ T cells is indispensable for ammonia-driven liver injury.

In conclusion, our study shows that disruptions in the gut microbiota led to an ammonia-stressed intestinal environment, resulting in gut barrier damage. Barrier disruption facilitates the translocation of gut-derived factors, including ammonia, to the liver, where they induce epigenetic and transcriptional reprogramming in hepatic CD8^+^ T cells. These alterations promote FosB-mediated CCL5 expression, driving CD8^+^ T cell cytotoxicity and contributing to MASH progression. Importantly, we demonstrate that DT-109 effectively ameliorates MASH by disrupting this pathogenic gut/liver axis.

## Methods

### Sex as a biological variable.

Human samples were deliberately sourced from male and female individuals, with approximately equal representation. For nonhuman primate and mouse experiments, to minimize estrogen-related confounding effects, only male animals were employed in this study. This decision was made because female mice exhibit greater interindividual variability in metabolic indices than males, largely due to the substantial impact of estrogen and estrogen receptor signaling on lipid metabolism in MASH.

All other methods are described in the Supplemental Methods.

### Statistics.

Unless otherwise specified, results from all experiments are presented as the mean ± SEM, with each experiment repeated a minimum of 3 times independently. Biological replications were conducted as indicated. Each data point reflects either an independent experiment or an individual subject. Prior to statistical comparisons, data were evaluated for equal variance and normality using the Shapiro-Wilk and Kolmogorov-Smirnov tests. If assumptions of normality and equal variance were confirmed, comparisons between 2 groups were made using an unpaired *t* test, and differences among multiple groups were assessed using 1-way ANOVA followed by Dunnett’s post hoc test. For data that did not meet these assumptions, nonparametric methods such as the Mann-Whitney *U* test or Kruskal-Wallis test, followed by Dunn’s post hoc test, were used. All statistical tests were 2-sided unless specified otherwise. A *P* value less than 0.05 was considered statistically significant. Comprehensive demographic, clinical, and biochemical data for cohorts are provided in Supplemental Tables 4 and 5.

### Study approval.

The study protocol involving humans, all amendments, and the informed consent form were reviewed and approved by the Institutional Review Boards at each site, including the First Affiliated Hospital of Xi’an Jiaotong University (approval: XJTU1AF2023LSK330), Jinan University (approval: 2016-017), and The University of Hong Kong/Hospital Authority Hong Kong West Cluster (approval number: UW 20–700). Written informed consent was obtained from all eligible participants before any study-related procedures were conducted. All experimental protocols involving nonhuman primates were approved by the Laboratory Animal Care Committee of Xi’an Jiaotong University (approval: 20191278) and the Institutional Animal Care and Use Committee of Spring Biological Technology Development (approval: 201901). All procedures performed on mice were approved (approval: 2024971) by the Laboratory Animal Care Committee of Xi’an Jiaotong University.

### Data availability.

All data are available in the paper and in its supplement. scRNA-seq of livers, RNA-seq of livers and ileum, and metagenome of the ileal contents have been deposited in NCBI’s GEO database (accession codes: GSE279424, PRJNA1177807, PRJNA1177814, and PRJNA1177826). Supporting data values used to generate the figures are provided in a separate Supporting Data Values file.

## Author contributions

PQ, J Zhang, EL, and YEC conceived the study. PQ, SD, Y Zhang, LH, RD, J Zhao, WC, CS, JQ, S Luo, YH, BW, SH, ZZ, OR, J Zhang, EL, and YEC conducted the investigation. PQ, Y Zhang, SD, ES, LH, RD, J Zhao, WC, CS, JQ, XX, S Luo, HH, YH, LB, S Zhao, BW, Ying Zhao, YG, ZZ, YW, QW, AX, LZ, WG, CD, S Liu, J Zhang, EL, and YEC developed the methodology. PQ, SD, Y Zhang, J Zhang, EL, and YEC performed the formal analysis. PQ, Yang Zhao, J Zhang, SD, and EL wrote the original draft. PQ, SD, Yang Zhao, J Zhang, EL, and YEC reviewed and edited the manuscript. PQ, LZ, BW, AX, EL, and YEC acquired funding.

## Conflict of interest

OR, JZ, and YEC have filed a patent application (PCT/US2019/046052). YEC is the founder of Diapin Therapeutics, which provided DT-109 for this study.

## Funding support

- Fundamental Research Funds for the Central Universities (grants xzy012025168, xtr052025019, and xxj032023021 to PQ; grant xzd012024038 to EL).
- Guangxi Science and Technology Program (grant 2025FNFN96725 to PQ).
- Fangchenggang Science and Technology Plan Project (grant AB23006037 to PQ).
- National Natural Science Foundation of China (grants 82270496 to EL and 82070649 to BW).
- National High Technology Research and Development Program of China (grants 2020YFA0803700 and 2023YFA1800904 to LZ).
- Natural Science Foundation of Beijing, China (grants L232031 and J230039 to LZ).
- Hong Kong Research Grant Council (grant AOE/M/707-18 to AX).
- Tang Scholar (to PQ).
- Frederick G.L. Huetwell Endowed Professor of Cardiovascular Medicine at the University of Michigan (to YEC).

## Supplementary Material

Supplemental data

Unedited blot and gel images

Supplemental tables 1-5

Supporting data values

## Version 1 05/12/2026

In-Press Preview

## Version 2 07/01/2026

Electronic publication

## References

[1] Riazi K (2022). The prevalence and incidence of NAFLD worldwide: a systematic review and meta-analysis. Lancet Gastroenterol Hepatol.

[2] Friedman SL (2018). Mechanisms of NAFLD development and therapeutic strategies. Nat Med.

[3] Harrison SA (2023). Challenges and opportunities in NASH drug development. Nat Med.

[4] Liu W (2024). SGLT2 inhibitor promotes ketogenesis to improve MASH by suppressing CD8(+) T cell activation. Cell Metab.

[5] Dudek M (2021). Auto-aggressive CXCR6^+^ CD8 T cells cause liver immune pathology in NASH. Nature.

[6] Pfister D (2021). NASH limits anti-tumour surveillance in immunotherapy-treated HCC. Nature.

[7] Yahoo N (2023). Role of immune responses in the development of NAFLD-associated liver cancer and prospects for therapeutic modulation. J Hepatol.

[8] Pendyala S (2012). A high-fat diet is associated with endotoxemia that originates from the gut. Gastroenterology.

[9] Chen B (2022). Gut bacteria alleviate smoking-related NASH by degrading gut nicotine. Nature.

[10] Wang P (2023). Gut microbiome-derived ammonia modulates stress vulnerability in the host. Nat Metab.

[11] Jian X (2020). Alterations of gut microbiome accelerate multiple myeloma progression by increasing the relative abundances of nitrogen-recycling bacteria. Microbiome.

[12] De Chiara F (2018). Urea cycle dysregulation in non-alcoholic fatty liver disease. J Hepatol.

[13] Jalan R (2016). Ammonia produces pathological changes in human hepatic stellate cells and is a target for therapy of portal hypertension. J Hepatol.

[14] Thomsen KL (2023). Role of ammonia in NAFLD: An unusual suspect. JHEP Rep.

[15] Simon J (2020). Targeting hepatic glutaminase 1 ameliorates non-alcoholic steatohepatitis by restoring very-low-density lipoprotein triglyceride assembly. Cell Metab.

[16] Mercado-Gomez M (2024). The lipopolysaccharide-TLR4 axis regulates hepatic glutaminase 1 expression promoting liver ammonia build-up as steatotic liver disease progresses to steatohepatitis. Metabolism.

[17] Rom O (2020). Glycine-based treatment ameliorates NAFLD by modulating fatty acid oxidation, glutathione synthesis, and the gut microbiome. Sci Transl Med.

[18] Qu P (2023). DT-109 ameliorates nonalcoholic steatohepatitis in nonhuman primates. Cell Metab.

[19] Jia L (2025). Tripeptide DT-109 (Gly-Gly-Leu) attenuates atherosclerosis and vascular calcification in nonhuman primates. Signal Transduct Target Ther.

[20] Rom O (2022). Induction of glutathione biosynthesis by glycine-based treatment mitigates atherosclerosis. Redox Biol.

[21] Zhang J (2013). A tripeptide Diapin effectively lowers blood glucose levels in male type 2 diabetes mice by increasing blood levels of insulin and GLP-1. PLoS One.

[22] Richardson AJ (2013). Ammonia production by human faecal bacteria, and the enumeration, isolation and characterization of bacteria capable of growth on peptides and amino acids. BMC Microbiol.

[23] Grenda T (2023). *Clostridium perfringens*-opportunistic foodborne pathogen, its diversity and epidemiological significance. Pathogens.

[24] Fenn S (2021). NirA is an alternative nitrite reductase from pseudomonas aeruginosa with potential as an antivirulence target. mBio.

[25] Sharpton SR (2021). Current concepts, opportunities, and challenges of gut microbiome-based personalized medicine in nonalcoholic fatty liver disease. Cell Metab.

[26] Porcari S (2023). Key determinants of success in fecal microbiota transplantation: From microbiome to clinic. Cell Host Microbe.

[27] Martinez-Guryn K (2019). Regional diversity of the gastrointestinal microbiome. Cell Host Microbe.

[28] Chen L (2021). The long-term genetic stability and individual specificity of the human gut microbiome. Cell.

[29] Rosshart SP (2019). Laboratory mice born to wild mice have natural microbiota and model human immune responses. Science.

[30] Eriksen PL (2023). Clearance and production of ammonia quantified in humans by constant ammonia infusion - the effects of cirrhosis and ammonia-targeting treatments. J Hepatol.

[31] Li X (2018). Establishment of a Macaca fascicularis gut microbiome gene catalog and comparison with the human, pig, and mouse gut microbiomes. Gigascience.

[32] Shalon D (2023). Profiling the human intestinal environment under physiological conditions. Nature.

[33] Gabele E (2011). DSS induced colitis increases portal LPS levels and enhances hepatic inflammation and fibrogenesis in experimental NASH. J Hepatol.

[34] Maccioni L (2025). Opposite regulation of intestinal and intrahepatic CD8^+^ T cells controls alcohol-associated liver disease progression. Gut.

[35] Qu PX (2023). DT-109 ameliorates nonalcoholic steatohepatitis in nonhuman primates. Cell Metab.

[36] Yokoo K (2021). Ammonia impairs tight junction barriers by inducing mitochondrial dysfunction in Caco-2 cells. FASEB J.

[37] Zhang X (2022). Intestinal barrier dysfunction induced by ammonia exposure in pigs in vivo and in vitro: The protective role of L-selenomethionine. Ecotoxicol Environ Saf.

[38] De Chiara F (2020). Ammonia scavenging prevents progression of fibrosis in experimental nonalcoholic fatty liver disease. Hepatology.

[39] Gallego-Duran R (2024). Ammonia-induced stress response in liver disease progression and hepatic encephalopathy. Nat Rev Gastroenterol Hepatol.

[40] Kjaergaard K (2021). Cognitive dysfunction in non-alcoholic fatty liver disease-current knowledge, mechanisms and perspectives. J Clin Med.

[41] Carias S (2016). Nonalcoholic steatohepatitis is strongly associated with sarcopenic obesity in patients with cirrhosis undergoing liver transplant evaluation. J Gastroenterol Hepatol.

[42] Zhang Q (2024). Deficiency in SLC25A15, a hypoxia-responsive gene, promotes hepatocellular carcinoma by reprogramming glutamine metabolism. J Hepatol.

[43] Liu S (2023). Functional gene-guided enrichment plus in situ microsphere cultivation enables isolation of new crucial ureolytic bacteria from the rumen of cattle. Microbiome.

[44] Patra AK, Aschenbach JR (2018). Ureases in the gastrointestinal tracts of ruminant and monogastric animals and their implication in urea-N/ammonia metabolism: A review. J Adv Res.

[45] Attwood GT (1998). Ammonia-hyperproducing bacteria from New Zealand ruminants. Appl Environ Microbiol.

[46] Hasan SM, Hall JB (1975). The physiological function of nitrate reduction in Clostridium perfringens. J Gen Microbiol.

[47] Zheng M (2020). Metagenome analyses reveal the role of Clostridium perfringens in alfalfa silage anaerobic deterioration. FEMS Microbiol Lett.

[48] Bass NM (2010). Rifaximin treatment in hepatic encephalopathy. N Engl J Med.

[49] Jian J (2022). Rifaximin ameliorates non-alcoholic steatohepatitis in mice through regulating gut microbiome-related bile acids. Front Pharmacol.

[50] Enomoto M (2022). Rifaximin and lubiprostone mitigate liver fibrosis development by repairing gut barrier function in diet-induced rat steatohepatitis. Dig Liver Dis.

[51] Carpino G (2020). Increased liver localization of lipopolysaccharides in human and experimental NAFLD. Hepatology.

[52] Murooka TT (2008). CCL5-mediated T-cell chemotaxis involves the initiation of mRNA translation through mTOR/4E-BP1. Blood.

[53] Kim BM (2018). Hepatic stellate cells secrete Ccl5 to induce hepatocyte steatosis. Sci Rep.

[54] Li BH (2017). Steatosis induced CCL5 contributes to early-stage liver fibrosis in nonalcoholic fatty liver disease progress. Transl Res.

[55] Zeng Z (2022). CCL5/CCR5 axis in human diseases and related treatments. Genes Dis.

[56] Pallett LJ (2023). Tissue CD14^+^CD8^+^ T cells reprogrammed by myeloid cells and modulated by LPS. Nature.

[57] Bell HN (2023). Microenvironmental ammonia enhances T cell exhaustion in colorectal cancer. Cell Metab.

[58] Zhang H (2024). Ammonia-induced lysosomal and mitochondrial damage causes cell death of effector CD8^+^ T cells. Nat Cell Biol.

[59] Deitmer JW (1993). Independent changes of intracellular calcium and pH in identified leech glial cells. Glia.

[60] Cai Z (2019). Ammonia induces calpain-dependent cleavage of CRMP-2 during neurite degeneration in primary cultured neurons. Aging (Albany NY).

[61] Baumann S (2003). An unexpected role for FosB in activation-induced cell death of T cells. Oncogene.

[62] Delpoux A (2021). FOXO1 constrains activation and regulates senescence in CD8 T cells. Cell Rep.

[63] Wickremasinghe MI (2004). Transcriptional mechanisms regulating alveolar epithelial cell-specific CCL5 secretion in pulmonary tuberculosis. J Biol Chem.

[64] Malik AN (2014). Genome-wide identification and characterization of functional neuronal activity-dependent enhancers. Nat Neurosci.

