# Supplementary material for: Metabolic dysfunction–associated steatohepatitis exacerbated by Clostridium perfringens–derived ammonia is attenuated by tripeptide DT-109
Source: J Clin Invest. 2026 May 12;136(13):e200522. doi: 10.1172/JCI200522 (PMC13318118; doi:10.1172/JCI200522)
Supplement: Supplemental data [file jci-136-200522-s202.pdf]

Supplementary Materials for

Metabolic dysfunction-associated steatohepatitis exacerbated by Clostridium perfringens-derived ammonia is attenuated by tripeptide DT-109

Pengxiang Qu[1#,*], Shusi Ding[2#], Yanru Zhang[1#], Yang Zhao[3], Erfei Song[4], Liangshuo Hu[5], Ruike Ding[1], Wenbin Cao[1], Yiting Hou[1], Jia Qi[1], Juan Zhao[1,6], Chenjing Duan[1], Shuangqing Liu[1], Chong Shen[1], Ying Zhao[3], Yanhong Guo[3], Zuowen Zheng[7], Shiwei Luo[1], Huizhong Hu[1], Liang Bai[1], Sihai Zhao[1], Bo Wang[5], Shuixiang He[1], Yi Wu[1], Xuelian Xiong[8], Qiutong Wu[9], Weiwang Gu[10], Oren Rom[11], Aimin Xu[12], Lemin Zheng[2,9*], Jifeng Zhang[3*], Enqi Liu[1*], Y. Eugene Chen[3*]

*** Corresponding authors**

Correspondence to qupengxiang@xjtu.edu.cn (P.Q.); zhengl@bjmu.edu.cn (L.Z.); jifengz@umich.edu (J.Z.); liuenqi@xjtu.edu.cn (E.L.); and echenum@umich.edu (Y.E.C)

Content:

Details of Materials and Methods

Supplemental figures S1-S14

Details of Materials and Methods

Human Participants

This study comprised two patient cohorts. Cohort 1 included a total of 111 subjects (44 healthy individuals and 67 diagnosed with MASH), as characterized by plasma CCL5 levels determined using Olink data, sourced from a recent study (1). Subjects with a NAS of 4 or greater were classified as the MASH group, while those with a NAS of 0 were classified as the healthy group. Inclusion and exclusion criteria were as previously reported (1). Additionally, serum samples from 45 subjects (20 healthy and 25 with MASH) were randomly selected from this cohort and validated for CCL5 levels by ELISA.

For cohort 2, liver samples were collected from 12 female subjects (6 healthy individuals and 6 with MASH) at the First Affiliated Hospital of Xi'an Jiaotong University. The inclusion criteria for this cohort were: (1) age greater than 18 years; (2) alcohol consumption of less than 70 g/week for females. The exclusion criteria were: the presence of diabetic ketoacidosis or hyperglycemic hyperosmolar state; decompensated cirrhosis; stage 3-5 chronic kidney disease; cancer; pulmonary tuberculosis; AIDS; alcoholism; viral hepatitis; autoimmune liver disease; and drug-induced liver disease. The diagnosis of MASH and healthy status was determined as described in cohort 1.

Nonhuman Primate Experiments

Twenty male monkeys were fed a MASH diet for 10 months, then randomized to receive DT-109 (150 mg/kg body weight/day by gavage) or an equivalent volume of H_2O for 5 additional months on the MASH diet. The MASH diet is composed of monkey maintenance feed (40.5%), casein (7%), lard (15%), egg yolk powder (15%), cholesterol (1%), fructose (13.4%), sucrose (4.3%), dicalcium phosphate (2.2%), and vitamins and minerals (1.6%). Six male monkeys were fed a chow diet during the 15 months. The schedule of the three groups was shown in Fig. S1a. At the endpoint, blood samples were collected, and the monkeys were then anesthetized as previously described (2). Livers were quickly removed and then perfused with cold phosphate-buffered saline (PBS). The livers were then divided into peanut-sized tissue blocks for immediate scRNA-seq, and the remaining liver tissues were stored in liquid nitrogen or formalin. The ileum was quickly removed, and the content in the ileum was collected for ammonia detection, metagenomic, and targeted metabolomics. And the ileum tissues were stored in liquid nitrogen or formalin.

Mouse Experiments

All animal procedures were approved by the Institutional Animal Care and Use Committee of Xi'an Jiaotong University Health Science Centre. Mice were maintained under specific-pathogen-free conditions with a chow diet unless specified. For Fig. 4N, C57BL/6 mice were treated with an antibiotic cocktail (ampicillin 0.2 g/L, vancomycin 0.1 g/L, neomycin 0.2 g/L, metronidazole 0.2 g/L) for 2 weeks, followed by *C. perfringens* or *C. perfringens* plus phages (10^8 CFU/mL, 200 μL/mouse, twice weekly) for 2 weeks. For Fig. 4U-V, C57BL/6 mice were orally administered DT-109 (450 mg/kg/day), with DT-109 and ammonia levels measured at specified time points. For Fig. 5A, C57BL/6 mice were treated with the same antibiotic cocktail for 2 weeks, then divided into groups receiving *C. perfringens*, inactivated *C. perfringens*, *C. perfringens* ΔNirA, or *C. ljungdahlii* under the same dosing regimen. For Fig. 5I, C57BL/6 mice were divided into groups receiving ammonium chloride (0.30 mg/g/day by gavage) or H_2O for 8 weeks. For Fig. 7H, untreated $CD8^+$ T cells, ammonia-exposed (for 24 h) siCtrl-transfected $CD8^+$ T cells, or siFosB-transfected $CD8^+$ T cells were transferred into nude mice; analysis at 48 h post-transfer. For Fig. 8A, C57BL/6 mice were fed a chow diet (CD-Ctrl group) or MASH diet (with 40% fat-derived energy, 20% fructose (w/w), and 2% cholesterol (w/w)) for 4 months, with MASH diet-fed mice co-administered *C. perfringens* under the same dosing regimen receiving solvent control, bacteriophages, or DT-109 in the final month. For Fig. S11A, C57BL/6 mice were fed a MASH diet for 3 months, transplanted with *C. perfringens*, *C. perfringens* ΔNirA, or *C. ljungdahlii* under the same dosing regimen or left untreated in the final month. For Fig. S13A, Balb/c mice and Balb/c-nu were treated with ammonium chloride under the same dosing regimen for 1 week, while untreated Balb/c mice served as controls. For Fig. S13B, C57BL/6 mice were administered no ammonia (Non-A group), control IgG (200 μg/mouse, i.v.) plus ammonium chloride, or $CD8^+$ T cell-depleting antibody (200 μg/mouse, i.v.) plus ammonium chloride for 1 week. For Fig. S13C, C57BL/6 mice were fed a MASH diet for 3 months, with mice receiving ammonium chloride and IgG control, $CD8^+$ T cell-depleting antibody, PBS control, or the CCR5 antagonist Maraviroc (10 mg/kg, i.p., 3/week) in the final month. At the endpoint of those experiments, mice were euthanized, ileum contents were collected for the measurement of ammonia, and blood from the portal vein was collected for the measurement of plasma endotoxin. Plasma samples were collected for the measurement of AST, ALT, ammonia, and related parameters. Frozen liver tissue samples were collected for the quantification of TG, hydroxyproline, PRF1, CCL5, and other relevant markers. Formalin-fixed ileum and liver tissues were collected for H&E and immunohistochemistry.

Histological Analyses

Formalin-fixed tissues were processed through graded alcohols and cleared with xylene, followed by infiltration with molten paraffin using an automated tissue processor (TissueTek, Sakura-Americas). Using a Histostar Embedding Station (ThermoFisher Scientific), tissues were then sectioned on a M355S rotary microtome (ThermoFisher Scientific) at 4 μm and mounted on glass slides. Slides were stained for hematoxylin and eosin (H&E, ThermoFisher Scientific). Masson staining of the liver was performed using Masson's Trichrome Stain Kit (Solarbio, G1340) according to the instructions. The Masson’s Trichrome staining was used to score hepatic fibrosis from 0-4 (0: no fibrosis; 1: perisinusoidal or portal fibrosis; 2: perisinusoidal and portal fibrosis; 3: bridging fibrosis; 4: cirrhosis). The scores were independently evaluated by at least two independent pathologists who were blinded to experimental groups. Alcian Blue Periodic Acid Schiff (AB-PAS) Staining of the ileum was performed using AB-PAS Stain Kit (Solarbio, G1285) according to the instructions. Ammonia accumulation in liver sections was quantified using Nessler staining reagent (Sigma, MO, USA), and the ammonia score was determined based on the area ratios: 0-2 (Score 1), 2-10 (Score 2), 10-20 (Score 3), 20-30 (Score 4), 30-40 (Score 5), and >40 (Score 6), as previously described (3, 4).

Immunohistochemistry and immunofluorescence were performed on paraffin-embedded liver or ileum sections. Samples were heated in a microwave oven for 10 min in antigen retrieval buffers, then placed in 3% hydrogen peroxide for 15 min to quench endogenous peroxide. After washing with PBS (5 min/wash, three times) and blocking with goat serum (Absin, Shanghai, China) for 1 h, the sections were incubated with anti-MUC2 (1:200, ABclonal, A14659, Hubei, China), or anti-Occludin (1:200, ABclonal, A2601), or anti-MMP9 (1:200, ABclonal, A0289), or anti-TLR4 (1:150, ABclonal, A5258), or anti-NF-κB1 (1:100, ABclonal, A6667), or anti-CD68 (1:100, ABclonal, A22329), or anti-F4/80 (1:200, Abcam, ab16911, Boston, MA, USA), or anti-CD45 (1:100, ABclonal, A19021), or anti-CD3 (1:200, ABclonal, A1238), or anti-CD8 (1:200, ABclonal, A0663), or anti-Perforin (1:100, ABclonal, A0093), or anti-CCL5 (1:200, ABclonal, A14192), or anti-CD25 (1:200, ABclonal, A13253) at 4°C for 12 h, and washed with PBS (5 min/wash, three times). The sections were then incubated with enzyme-labeled goat anti-rabbit IgG (1:200, ABclonal, AS014) or goat anti-rat IgG (1:200, absin, abs20031, Beijing, China) for 1 h at room temperature, and washed with PBS (5 min/wash, three times). Finally, the sections were visualized using a 3, 3-diaminobenzidine (DAB) kit (ZSGB-BIO, ZLI-

9018, Beijing, China) and counterstained with hematoxylin. And for the immunofluorescence, primary antibody-stained sections were then incubated with 488-conjugated Goat anti-Rabbit IgG (1:500, ABclonal, AS053) or Cy3-conjugated Goat anti-Mouse IgG (1:500, ABclonal, AS008) for 1 h at room temperature, then incubated with DAPI for 5 min, and washed with PBS (5 min/wash, three times). Images were captured under a light microscope (Olympus, Tokyo, Japan) and quantified using Image-Pro plus (Media Cybernetics, Silver Springs, MD, USA). Western blot analyses were performed to quantify MUC2, Occludin, CD8, CCL1 and FOSB for additional validation.

scRNA-seq of Livers from Cynomolgus Monkeys

Liver samples for scRNA-seq were first washed with PBS, minced into small pieces (approximately 1mm^3) on ice, and enzymatically digested with 2 mg/mL collagenase II (Worthington) and 50 U/mL DNase I for 20 min at 37°C, with agitation. After digestion, the samples were thoroughly resuspended by pipetting, then centrifuged at 50 g for 2 minutes, and the supernatant was retained. Subsequently, the samples were sieved through a 70μm cell strainer, and centrifuged at 300 g for 5 min. After the supernatant was removed, the pellet was suspended in red blood cell lysis buffer (Miltenyi Biotec) to lyse red blood cells. After washing with PBS containing 0.04% BSA, the cell pellets were resuspended in PBS containing 0.04% BSA and re-filtered through a 35μm cell strainer. Dissociated single cells were then stained with AO/PI for viability assessment using a Countstar Fluorescence Cell Analyzer.

The scRNA-Seq libraries were generated using the 10X Genomics Chromium Controller Instrument and Chromium Single Cell 3’ V3.1 Reagent Kits (10X Genomics, Pleasanton, CA). Briefly, cells were concentrated to approximately 1,000 cells/uL and loaded into each channel to generate single-cell Gel Bead-In-Emulsions (GEMs). After the RT step, GEMs were broken, and barcoded cDNA was purified and amplified. The amplified barcoded cDNA was fragmented, A-tailed, ligated with adaptors and index PCR amplified. The final libraries were quantified using the Qubit High Sensitivity DNA assay (Thermo Fisher Scientific), and the size distribution of the libraries was determined using a High Sensitivity DNA chip on a Bioanalyzer 2200 (Agilent). All libraries were sequenced by an Illumina sequencer (Illumina, San Diego, CA) on a 150 bp paired-end run.

Fastp was applied to filter and eliminate adaptor sequences as well as remove low-quality reads. The Seurat package was utilized for cell normalization and

filtering, with criteria based on MT percentage, minimum, and maximum gene numbers. Principal component analysis (PCA), t-distributed stochastic neighbor embedding (t-SNE), and uniform manifold approximation and projection (UMAP) were employed to describe single-cell relationships. Graphcluster and K-means clustering were used for cell clustering, and the Wilcoxon rank sum test was applied in marker gene analysis. Differential screening algorithms were employed to calculate the Marker gene groups of single-cell clusters. Through these marker genes, cellular populations associated with each cell cluster were inferred and identified. Fisher's exact test assigned significance scores to the cell types based on the marker genes recorded in the CellMarker database. The Seurat4RDSPlot, implemented in R language, facilitated personalized visualization of single-cell UMAP/t-SNE plots, violin plots, and bubble plots by selecting specific genes, cells, or special groupings. Pathway analysis was conducted using the KEGG database to identify significant pathways of differential genes. Significant pathways were selected using Fisher's exact test, with the threshold of significance defined by FDR. The SCENIC (Single-Cell rEgulatory Network Inference and Clustering) analysis was performed on filtered cells using the 20,000 motifs database for RcisTarget and GRNboost. Monocle2 was utilized for pseudotime analysis based on the rds file analyzed by the Seurat package, which included clustering and cell marker identification. This analysis allowed for the examination of cell processing states and placed single cells along a trajectory according to a biological process such as cell differentiation, taking advantage of individual cells' asynchronous progression through these processes. The cell population of all identified cell types detected in our scRNA-seq assay is provided in the Table. S3.

RNA-sequencing of Livers or Ileums from Cynomolgus Monkeys

Approximately 50 mg of tissue was pulverized into a fine powder using liquid nitrogen, after which it was transferred into a 2 mL microtube pre-loaded with 1.5 mL of Trizol reagent (Invitrogen, Carlsbad, CA, USA). The resulting mixture was subjected to centrifugation at 12,000 g for 5 min at 4°C. Subsequently, the supernatant was carefully decanted into a fresh microtube containing 0.3 mL of chloroform/isoamyl alcohol (24:1) per 1.5 mL of Trizol reagent. Following a second centrifugation step at 12,000 g for 10 min at 4°C, the aqueous phase was isolated and transferred to a new microtube. An equal volume of isopropyl alcohol was then added. After further centrifugation at 12,000 g for 20 min at 4°C, the resulting supernatant was discarded, and the RNA pellet was washed with 1 mL of 75% ethanol. The RNA pellet was subsequently air-dried and dissolved in 100 µL of

DEPC-treated water. Quality and quantity of the RNA were assessed using a Bioanalyzer (Agilent 2100). RNase H was employed to selectively eliminate ribosomal RNA (rRNA) contamination. Purified mRNA was then fragmented, and first-strand cDNA synthesis was initiated within a First Strand Reaction System through PCR amplification, followed by the synthesis of second-strand cDNA. The resultant reaction product underwent purification, and subsequent steps involved A-Tailing Mix and RNA Index Adapters for end repair. The resultant products were amplified to generate the final library. The final library was sequenced on the BGISEQ500 platform (BGI, Shenzhen, China).

The raw data were preprocessed using SOAPnuke (v1.6.5). This involved a three-step filtering process: firstly, the removal of reads containing adapters to mitigate potential adapter contamination; secondly, the exclusion of reads exhibiting a ratio of unknown bases exceeding 1%; and thirdly, the elimination of reads characterized by a low-quality base ratio surpassing 40%. Subsequently, the resulting clean reads were acquired and stored in FASTQ format. The clean data underwent alignment to the reference genome (GCF000364345.1_macaca_fascicularis_5.0) employing HISAT (v2.2.1), followed by mapping to assembled unique genes utilizing Bowtie2 (v2.4.5). Quantification of gene expression levels was conducted utilizing RSEM (v1.3.1), supplemented by gene annotation utilizing public databases such as KEGG and GO. Differential gene expression analysis between groups was executed utilizing DEseq2, imposing criteria of Fold Change > 2 and FDR < 0.01. Furthermore, Gene Set Enrichment Analysis (GSEA) was performed based on gene expression data.

Gene transcription in human ileitis samples (GSE16879) retrieved from the GEO database was evaluated. GEO2R was utilized to generate log2 fold change data between inflamed ileum mucosa (18 patients) and normal ileum mucosa (n=6) employing all available replicates with default parameters. GSEA was subsequently conducted utilizing the preranked method, employing the clusterProfiler package in R. Specifically, curated gene sets (C2) from the MSigDB (Molecular Signatures Database) were analyzed. Additionally, estimation of immune cell abundance was performed utilizing the ImmuCellAI tool(5) (http://bioinfo.life.hust.edu.cn/ImmuCellAI), based on RNA-Seq.

Metagenome

Microbial genomic DNA samples were extracted using the OMEGA Mag-Bind Soil DNA Kit (M5635-02, Omega Bio-Tek, Norcross, GA, USA), following the

manufacturer's guidelines, and stored at -20°C for subsequent analysis. The libraries were prepared with TruSeq Nano DNA LT Library Preparation Kit (FC-121, Illumina, San Diego, CA, USA), which were then sequenced using the Illumina NovaSeq platform with a PE150 strategy at Metabo-Profile Biotechnology Co., Ltd. (Shanghai, China). The raw sequencing reads were processed to obtain high-quality reads for further analysis. Adapters were removed using Cutadapt (v1.2.1), and low-quality reads were trimmed with fastp's sliding-window algorithm. Host contamination was minimized by aligning reads to the host genome using BMTagger. Quality-filtered reads were then taxonomically classified using Kraken2 or Kaiju against RefSeq and NR-derived databases, respectively. Contigs longer than 300 bp were assembled and clustered, and their taxonomy was determined by aligning them against the NCBI-nt database. Genes within the contigs were predicted using MetaGeneMark, and CDS sequences were clustered using mmseqs2. Gene abundances were assessed by mapping high-quality reads onto the predicted gene sequences. Non-redundant genes were functionally annotated using mmseqs2 against KEGG. Beta diversity was assessed to explore the variation in microbial community composition and function across samples, employing Bray-Curtis distance metrics and visualized through PCoA, NMDS, and UPGMA hierarchical clustering. In addition, the taxonomic and functional profiles of non-redundant genes were analyzed using LEfSe to identify differentially abundant taxa and functions across the groups. Microbial function was predicted with FAPROTAX. Significantly different species, KOs and functional profiles of FAPROTAX were analyzed using DESeq2 (version 1.26.0), and the difference that was significant was determined with the absolute value of log base 2 of fold change > 1 and FDR < 0.05. The Spearman’s correlation in this study was calculated and visualized with Wekemo Bioincloud (6) (https://bioincloud.tech/).

Biochemical Analyses for Human Samples

Serum samples from cohort 1 were collected from overnight fasting patients and stored at −80°C for Olink proteomics analysis (Uppsala, Sweden) and ELISA. The relative level of CCL5 in human serum samples based on the Olink assay, and indicated with NPX (Normalized Protein eXpression). The absolute content of CCL5 in human serum samples from cohort 1 was detected using Human CCL5/RANTES Immunoassay ELISA kit (DRN00B).

Biochemical Analyses for Monkey Samples

Thirty-five cytokines or chemokines or growth factors (such as CCL5, IFN-

gamma, PD-L1, and CD40L) in monkey livers were detected using NHP XL Cytokine Premixed Kit (R&D Systems, FCSTM21, Minneapolis, USA) under Luminex 200 analyzer according to instructions. Concentrations of liver cytokines or chemokines or growth factors were normalized to total protein in liver tissues. CCL5 in monkey plasma was detected using the NHP XL Cytokine Premixed Kit. Endotoxin in monkey plasma was detected using the Endotoxin Assay Kit (Bioendo Technology Co., Ltd. EC64405S, Xiamen, China). D-lactic acid in monkey plasma was detected using D-Lactic Acid Colorimetric Assay Kit (Elabscience Biotechnology Co., Ltd. E-BC-K002-M, Wuhan, China). Ammonia in plasma or ileal contents was detected using an Ammonia Assay Kit (Abcam, ab83360) with a colorimetric detection method on a microplate reader according to instructions.

Biochemical Analyses for Mouse Samples

Mice were anesthetized by intraperitoneal injection of avertin (300 mg/kg). The abdominal cavity of the mice was opened to expose the portal vein, and blood was collected from the portal vein using a 1 mL syringe. After obtaining whole blood, plasma was quickly extracted. Subsequently, sodium pentobarbital (150 mg/kg) was used to euthanize the mice by intravenous injection, and the contents of the ileum were immediately collected. Endotoxin in mouse plasma was detected using Endotoxin Assay Kit (Bioendo Technology Co., Ltd). Ammonia in plasma or the ileal contents of mice was detected using an Ammonia Assay Kit (Solarbio, BC4385, Beijing, China) according to instructions.

Culture of *C. perfringens*

Reinforced Medium for *Clostridia* (Hopebio, HB0316, Tsingtao, China) was sterilized by boiling prior to use. The oxygen scavenger cysteine hydrochloride (Harveybio, AA1013, Beijing, China) was added and nitrogen gas was passed through to facilitate the removal of oxygen. An oxygen indicator resazurin (Zikerbio, ZK-L1084, Shenzhen, China) was added at a final concentration of 0.1 mg/100 ml until the medium changed color from blue to pink and eventually became colorless. This indicated that the medium had become anaerobic. Both the medium and anaerobic tubes were autoclaved before use. *C. perfringens* strains were obtained from BeNa Culture Collection (BNCC364172). After inoculating *C. perfringens* into the culture medium, the bacteria were incubated in a constant temperature anaerobic incubator. The absorbance of bacterial suspension was measured using a spectrophotometer and an OD_{600} value of 0.4 indicated logarithmic phase growth suitable for subculture or intervention experiments.

An appropriate amount of bacterial solution was mixed with DT-109 (the control group received an equal amount of ddH_20) to achieve a final concentration of 1 mM DT-109. The bacterial solution was then cultured in an anaerobic incubator and samples were taken at five time points: 0, 2, 4, 8, and 12 h for OD_{600} absorbance detection as well as ammonia content detection, which reflected the degree of bacterial growth.

Generation of *C. perfringens* Δ*NirA* Isogenic Mutants Strain

All *C. perfringens* strains were cultured in Reinforced Medium for Clostridium (Hopebiol, Shandong, China) under anaerobic conditions for liquid growth. To construct the ΔnirA mutants, two primer pairs—nirA-up-F/ nirAL-5′RXS and nirA-dn-R/cagAF-3′FXS—were used to amplify the upstream and downstream regions of the nirA locus, respectively. These fragments were fused by overlap-extension PCR to generate the nirAdel amplicon, which excludes the nirA coding sequence but retains both homology arms. A 0.7 kb chloramphenicol-acetyltransferase (cat) cassette was inserted into a central SmaI site within nirAdel, yielding a unilateral homologous recombination construct capable of replacement in the C. perfringens genome. The recombination plasmid was introduced into C. perfringens by electroporation. Mid-log-phase cells were washed twice with ice-cold 10% glycerol and resuspended in 10% glycerol. A 50 µL aliquot was mixed with ~1 µg plasmid DNA and pulsed in a 0.2 cm electroporation cuvette at 1.8 kV, 200 Ω, and 25 µF. After electroporation, cells were recovered in liquid medium for 2 hours, then plated onto sheep blood agar supplemented with chloramphenicol (25 µg/mL). Single chloramphenicol-resistant colonies were picked, and genomic DNA was extracted. Successful replacement of nirA by the cat cassette was confirmed by diagnostic PCR using the nirA flanking primers (nirA-up-F / nirA-dn-R) and cat-specific primers (cat-up-F / cat-dn-R). Finally, long-range PCR spanning the entire Up–cat–Dn region followed by Sanger sequencing verified precise allelic exchange and confirmed that no additional mutations or rearrangements had occurred.

Co-incubation Experiments of Ileocecal Contents and Bacteria

The fresh ileocecal contents of mice were collected and weighed, then diluted with a 4-fold volume of PBS, ground, and vigorously shaken to ensure thorough mixing. Centrifuged at 500 g to obtain the upper suspension after centrifugation and discarded the bottom fecal residue. A mixture consisting of equal parts mouse ileocecal content extract and *C. perfringens* culture solution was prepared as the fecal microbiota co-incubation mixture. DT-109 was added to achieve a final

concentration of 1mM (the control group received an equivalent amount of ddH_2O), after which the mixture was incubated in an anaerobic environment for 24 h. Subsequently, it was frozen and stored at -80℃ for subsequent analyses.

Bacterial DNA Extraction, PCR, and Agarose Gel Electrophoresis

DNA extraction from the co-incubation mixture was performed using the Bacterial DNA Kit (Omega bio-tek, D3350-01, Guangzhou, China) according to the manufacturer's instructions. To assess the impact of DT-109 on the growth of *C. perfringens* and the ammonia-producing capacity of the co-incubation mixture, genes were amplified via PCR. The resulting amplified DNA fragments were separated on a 1.5% agarose gel (Yesen, 10208ES60, Shanghai, China), and relative DNA quantities were determined by analyzing gray values associated with each band.

Assessing the Efficacy of Phages Against *C. Perfringens* in a Mouse Model

A phage library was isolated from the feces of mice post-gavage with *C. perfringens*, containing strains potentially infectious to *C. perfringens*. The library was prepared by suspending the fecal matter in pre-cooled SM buffer at a 1:10 ratio, followed by centrifugation at 4°C and 5,000 rpm for 15 min to obtain the supernatant. This supernatant was subjected to a second centrifugation at 4°C and 13,000 rpm for 15 min to remove solid residues. The resulting supernatant was filtered twice through a 0.22 μm polyvinylidene fluoride (PVDF) filter to eliminate bacteria, resulting in the phage solution. To selectively cultivate phages capable of infecting *C. perfringens*, 1 mL of *C. perfringens* culture (OD_{600} = 0.3, grown to over 80%) was combined with 12 mL of 3X BHI medium and 23 mL of phage solution and cultured anaerobically overnight. The culture was then centrifuged at 4°C and 4,000 rpm for 10 min, and 5 mL of the supernatant was filtered through a 0.22 μm filter. This filtrate was combined with 1 mL of bacterial culture and 3 mL of 3X BHI and cultured overnight. This amplification process was repeated twice to obtain anti-*C. perfringens* phages. For virulence determination, six-well plates containing BHI solid medium were inoculated with *C. perfringens* culture and incubated until approximately 80% growth was achieved. Subsequently, 1 mL of diluted phage solution was added to each well. Following further incubation, the plates were examined for the presence of lytic plaques or morphological changes in the bacteria. The appropriate concentration was determined by measuring plaque-forming units (PFU). The phage solution was stored at 4°C and used within six months. Two groups of mice were utilized. The first group was administered *C. perfringens* (10^8 CFU/mL, 200 μL per mouse, twice per week). The second group received the same *C. perfringens* administration in

conjunction with phages (200 μL per mouse per dose at $1x10^8$ PFU/mL).

Detection of DT-109

Bacterial solution (20 μL) or bacterial precipitate (20 mg) was collected in 1.5mL EP tubes. Methyl alcohol (80 μL) comprised of 5 μM ^{13}C-labeled IS-DT109 was added to EP tubes. The bacteria were vortexed and sonicated at 4-8 ° C to obtain their contents, and macromolecular proteins were precipitated with methanol. Then the supernatant was recovered following centrifugation at 20,000 g at 4 °C for 10 min. Supernatants (5 μL) were analyzed by injection onto an Intrada Amino Acid (100×3 mm, 3 μm) UPLC column (IMTAKT corporation, Kyoto, JAPAN) at a flow rate of 0.4ml/min using an LC-20AD Shimadzu pump system, SIL-20AXR auto-sampler interfaced with an API 4500Q-TRAP mass spectrometer (SCIEX, Framingham, MA), and the samples were maintained in an autosampler at 4℃ during analysis. A discontinuous gradient was generated to resolve the analytes by mixing solvent A (0.01% formic acid water) with solvent B (0.01% formic acid in acetonitrile) at different ratios starting from 5% to 95% B in 1min, keeping 95% B in 2min, 95% to 5% B in 0.1min and then keeping 5% B in 1.5min. To optimize the MS parameters, the standard solutions were infused directly into the mass spectrometer in the positive ionization mode. Precursor and characteristic product-ion transitions of DT109 at m/z 246.2→132.1, IS-DT109 at m/z 248→132.1. The MS parameters were as follows: ion spray voltage at 4500 V; source temperature at 500°C; curtain gas and collision gas at 30 psi and 7 psi, respectively. All analyses were detected in multiple reaction monitoring (MRM) mode. All data acquisition, analysis, and processing were performed using the Analyst software (version 1.6.2, SCIEX). For NBD probes confirm DT109 uptake into *C. perfringens*, DT109–NBD was synthesized by selectively labeling the free N-terminus of DT109 with NBD-F in a mildly basic aqueous buffer, followed by quenching excess NBD-F using an amine scavenger. *C. perfringens* was cultured anaerobically in Reinforced Clostridial Medium until mid-log phase, then harvested, washed twice with PBS, and resuspended to an OD_{600} of approximately 0.2. Bacterial suspensions were incubated with either NBD-F (20 μM), DT109-NBD (20 μM), or Vancomycin-NBD (20 μM) in the presence of vancomycin at 37 °C under anaerobic and light-protected conditions for 1 hour. Following incubation, samples were washed three times with PBS, resuspended, applied onto glass slides, and covered with coverslips. Confocal laser scanning microscopy was conducted under identical imaging parameters across all experimental conditions.

Isolation and *In Vitro* Culture of CD8$^+$ T cells

Mouse $CD8^+$ T cells were isolated using the MagniSort™ Mouse $CD8^+$ T Cell Enrichment Kit (Invitrogen, 8804-6822-74) as follows: Under sterile conditions, the mouse spleen was removed and crushed by applying pressure. The homogenized spleen was then filtered through a 40 μm screen, and the filtrate was collected into a 50 mL centrifuge tube. This suspension was centrifuged at 4°C and 320 g for 5 min, after which the supernatant was discarded. The spleen cells were resuspended in 2 mL of red blood cell lysis buffer and thoroughly dispersed with a pipette. The mixture was incubated at room temperature for 2 min, followed by the addition of 4 mL of PBS to stop the reaction. The suspension was centrifuged again at 4°C and 320 g for 5 min, and the supernatant was discarded.

A single-cell suspension of lymphocytes at a concentration of 1×10^8 cells/mL was prepared using cell separation buffer. To this, 20 μL of MagniSort™ enrichment antibody per 100 μL of cells was added, mixed thoroughly, and incubated at room temperature for 10 minutes. The cells were washed with separation buffer, and then 20 μL of MagniSort™ negative selection beads per 100 μL of cells was added, mixed thoroughly, and incubated at room temperature for 5 min. The supernatant was poured into a new 12 x 75 mm, 5 mL tube using a magnet in a continuous motion, and the inverted tube was held for 1 second before returning it to the upright position. The tube containing bound cells was removed from the magnet and discarded. The untouched, negatively selected cells in the new tube were ready for use.

The isolated $CD8^+$ T cells were resuspended at 1×10^6 cells/mL in complete CTS™ OpTmizer™ T-Cell Expansion SFM (Gibco, A1048501), supplemented with Anti-CD3 (1 μg/mL), Anti-CD28 (1 μg/mL), and IL-2 (10 ng/mL), and incubated at 37°C in a humidified atmosphere of 5% CO_2. For ammonia treatment, the concentrations of ammonium chloride in the medium were 0 and 100 μM.

Adoptive transfer of auto-aggressive CD8 T cells into mice

Transfection of siRNAs for FosB and control sRNA (scrambled RNA), purchased from Invitrogen, was performed for $CD8^+$ T cells *in vitro* culture using Lipofectamine 3000 reagent, Invitrogen, according to the manufacturer's protocol. The $CD8^+$ T cells were treated with ammonia (100 μM) or vehicle for 24 h, then washed and adoptively transferred (4×10^6 cells per mouse) into nude mice.

Quantitative Real-time PCR and ChIP-qPCR

Total RNA was extracted from $CD8^+$ T cells using Trizol reagent, and

subsequently reverse transcribed into cDNA with Evo M-MLV RT Reaction Mix (AG Bio, Changsha, China, AG11728). The resulting cDNA, together with primers and SYBR green master mix (AG Bio, AG11701), was assembled and subjected to Real-Time PCR analysis using a Bioer Technology Real-Time PCR System (Hangzhou, China, FQD-96C). The fold-change of gene expression was determined using the ΔΔCt method, with GAPDH serving as the reference housekeeping gene.

The ChIP assay was executed using the ChIP Kit (BersinBio, Guangzhou, China, Bes5001). In brief, $CD8^+$ T cells were cross-linked, lysed to disrupt cell membranes, and chromatin was sheared. The fragmented DNA was then incubated with an anti-FosB antibody (Cell Signaling Technology, Danvers, Massachusetts, USA, Cat#2251), while immunoglobulin G (IgG) served as the negative control antibody. The immunoprecipitated chromatin underwent quantitative PCR analysis with the SYBR Green master mix (AG Bio, AG11701). Primer pairs designed for ChIP-qPCR targeted the FosB binding site within the CCL5 promoter or enhancer regions. Data from both FosB immunoprecipitation (IP) and control IP were expressed as enrichment relative to the DNA input.

References

1. Jia X, Song E, Liu Y, Chen J, Wan P, Hu Y, et al. Identification and multicentric validation of soluble CDCP1 as a robust serological biomarker for risk stratification of NASH in obese Chinese. *Cell Rep Med.* 2023;4(11):101257.
2. Qu P, Rom O, Li K, Jia L, Gao X, Liu Z, et al. DT-109 ameliorates nonalcoholic steatohepatitis in nonhuman primates. *Cell Metab.* 2023;35(5):742-57 e10.
3. Gutierrez-de-Juan V, Lopez de Davalillo S, Fernandez-Ramos D, Barbier-Torres L, Zubiete-Franco I, Fernandez-Tussy P, et al. A morphological method for ammonia detection in liver. *PLoS One.* 2017;12(3):e0173914.
4. Shen H, Zhou L, Zhang H, Yang Y, Jiang L, Wu D, et al. Dietary fiber alleviates alcoholic liver injury via Bacteroides acidifaciens and subsequent ammonia detoxification. *Cell Host Microbe.* 2024;32(8):1331-46 e6.
5. Miao YR, Zhang Q, Lei Q, Luo M, Xie GY, Wang H, et al. ImmuCellAI: A Unique Method for Comprehensive T-Cell Subsets Abundance Prediction and its Application in Cancer Immunotherapy. *Adv Sci (Weinh).* 2020;7(7):1902880.
6. Gao Y, Zhang G, Jiang S, and Liu YX. Wekemo Bioincloud: A user-friendly platform for meta-omics data analyses. *Imeta.* 2024;3(1):e175.

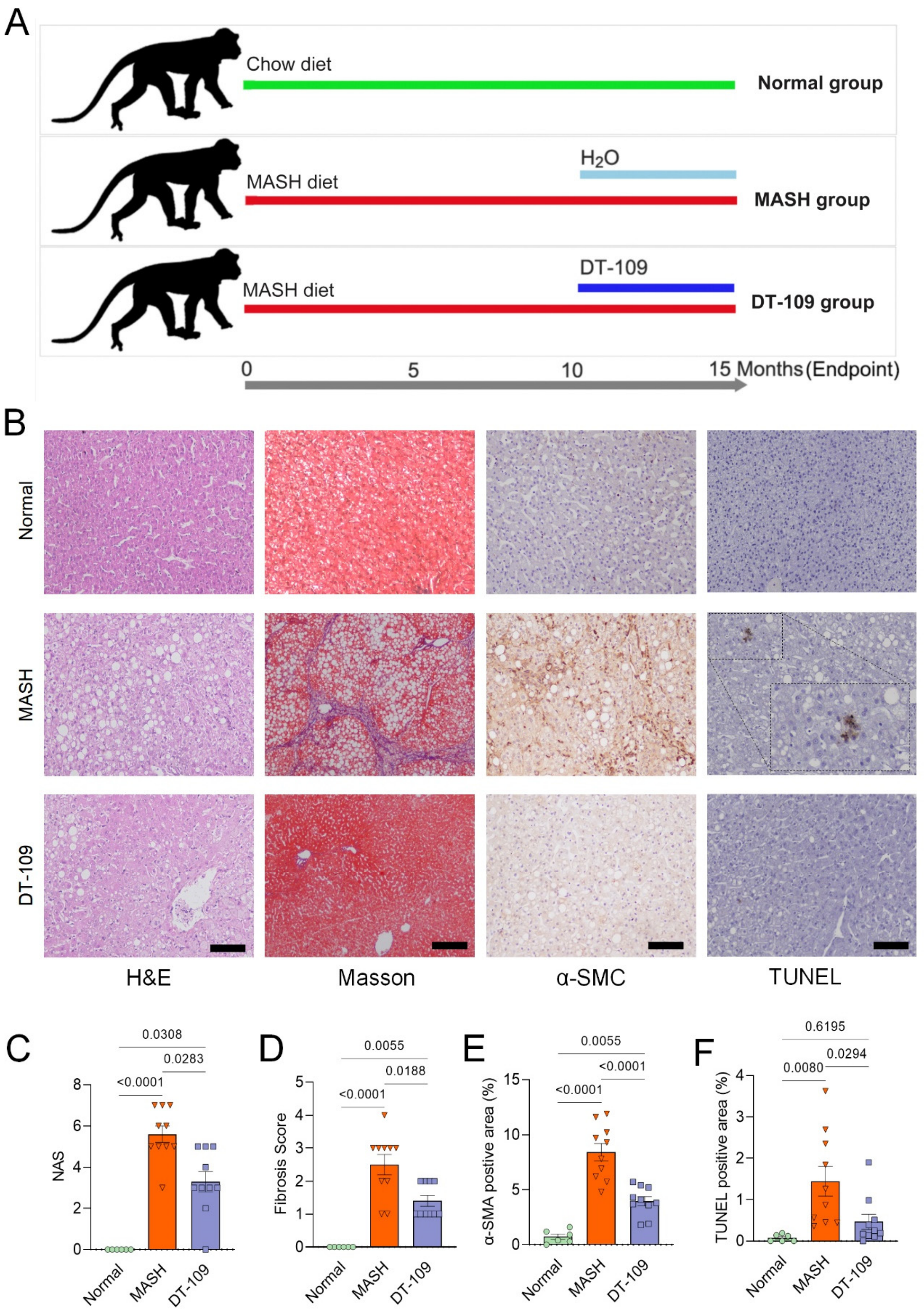

Fig. S1. The experimental design and pathological verification of NAFLD activity score and fibrosis in monkey livers.

(A) Schematic representation of experimental design. Monkeys were fed a chow diet (n=6) or MASH diet (n=20) for 10 months. After confirming the MASH phenotype, the monkeys fed

with the MASH diet were randomly divided into two groups, named the MASH group (n=10) and the DT-109 group (n=10), respectively. The DT-109 group received DT-109 daily via gavage at a dose of 150 mg/kg/day for 5 additional months while continuing on the MASH diet, whereas the MASH group received water. (B) Histology of monkey livers using H&E, Masson staining, α-SMA immunohistochemistry, and TUNEL assay (detects apoptotic cells) at the endpoint (scale bars, 100 μm). NAFLD activity score (NAS) (C) and fibrosis score (D) at the endpoint. The NAS is the sum of steatosis, hepatocellular ballooning, and lobular inflammation scores assessed by H&E histology. The fibrosis score was assessed by Masson staining. Quantification of α-SMA (E) and TUNEL-positive (F) in monkey livers. Data are presented as means ± SEM. Statistical differences were analyzed by the Kruskal-Wallis test, followed by Dunn’s post hoc test, or one-way ANOVA, followed by Dunnett's post hoc test, depending on normality tests.

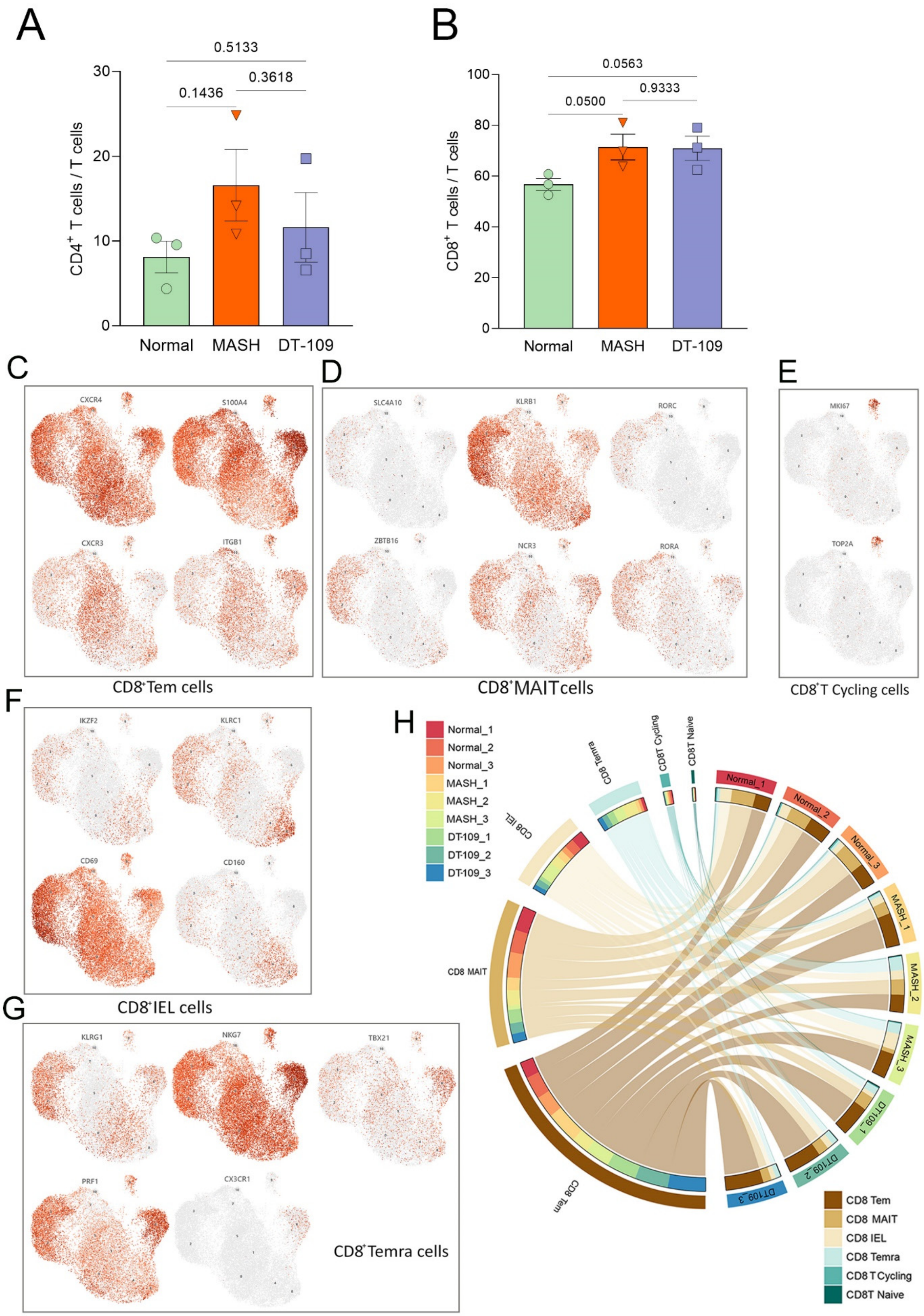

Fig. S2. Characterization of CD8+ T sub-type cells in Monkey Livers Based on scRNA-seq Analysis.

Histogram plot showing percentage of $CD4^+$ T cells (A) and $CD8^+$ T cells (B) out of total T cells in the monkey livers among the normal group, MASH group, and DT-109 group based on scRNA-seq analysis (n=3 in each group, each data point represents an individual animal). Statistical differences were compared by one-way ANOVA followed by Dunnett's post hoc test (A, B). UMAP visualization and marker gene expression in $CD8^+$ Tem cells (C), $CD8^+$ MAIT cells (D), $CD8^+$ IEL cells (E), $CD8^+$ Temra cells (F), and $CD8^+$ T cycling cells (G). (H) Circos plot showing the percentage of $CD8^+$ T sub-types cells (including $CD8^+$ Temra cells, $CD8^+$ Tem cells, MAIT cells, $CD8^+$ IEL cells, $CD8^+$ T Cycling cells, and $CD8^+$ T Naive cells) out of total $CD8^+$ T cells in the monkey livers among the three groups in an individual sample based on scRNA-seq analysis.

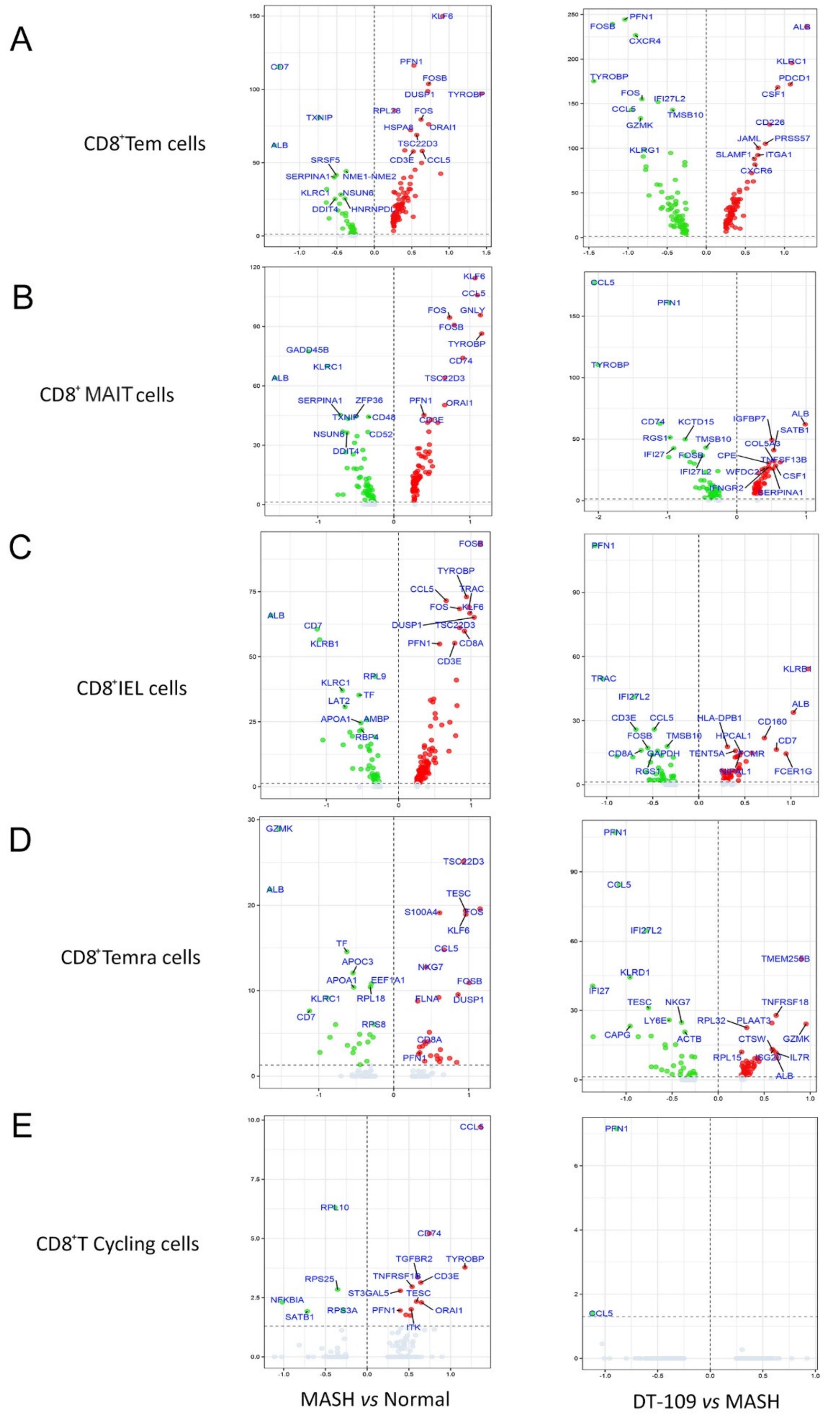

Fig. S3. DEGs of CD8$^+$ T sub-type cells in Monkey Livers Based on scRNA-seq Analysis. Scatterplot of different gene expressions analyzed by DESeq2 in CD8$^+$ Tem cells (A), CD8$^+$

MAIT cells (B), CD8+ IEL cells (C), CD8+ Temra cells (D), and CD8+ T cycling cells (E) revealed by scRNA-seq of CD8+ Tem cells from MASH and normal monkey livers (left), and DT-109 and MASH monkey livers (right). The representations are as follows: x-axis, logFC; y-axis, -log10 of FDR. The significant DEGs with both satisfying values are in green (down-regulated) or red (up-regulated) dots.

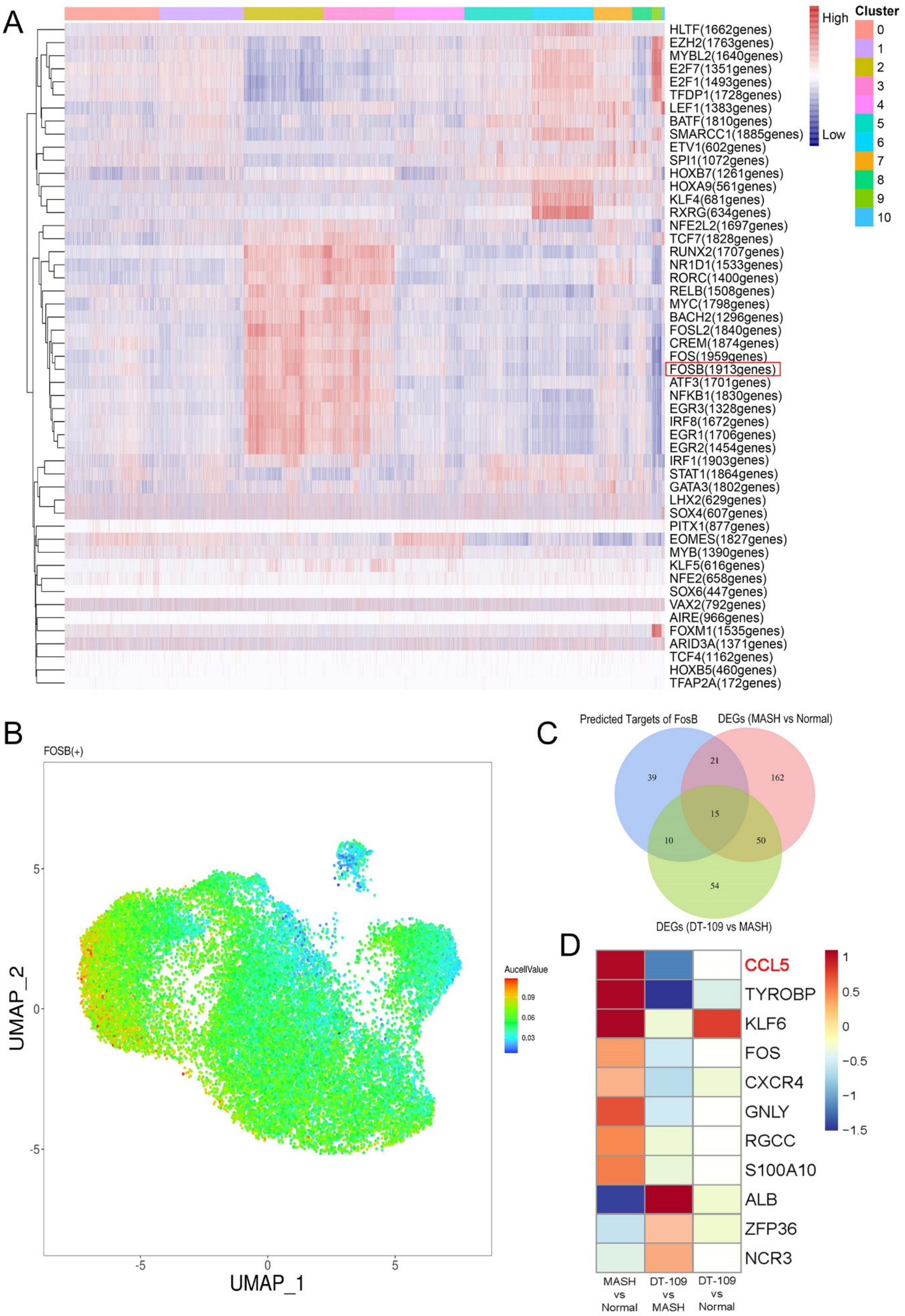

Fig. S4. Transcription Factor Activity Analysis in CD8+ T Cells of Monkey Livers Using SCENIC.

(A) Heatmap of activity of transcription factors in the CD8$^+$ T cell of monkey livers using SCENIC. Columns represent individual cells, and the color legend at the top displays clusters, and the cluster labels correspond to those used in Fig. 1F. The number in parentheses after the gene name indicates the number of regulated genes. The stronger the regulation intensity of transcription factors, the closer the color is to red; the weaker, the closer to blue. Gene names are marked with red boxes, indicating that transcription factors belong to differentially expressed genes (FOSB is differentially expressed between the comparisons: MASH vs normal, and DT-109 vs MASH; and STAT is differentially expressed between the MASH vs normal groups). (B) UMAP visualization of AUCell regulatory intensity of transcription factor FosB. Scale bars, AUCell Score. (C) Venn plot showing target genes of FosB in the CD8$^+$ T cell and the DEGs in the two comparisons (MASH vs normal, and DT-109 vs MASH) in the CD8$^+$ T cell. (D) Heatmap of the overlap genes (in Fig. S4C) in the three comparisons (MASH vs normal, DT-109 vs MASH, and DT-109 vs normal) in the CD8$^+$ T cells. White represents $p \geq 0.05$. Scale bars, $\log_2$FC.

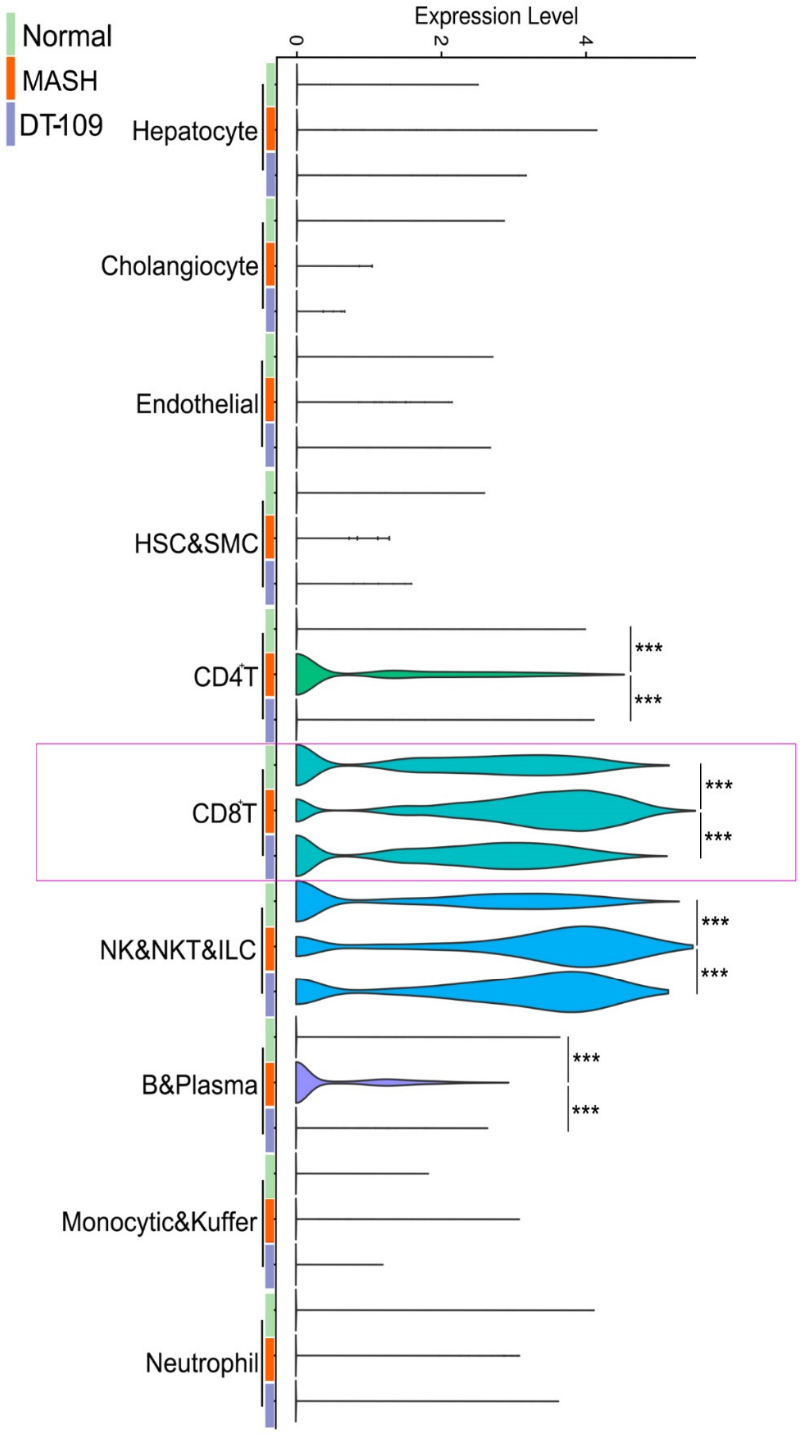

Fig. S5. Violin diagram of CCL5 expression level in all cell populations of monkey liver among the three groups.

Gene expression levels in each cell type were analyzed by DESeq2. ***FDR < 0.0001, compared with MASH group. CCL5 expression in $CD8^+$ T cells was marked in a purple box.

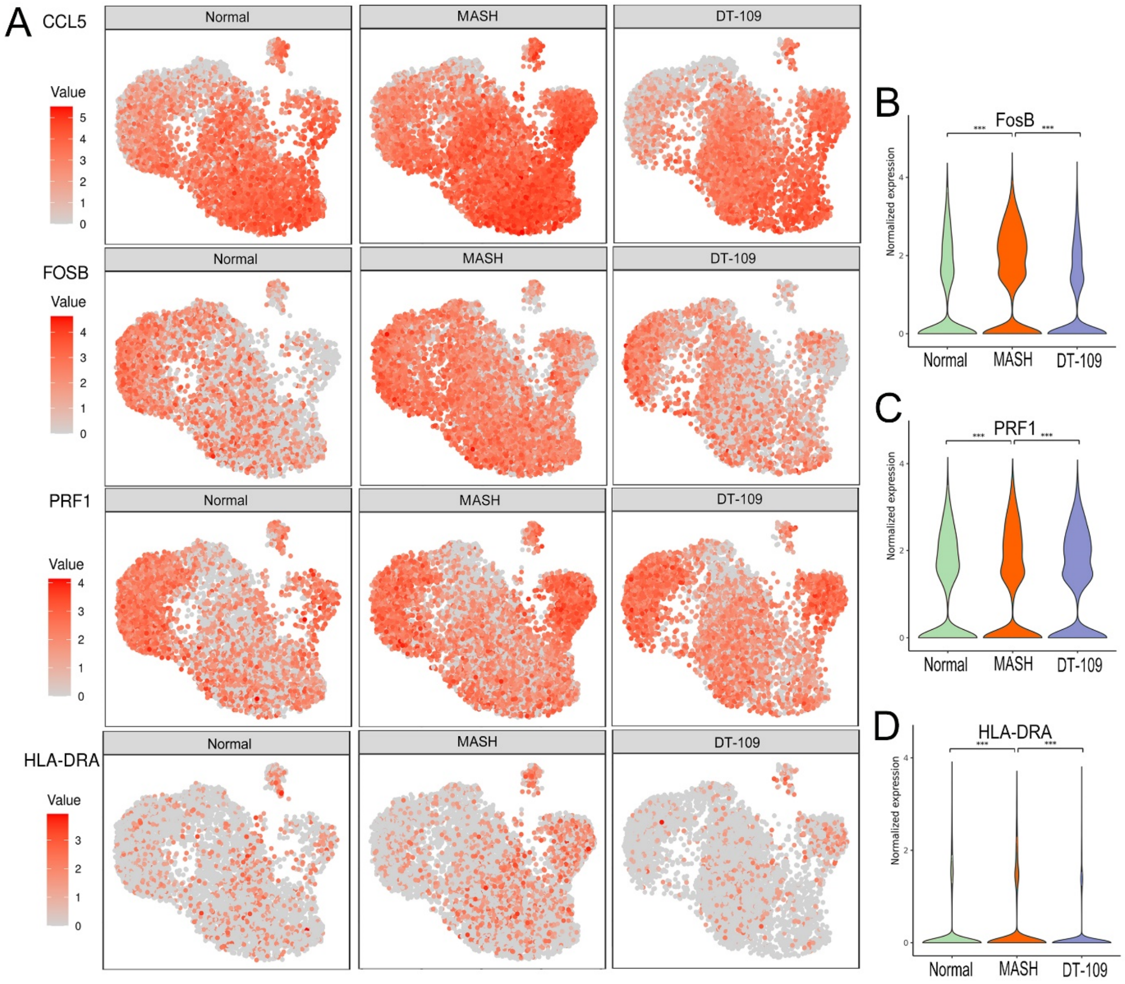

Fig. S6. *FosB*, *CCL5*, *PRF1*, and *HLA-DRA* Expression in Liver CD8$^+$ T Cells from Monkey.

UMAP visualization (A) of FOSB, CCL5, PRF1, and HLA-DRA expression level in the CD8$^+$ T cells of monkey liver among the three groups. Scale bars, gene expression level; the higher the expression level, the closer the color is to red; the lower the level of expression, the closer the color is to gray. Violin diagram of FOSB (B), PRF1 (C), and HLA-DRA (D) expression level in the CD8$^+$ T cells of monkey liver among the three groups. ***FDR < 0.0001, compared with the MASH group. Statistical differences were analyzed by DESeq2.

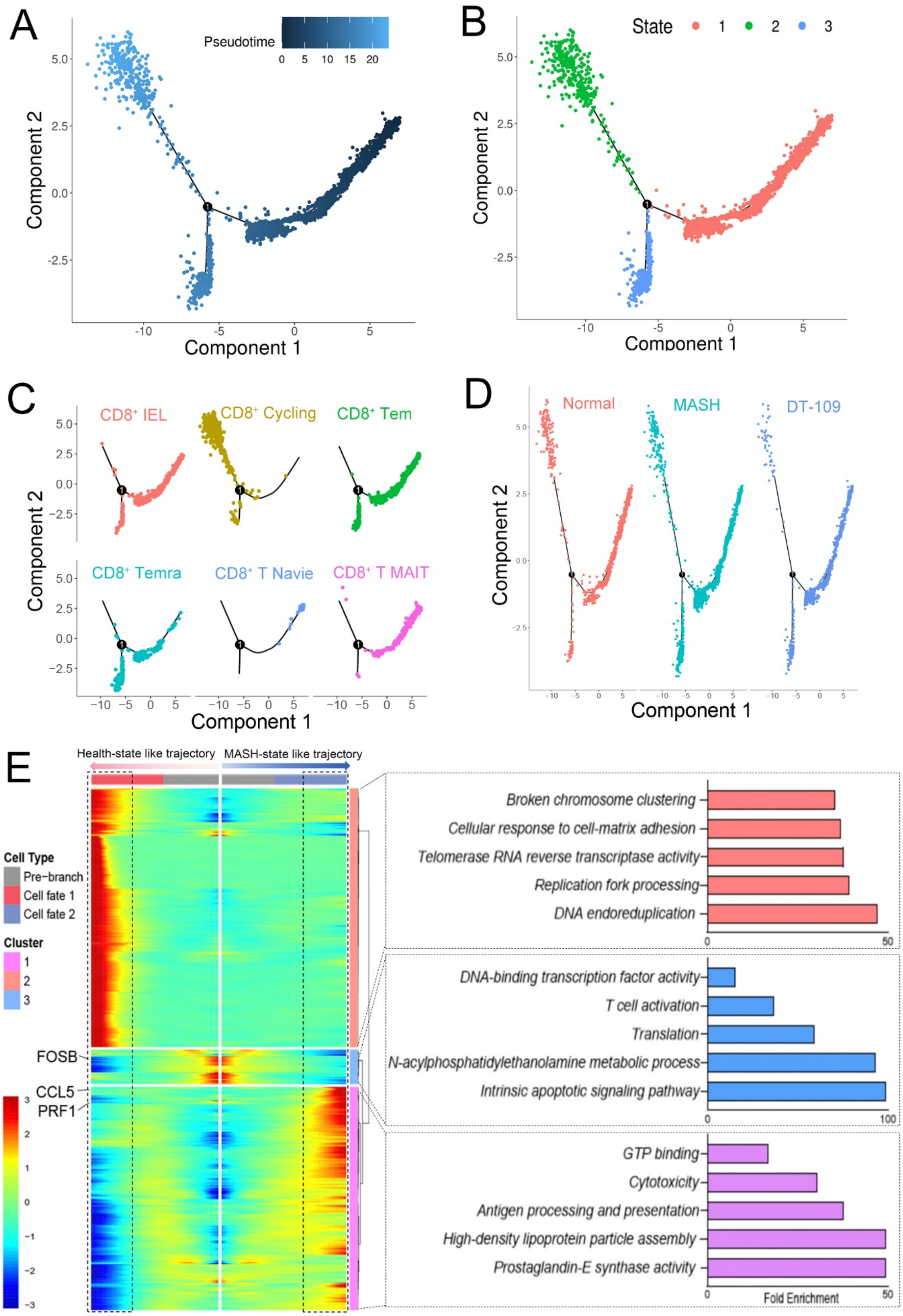

Fig. S7. Pseudotime Analysis Reveals CD8⁺ T Cell Differentiation in Monkey Livers.

Pseudotime analysis of CD8⁺ T cells in monkey livers was performed by Monocle 2, and the

pseudotime trajectory of the CD8$^+$ T cells (A), and revealed 3 different cell states (states 1~3). The distributions of cell states were presented along with pseudotime flows (B). Visualization of CD8$^+$ T cells differentiation trajectory by cell identity (C) and group identity (D). Each dot is a cell. (E) Trajectory inference and gene expression dynamics. The MASH state-like trajectory (blue) and health state-like trajectory (red) were inferred from all CD8+ T cells and represent predominant cell fates driven by their respective phenotypic states. Gene expression dynamics were calculated along these two trajectories. The heatmap (left) shows the relative expression of the top 1,000 DEGs (categorized into three clusters) along the inferred pseudotime. Trajectory reconstruction identified three segments: a pre-branch (before bifurcation), a cell fate 1 branch, and a cell fate 2 branch. The colored bars atop the heatmap correspond to cells primarily belonging to the health state-like (red) or MASH state-like (blue) trajectory. Enrichment analysis (right) of biological processes for the DEGs in the three clusters compares the two trajectories.

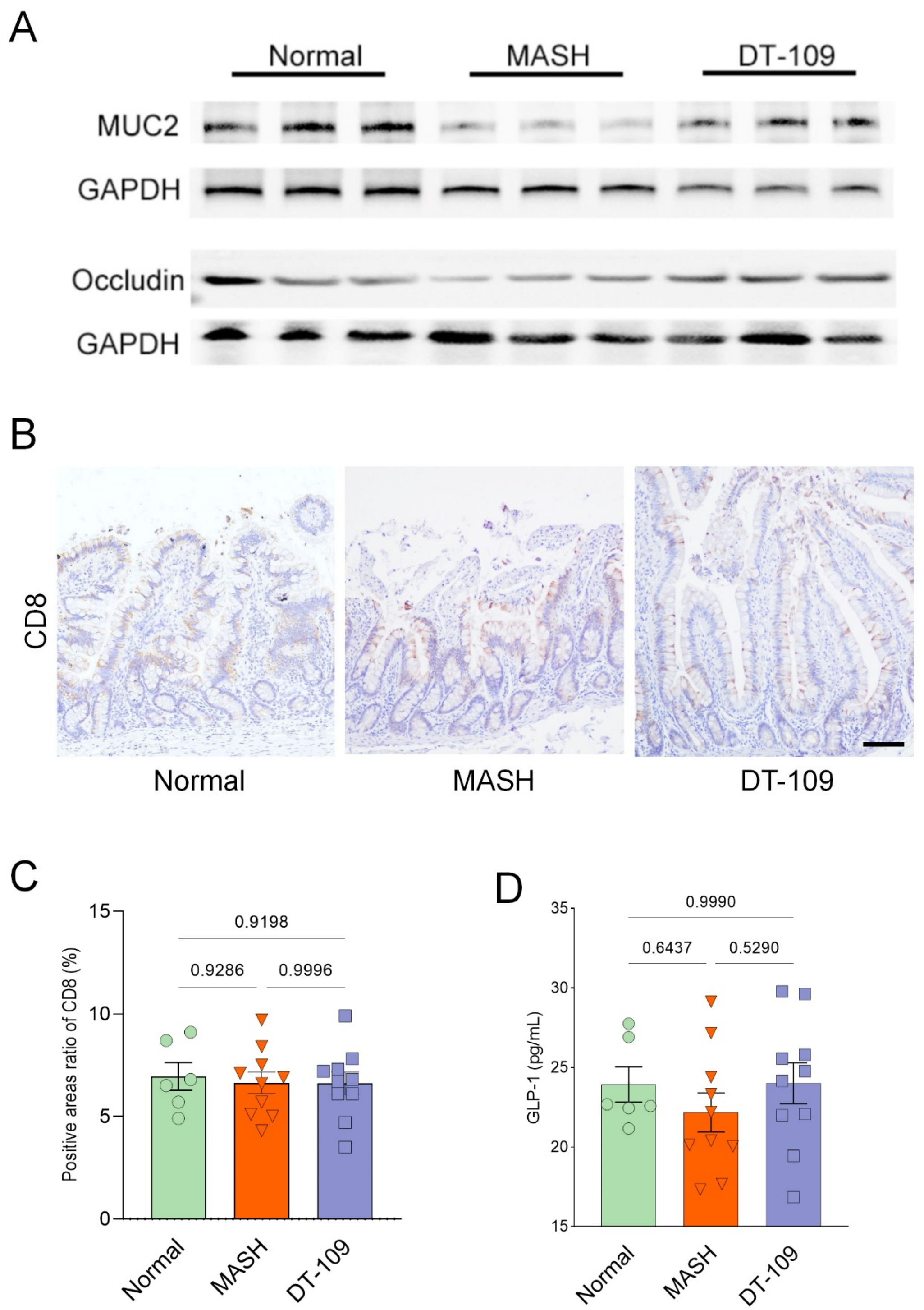

Fig. S8. Analysis of ileal and plasma biomarkers in monkeys with MASH. Samples are collected at the end stage from Normal, MASH, and DT-109 treated groups. (A) Representative Western blot images showing protein levels of MUC2 and Occludin in ileal tissues. (B) Representative immunohistochemical staining images demonstrating CD8-

positive cells in ileal tissues (scale bars are indicated). (C) Quantitative bar graph of the CD8-positive area in ileal tissues. (D) Plasma concentrations of GLP-1. Data are presented as mean ± SEM. Statistical differences were compared by one-way ANOVA followed by Dunnett's post hoc test (C, D).

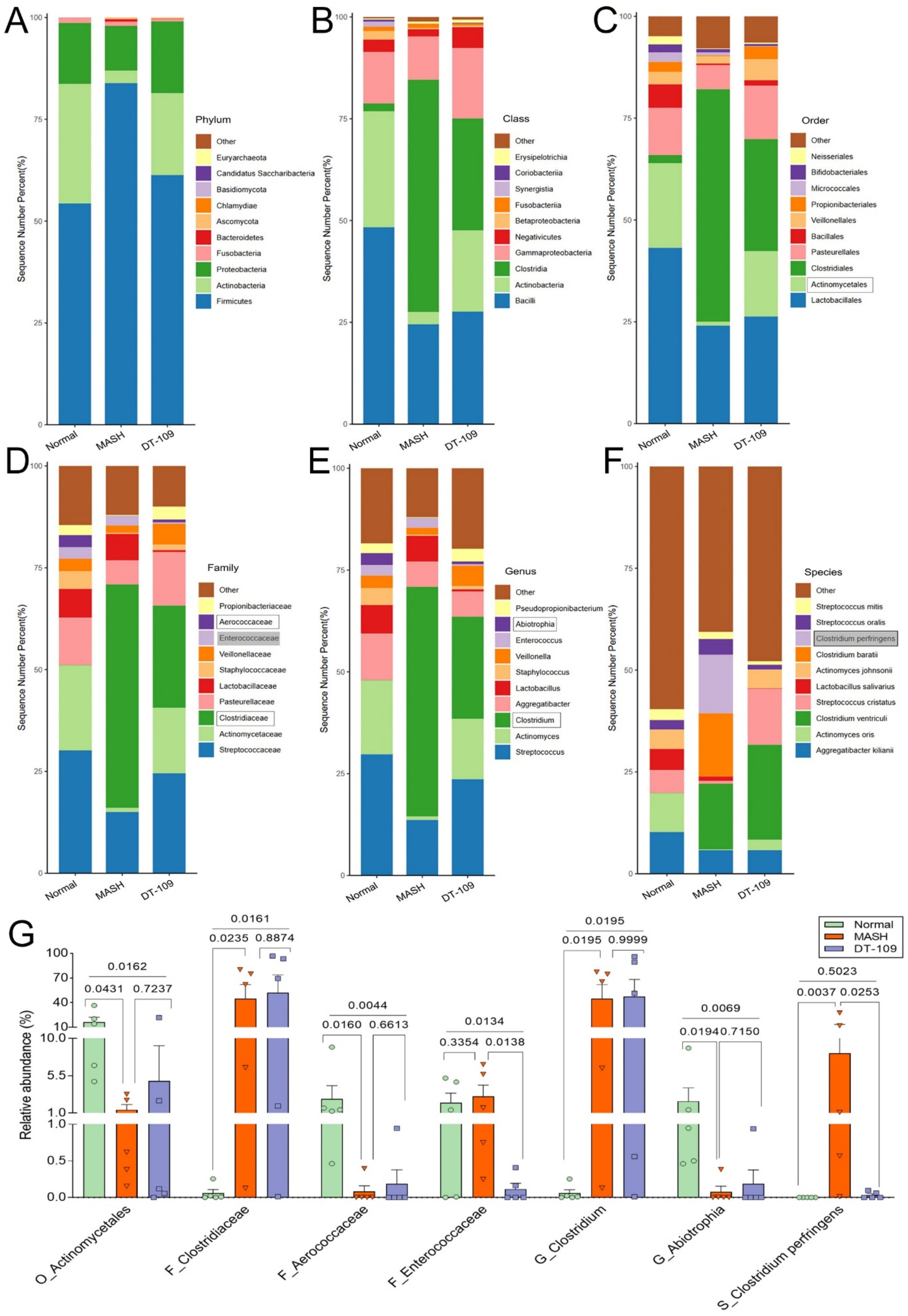

Fig. S9. Abundance analysis of microbiota in ileal contents from monkeys.

Top10 phylum (A), class (B), order (C), family (D), genus (E), and species (F) with the

highest abundance in the ileal contents of the monkeys among the three groups (n=5 in each group). The microbial names highlighted in solid boxes indicate the significantly different bacteria between the two groups (MASH vs. normal). Those highlighted with a gray background represent the significantly different bacteria between the two groups (DT-109 vs. MASH). Microbial names marked with both solid boxes and a gray background indicate significant differences in both comparisons. (G) Significantly different bacteria in the ileal contents among the normal group, MASH group, and DT-109 group at the endpoint based on metagenomic data of monkeys. Data are presented as means ± SEM. Statistical differences were analyzed by the Kruskal-Wallis test, followed by Dunn’s post hoc test (A-G).

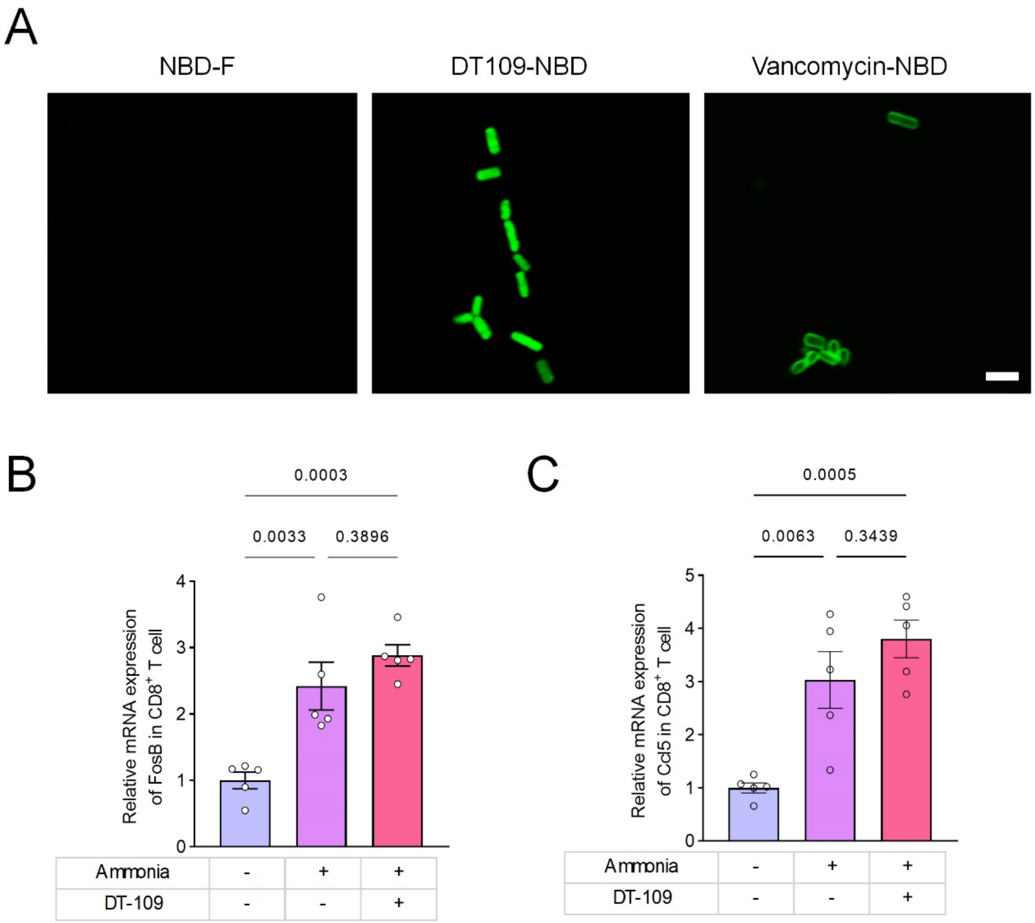

Fig. S10. DT-109 is internalized by bacteria and its lack of direct impact on ammonia-activated CD8⁺ T cells

(A) Representative confocal microscopy images of *C. perfringens* incubated for 1 hour with free NBD-F, DT-109-NBD, or Vancomycin-NBD. No fluorescence signal was detected in bacteria treated with free NBD-F. DT-109-NBD showed strong and uniform intracellular fluorescence. Vancomycin-NBD produced a distinct peripheral ring-like fluorescence pattern, consistent with labeling of the cell wall. Scale bars: 5 μm. (B, C) *In vitro* effect of DT-109 on gene expression in $CD8^+$ T cells. Mouse $CD8^+$ T cells were treated with ammonia (100 μM, 24 h) with or without co-intervention with DT-109 (100 μM). Quantitative PCR (qPCR) analysis of (B) FosB and (C) Ccl5 mRNA expression showed that DT-109 did not significantly alter the upregulation of either gene induced by ammonia. Data are presented as mean ± SEM; n = 5 biologically independent samples per group; Statistical differences were compared by one-way ANOVA followed by Dunnett's post hoc test (B,C).

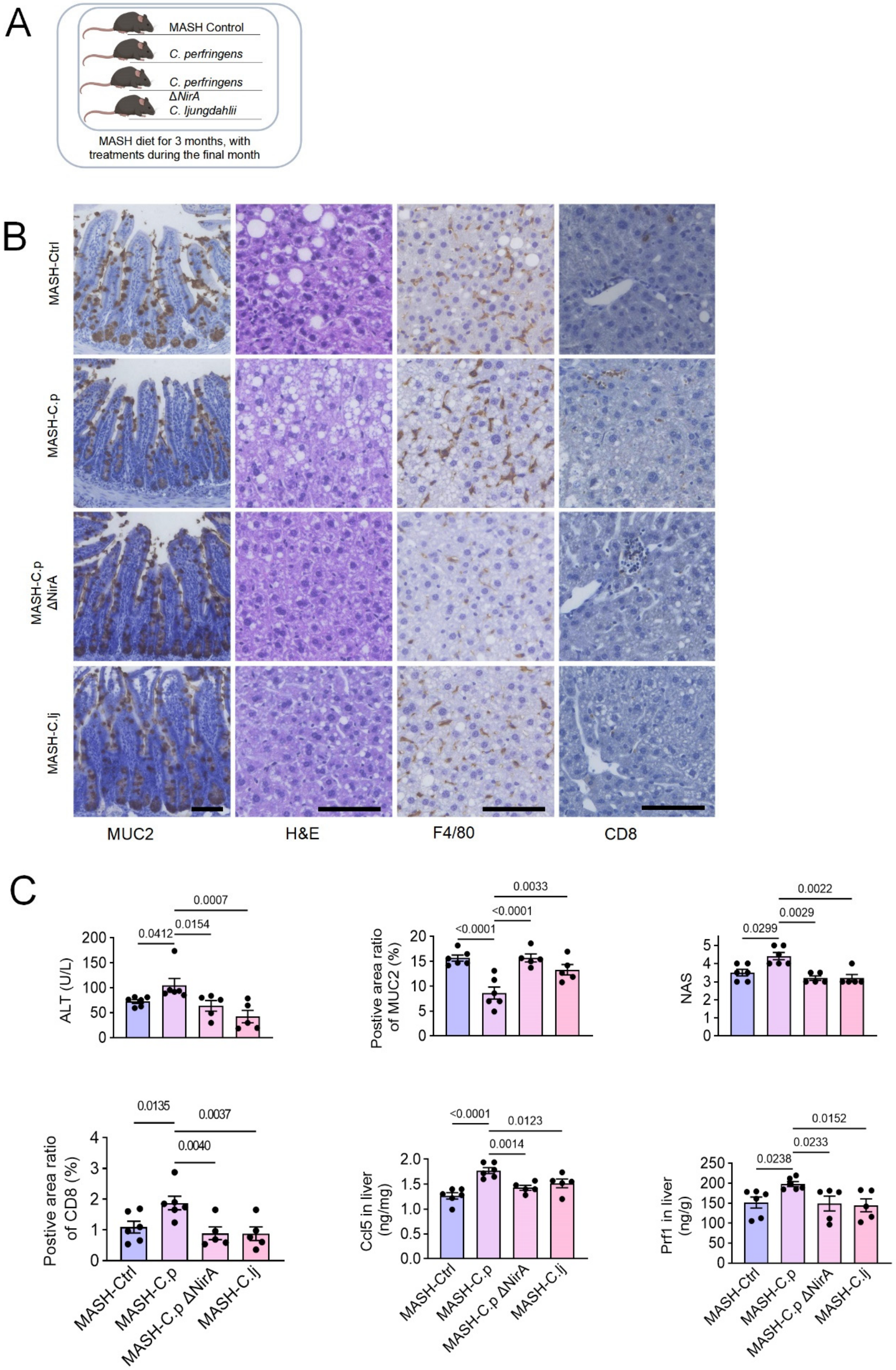

Fig. S11. Roles of *C. perfringens* in MASH progression and ammonia-induced hepatotoxicity. (A,B) Effect of specific bacterial colonization on MASH. (A) Schematic of

experimental design. C57BL/6 mice were fed a MASH-diet for 3 months. During the final month, mice were colonized with *C. perfringens* (MASH C.p), *C. perfringens* ΔNirA (MASH C.p ΔNirA), C. ljungdahlii (MASH C.lj), or remained as untreated controls (MASH Ctrl). Quantified endpoints: plasma ALT levels, ileal Muc2 mRNA expression, proportions of hepatic F4/80$^+$ macrophages and CD8$^+$ T cells, and hepatic Ccl5 and Prf1 concentrations. (B) Representative images corresponding to groups in (A): Ileal histology (AB-PAS staining), ileal MUC2 immunohistochemistry (IHC), hepatic histology (H&E staining), and hepatic IHC for F4/80 and CD8 (scale bars, 100 μm). Data (C) are presented as means ± SEM. Statistical differences were analyzed using the Kruskal–Wallis test followed by Dunn’s post hoc test for NAS, and one-way ANOVA followed by Dunnett's post hoc test for all others.

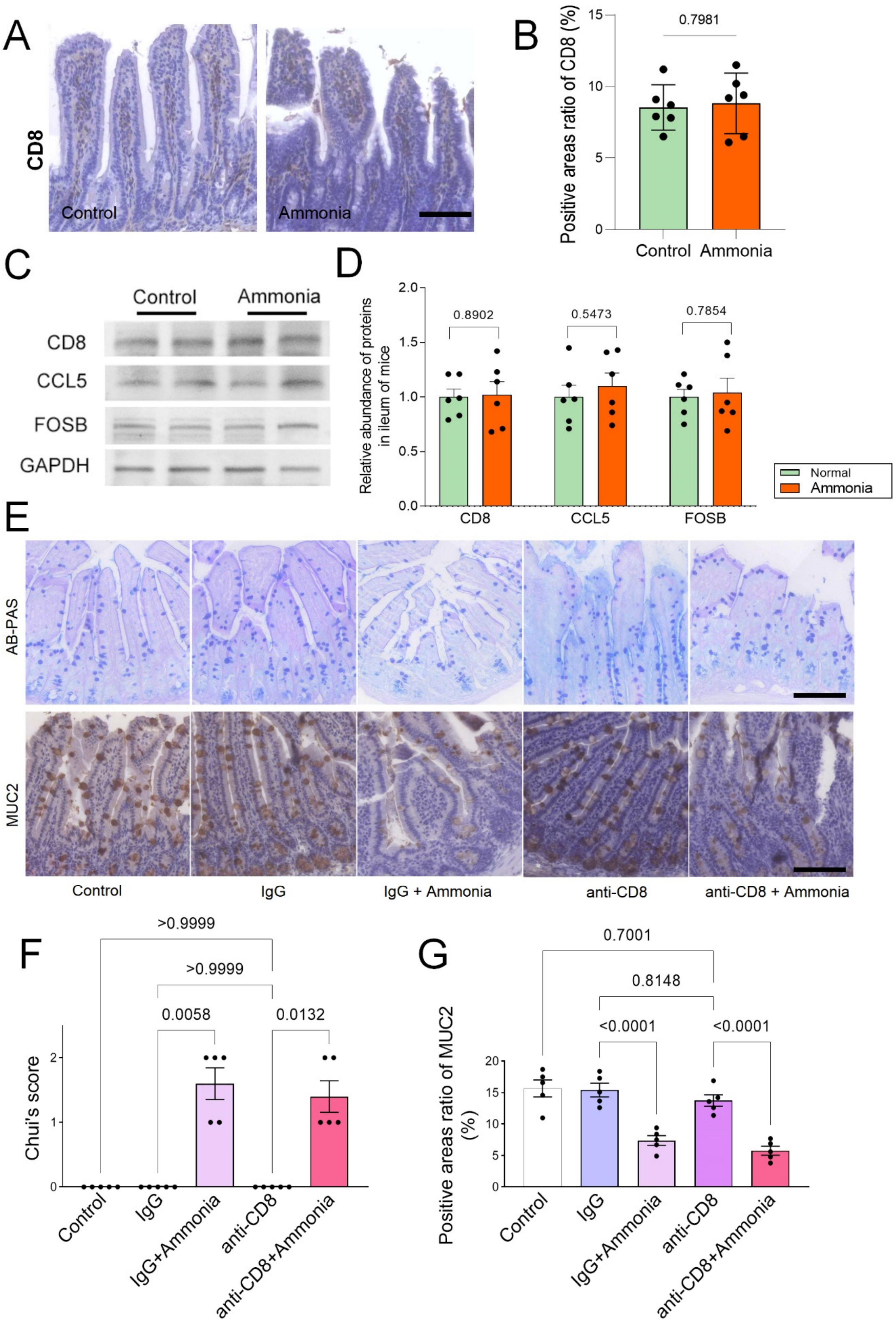

Fig. S12. Ammonium does not elevate intestinal CD8⁺ T cells and associated markers.

(A–D) C57BL/6 mice were treated with ammonium chloride (0.30 mg/g/day by oral gavage)

or water control for 8 weeks. (A) Representative immunohistochemistry images of $CD8^+$ T cells in the ileum. (B) Quantification of $CD8^+$ area percentage in ileal tissue. (C) Western blot of CD8, FosB, and CCL5 protein expression in ileal tissues. (D) Quantitative analysis of CD8, FosB, and CCL5 protein levels in the ileum based on panel C. (E–G) C57BL/6 mice were treated for 1 week as follows: control (no ammonia), control IgG (200 µg/mouse, i.v.), $CD8^+$ T cell–depleting antibody (200 µg/mouse, i.v.), control IgG plus ammonia (IgG + A), or $CD8^+$ T cell–depleting antibody plus ammonia (anti-CD8 + A). (E) Representative images of AB-PAS and MUC2 staining in ileal sections. Chui's score (F) and quantification of MUC2-positive area percentage (G) comparison among groups. Data are presented as mean ± SEM (B, D, F, G); Statistical differences were analyzed using unpaired t test (B, D), Kruskal-Wallis test followed by Dunn's post hoc test (F), one-way ANOVA followed by Dunnett's post hoc test (G).

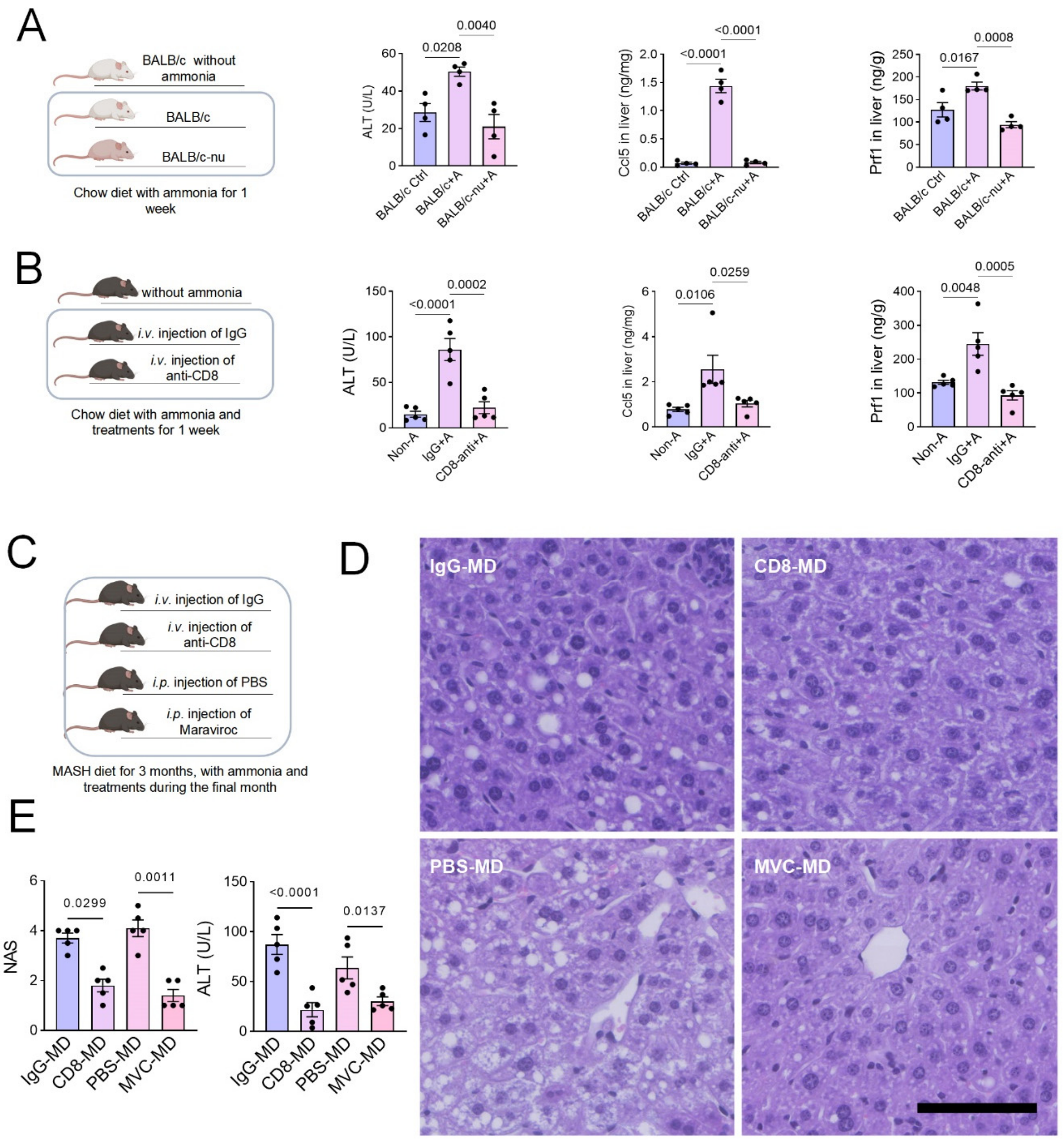

Fig. S13. Roles of CD8$^+$ T cells in MASH progression and ammonia-induced hepatotoxicity. (A) Role of T cells in ammonia-induced liver injury. Schematic of experimental design using BALB/c mice: Untreated controls (Ctrl), wild-type mice exposed to ammonia for 1 week (Balb/c+A), and T cell-deficient mice (BALB/c-nu) exposed to ammonia for 1 week (Balb/c-nu+A). Measured endpoints: plasma ALT levels, hepatic Ccl5 and Prf1 concentrations. (B) Specific role of CD8$^+$ T cells in ammonia-induced liver injury. Schematic of experimental design: C57BL/6 mice administered: no ammonia (Non-A), control IgG plus ammonia (IgG + A), or CD8$^+$ T cell-depleting antibody plus ammonia (CD8-anti + A). Plasma ALT levels, and hepatic Ccl5 and Prf1 concentrations in the three groups. (C) Therapeutic targeting of CD8$^+$ T cells/Ccl5 in MASH with superimposed ammonia challenge. Schematic of experimental design: C57BL/6 mice fed a MASH-diet for 3 months. During the final month,

mice received ammonia and one of the following treatments: IgG control (IgG), $CD8^+$ T cell-depleting antibody (CD8-anti), PBS control (PBS), or the CCR5 antagonist Maraviroc. Hepatic NAS and plasma ALT levels in the four groups. (D) Representative images of hepatic histology (H&E staining) for groups in (E) (scale bars, 100 μm). Data (A,C,D,E) are presented as means ± SEM. Statistical differences were analyzed using one-way ANOVA followed by Dunnett's post hoc test (A, B), Mann-Whitney U test (for NAS) or unpaired t test (for ALT) (E).

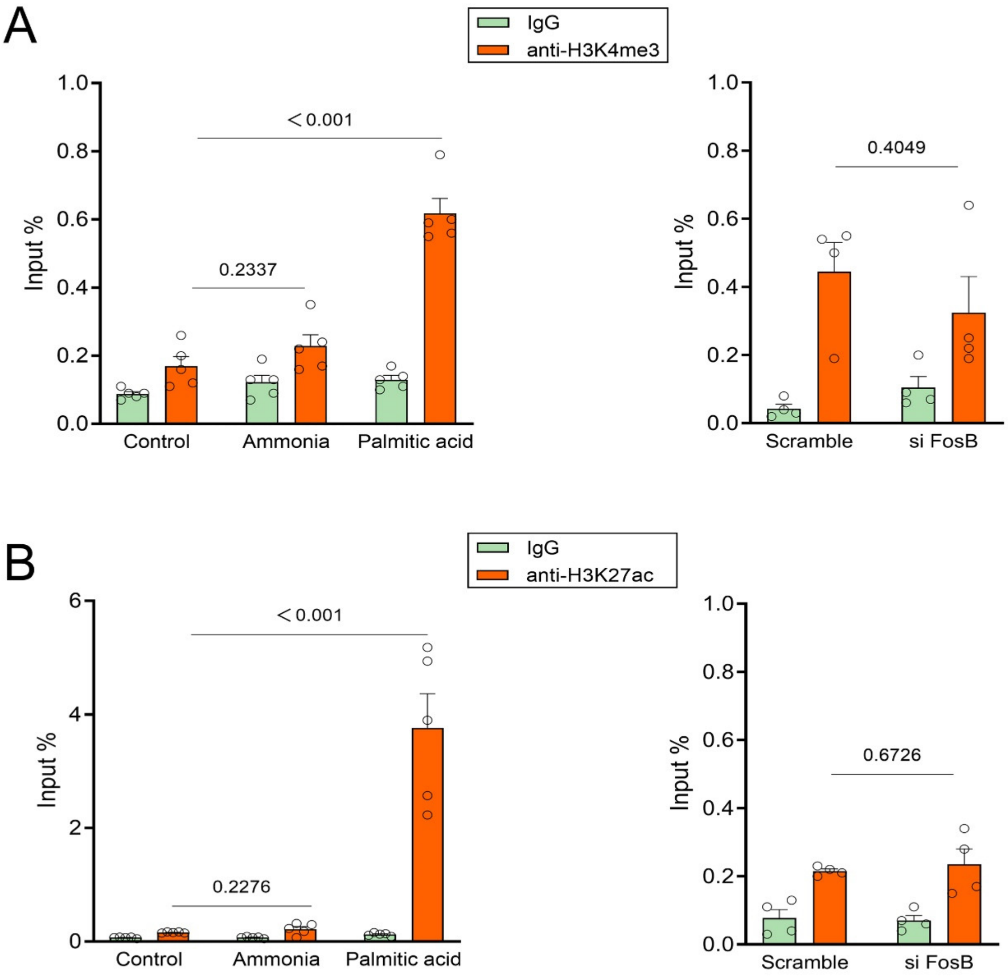

Fig. S14. H3K4me3 and H3K27ac enrichment at the *CCL5* gene promoter by ChIP-qPCR. (A) H3K4me3 enrichment in $CD8^+$ T cells after 24-h treatment with control, ammonia (100 μM), or palmitic acid (PA, 200 μM) (left panel), and in ammonia-treated cells transfected with scramble siRNA or *FosB*-specific siRNA (right panel). (B) H3K27ac enrichment under the same conditions as in (A). Data are presented as means ± SEM (n = 5); statistical significance was determined by unpaired t test.
