# Supplementary figures and images for: Metabolic dysfunction–associated steatohepatitis exacerbated by Clostridium perfringens–derived ammonia is attenuated by tripeptide DT-109
Source: J Clin Invest. 2026 May 12;136(13):e200522. doi: 10.1172/JCI200522 (PMC13318118; doi:10.1172/JCI200522)

Full unedited blots – Supplemental Figure 8A

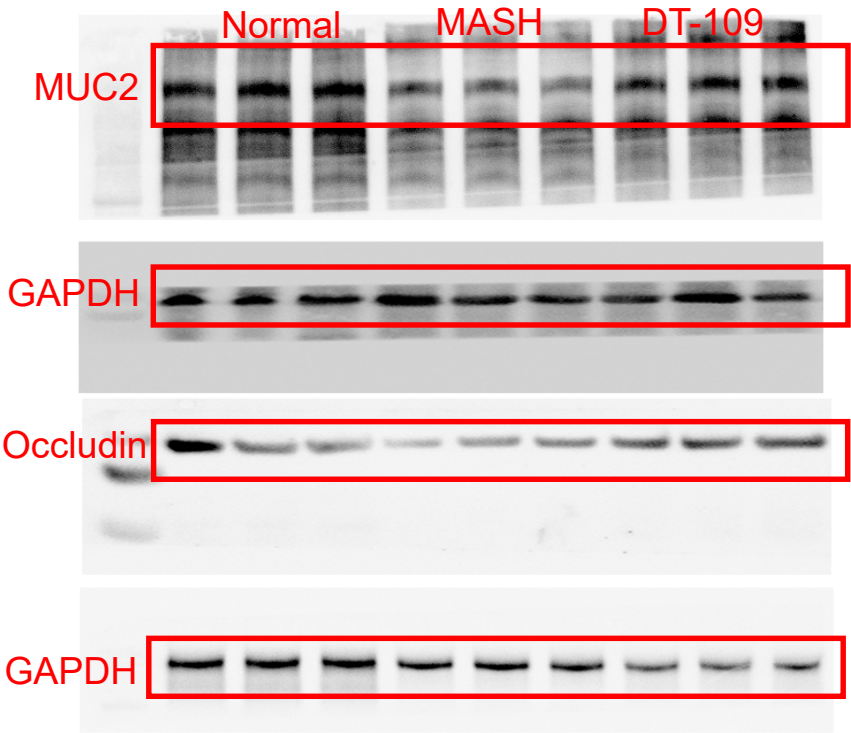

Full unedited blots – Supplemental Figure 12C

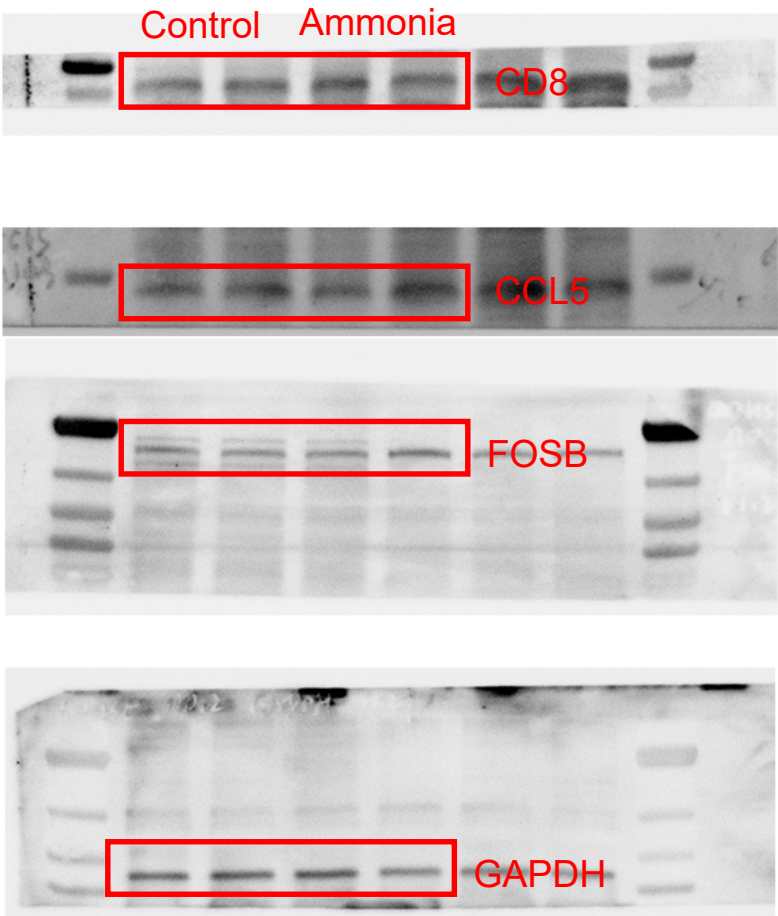

Supplement: Unedited blot and gel images [file jci-136-200522-s203.pdf]
